# SCS 6th Annual Meeting—EEVFA—11th International Congress of Biochemistry and Physiology of Exercise, Athens, Greece, 2023

**DOI:** 10.3390/sports12050126

**Published:** 2024-04-29

**Authors:** Pedro E. Alcaraz, Elena Marín-Cascales, Anthony J. Blazevich, Tomás T. Freitas, Olyvia Donti, Konstantinos Spyrou, Gregory C. Bogdanis

**Affiliations:** 1 UCAM Research Center for High Performance Sport, UCAM Universidad Católica de Murcia, 30107 Murcia, Spain; palcaraz@ucam.edu (P.E.A.); tfreitas@ucam.edu (T.T.F.); kspyrou@ucam.edu (K.S.); 2 Facultad de Deporte, UCAM Universidad Católica de Murcia, 30107 Murcia, Spain; 3 Strength & Conditioning Society, 30008 Murcia, Spain; a.blazevich@ecu.edu.au; 4 Centre for Exercise and Sports Science Research, School of Medical and Health Sciences, Edith Cowan University, Joondalup 6027, Australia; 5 NAR-Nucleus of High Performance in Sport, São Paulo 04753-060, Brazil; 6 School of Physical Education and Sports Science, National and Kapodistrian University of Athens, 17237 Dafne, Greece; odonti@phed.uoa.gr (O.D.); gbogdanis@phed.uoa.gr (G.C.B.)

**Keywords:** congress, performance, exercise, health, training, injury

## Abstract

On behalf of the Strength and Conditioning Society (SCS) and the Hellenic Society of Biochemistry and Exercise Physiology (EEVFA), we are pleased to present the abstracts of the SCS 6th Annual Meeting and EEVFA—11th International Congress of Biochemistry and Physiology of Exercise. The event was held at the Hellenic Olympic Committee headquarters in Athens, Greece, on 19–22 October 2023, and comprised several invited sessions from international and national speakers on a variety of topics related to biochemistry and exercise physiology, strength and conditioning practices and their application to health, injury prevention and sports performance. These included strength training in high-performance sports, sport science and training–competition load management in elite environments, biochemistry and exercise physiology and prescription, nutrition and biomechanics, among others. The conference also included different practical workshops conducted by renowned academics and practitioners on eccentric training, change of direction ability and strength and power training in professional team-sports, and ergospirometry and exercise prescription in specific populations. Finally, the event disseminated up-to-date strength and conditioning research by providing practitioners and researchers with the opportunity to present their most recent findings. In this regard, all abstracts of the communications presented at the SCS 6th Annual Meeting—EEVFA—11th International Congress of Biochemistry and Physiology of Exercise can be found in this Conference Report.

## 1. Introduction

The Strength and Conditioning Society (SCS) and the Hellenic Society of Biochemistry and Exercise Physiology (EEVFA) are pleased to introduce the abstracts of the SCS 6th Annual Meeting and the EEVFA—11th International Congress of Biochemistry and Physiology of Exercise, which was the first international event co-organized by two different societies. In accordance with the SCS’s and EEVFA’s vision and mission of disseminating high-quality evidence of the performance and health benefits of strength and conditioning practices worldwide, the 2023 Conference took place in Athens, Greece, on 19–22 October. The SCS 6th Annual Meeting—EEVFA—11th International Congress of Biochemistry and Physiology of Exercise, held at the Hellenic Olympic Committee headquarters in Athens, brought together more than 350 delegates from different areas of expertise (i.e., sports science, sports physiotherapy, sports nutrition, exercise physiology and biochemistry and sports medicine, among others), which provided ample opportunities to exchange and discuss the latest evidence on strength and conditioning practices from multiple perspectives. In a stimulating social and professional environment, practitioners and academics from different countries had the possibility of attending several thought-provoking invited sessions from international and national speakers on a variety of applied topics related to strength training in high-performance sports and in the elderly, sport science and load management in elite environments and the role of the Head of Performance in team-sports. Moreover, basic science-related topics such as biochemistry and exercise physiology and prescription, nutrition and biomechanics were also addressed. As in previous years, the conference also offered multiple practical workshops conducted by renowned academics and practitioners on eccentric training, change of direction ability in soccer, strength and power training in professional basketball, and ergospirometry and exercise prescription in chronic obstructive pulmonary disease. Also worth noting, the event fostered the dissemination of up-to-date strength and conditioning research by providing practitioners and researchers with the opportunity to present and discuss their latest findings, which can be found in the abstracts that compose this Conference Report. Finally, the SCS, in collaboration with EEVFA, recognized professional and academic excellence in the field of strength and conditioning and presented, for the first time, the “Female Strength and Conditioning Coach of the Year Award”, the “Male Strength and Conditioning Coach of the Year Award”, the “Emerging Strength and Conditioning Coach of the Year Award” and the “Strength and Conditioning Coach Career Achievement Award” to outstanding coaches, and the “Young Investigator Award” and the “Applied Science Award” to remarkable researchers.

## 2. Conference Abstracts

### 2.1. Comparison between the Effect of Aerobic and Resistance Exercise with Different Intensities on Post Exercise Blood Pressure Responses in Normotensive Men

Ramzi Al-horani ^1,^*, Tha’er Malkawi ^1^ and Rishad Al-Zoubi ^1^

^1^ Department of Exercise Science, Yarmouk University, Irbid, Jordan

* Correspondence: raalhorani@yu.edu.jo

**Abstract:** The variation in resting blood pressure among individuals engaging in aerobic and/or resistance exercise training might be due to the level of the acute drop of blood pressure after exercise. Therefore, this study aimed to compare the acute effect of resistance and aerobic exercise with different intensities on post-exercise blood pressure responses. Seven normotensive and physically active males participated in this study (age: 25.3 ± 6.2; Body mass index (BMI): 23.7 ± 2.6). Baseline blood pressure, heart rate (HR), heart rate reserve (HRR), maximum strength (1RM) of 6 resistance exercises were measured. Accordingly, participants performed two 40-min cycling sessions at 60% of HRR (C60) and 75% of HRR (C75), and two sessions of resistance exercise (6 exercises) at 60% 1RM (RE60) and 75% 1RM (RE75) in random order. After each exercise session, participants sat for 60 min, during which blood pressure, HR and the rate of perceived exertion (RPE) were measured at 0, 15-, 30-, 45-, and 60-min post-exercise. The anxiety state Y1 was obtained at 0-, 30-, and 60-min post exercise. Systolic blood pressure decreased significantly at 15–60 min post C75 and at 60 min post RE75. Diastolic blood pressure was decreased at 0-, 15-, and 30-min post RE60 and at 30 min post RE75 only, and not decreased after C60 and C75. Mean arterial pressure was decreased at 0 and 15 min post-RE60 and at 15 post-RE75 without changes after C60 and C75. The decrease in mean arterial pressure was lower in RE60 and RE75 compared to C60 and C75. There were no changes in the state of anxiety Y1. We conclude that higher intensity aerobic exercise has the greatest effect on lowering systolic blood pressure, while low intensity resistance exercise is more effective in reducing diastolic blood pressure and mean arterial pressure.

**Keywords:** systolic blood pressure; diastolic blood pressure; mean arterial pressure; aerobic training; resistance exercise.

**Funding:** This research received no external funding.

### 2.2. No Impact of Three Whole-Body Vibration Protocols on Isometric Knee Extension Strength and Vastus Lateralis EMG Activity in High-Level Adolescent Soccer Players

Panagiotis Ampeliotis ^1,^*, Spyridon Methenitis ^2^, Apostolos Z. Skouras ^1^, Dimitrios Antonakis-Karamintzas ^1^, Georgios Papagiannis ^1,3^, Charilaos Tsolakis ^1,2^ and Panagiotis Koulouvaris ^1^

^1^ 1st Department of Orthopaedic Surgery, School of Medicine, National and Kapodistrian University of Athens, 12462 Athens, Greece; pan.ampeliotis@gmail.com; apostolis.sk@gmail.com; jkaramintzas71@gmail.com; grpapagiannis@yahoo.gr; tsolakis@phed.uoa.gr; info@drkoulouvaris.gr

^2^ Sports Performance Laboratory, School of Physical Education & Sports Science, National and Kapodistrian University of Athens, 17237 Athens, Greece; smetheni@phed.uoa.gr

^3^ Biomechanics Laboratory, Department of Physiotherapy, University of the Peloponnese, 23100 Sparta, Greece

* Correspondence: pan.ampeliotis@gmail.com

**Abstract:** Acute whole-body vibration (WBV) has been shown to influence neuromuscular responses in adult soccer players, particularly those at high professional levels (1). While there is evidence to suggest that acute WBV can enhance isometric strength and electromyographic (EMG) activity in the quadriceps muscle in adults, the optimal protocol for its application in adolescents remains to be fully explored (2–4). This study investigates the acute effects of three different WBV protocols on isometric knee extension strength and EMG activity of vastus lateralis muscle. Twelve adolescent male soccer players (aged 16.21 ± 0.32 years; height 176.94 ± 8.34 cm; weight 67.29 ± 6.50 kg) from a U-16 professional team participated. Players underwent WBV on a Galileo 900 vibration device using three protocols: 1 set of 3 min (P1), 3 sets of 1 min (P2), and 6 sets of 30 s (P3). All were administered at a semi-squat position with a frequency of 25 Hz. The protocols were conducted on separate days in a random sequence, following a rest day. Isometric knee extension at 90° of the dominant limb was assessed using an isokinetic dynamometer while simultaneously recording integrated EMG of the vastus lateralis muscle. Parameters such as isometric peak force, time to peak force production, rate of force development (RFD), and EMG variables were measured before, and 2-, 5-, and 15-min post-intervention. The results showed no statistically significant differences between protocols and time points (*p* > 0.05). The most substantial percentage reduction in knee extension peak force was observed in P1 at times 2 min (−15.22%), 5 min (−15.28%), and 15 min (−14.11%) post-WBV. Time to peak force production was most affected in P1 (pre: 1.42 ± 1.08 s, 2 min: 2.81 ± 1.73 s, 5 min: 2.38 ± 1.39 s, 15 min: 2.94 ± 1.66 s; *p* = 0.056). Although not statistically significant, increases of over 10% in RFD at 80 ms were noted 5 min post-intervention for both P1 and P3, but a decrease was observed in RFD at 150 and 250 ms. The physiological activity of the vastus lateralis was consistent across all protocols and time points (root-mean-square across time points for P1 *p* = 0.901; P2 *p* = 0.922; and P3 *p* = 0.865). In conclusion, different WBV protocols had no discernible effect on isometric knee extension strength and EMG activity in high-level adolescent soccer players. Even though no protocol achieved statistical significance, the prolonged P1 protocol exhibited the most notable strength and physiological impact.

**Keywords:** post-activation potentiation; mechanical oscillation; fatigue; elite soccer.

**Funding:** This research received no external funding.

**References**

1. Cloak, R.; Lane, A.; Wyon, M. Professional soccer player neuromuscular responses and perceptions to acute whole body vibration differ from amateur counterparts. *J Sports Sci Med* **2016**, *15*, 57–64.
2. Wang, Z.; Wei, Z.; Li, X.; Lai, Z.; Wang, L. Effect of whole-body vibration on neuromuscular activation and explosive power of lower limb: A systematic review and meta-analysis. *PLoS One* **2022**, *17*, e0278637. doi:10.1371/journal.pone.0278637.
3. Jordan, M.; Norris, S.; Smith, D.; Herzog, W. Acute effects of whole-body vibration on peak isometric torque, muscle twitch torque and voluntary muscle activation of the knee extensors. *Scand J Med Sci Sports* **2010**, *20*, 535–540. doi:10.1111/j.1600-0838.2009.00973.x.
4. Marín, P.J.; Cochrane, D.J. The effects of whole-body vibration on EMG activity of the lower body muscles in supine static bridge position. *J Musculoskelet Neuronal Interact* **2021**, *21*, 59–67.

### 2.3. The Effect of a Combination of Strength Training Programme on the Development of Vertical and Standing Long Jump of Female Young Basketball Players

Panagiotis Androutsopoulos ^1,^*, Konstantinos Ntouvas ^1^ and Ilias Blantas ^2^

^1^ Hellenic Basketball Federation 37 Kifissias Avenue, Marousi, Greece; panagiotisandrouts@gmail.com

^2^ MSc Graduate of the Department of Physical Education & Sport Science, Aristotle University of Thessaloniki, Greece; iliasbladas@gmail.com

* Correspondence: panagiotisandrouts@gmail.com

**Abstract:** Basketball is a sport that requires outbursts of strength and power that derives of both the extremities and the core of the body (1). In recent years, basketball games have become more fast paced with increased physiological demands that require the acquisition of specialized technical skills and the development of certain physical abilities from a young age, especially in teenaged girls. The purpose of the study was to examine the effect of applying a combination program of lower extremity plyometric exercises (jumps with a progressively increasing degree of difficulty) and limb-trunk stabilization strength exercises, on the improvement of the vertical jump (SJ, CMJ, DJ) and horizontal jump (SLJ) in female basketball athletes aged 13–14 years. The hypothesis was that the intervention group would greatly improve its jumping ability in both jumping conditions after the implementation of the strength protocol. The sample size consisted of 65 female athletes (13–14 years old) from various basketball academies, who were randomly divided into 2 groups (experimental n = 43 and control n = 22 female athletes). In order to acquire the results of the jumping measurements, an optical data collection system (Optojump) was used, which measures the contact and flight times in a series of jumps with an accuracy of milliseconds (1/1000 s). The intervention group participated in a strength program consisting of plyometric exercises (jumps of progressively increasing difficulty and intensity), lasting 12 weeks, with practices occurring 2 times/week, along with the standardized basketball technique training program (4 times/week). The control group only followed the standardized basketball training program (4 times/week). Measurements were taken before and at the end of the intervention program (12 weeks), as well as 2 months after its completion, in order to investigate if there was strength maintenance in both groups. All of the measurements were made in a closed basketball court so the process would not be affected by the weather conditions. Statistical analysis of variance with repeated measures (ANOVA repeated measures) showed statistically significant differences between the groups. The female athletes of the experimental group were significantly better in SJ, CMJ and SLJ compared to the control group, while mixed results between the groups were found in the DJ measurements. The findings confirm the existing literature on the positive use of combined strength training protocols, specifically trunk-extremity strength-stabilization exercises with plyometric and suggests that coaches use combined strength training for the development of the jumping abilities in developmental age female basketball athletes.

**Keywords:** plyometric training; teenage girls; strength training; basketball.

**Funding:** This research received no external funding.

**References**

1. Kotzamanidis, C. Effect of plyometric training on running performance and vertical jumping in prepubertal boys. *J Strength Cond Res* **2006**, *20*, 441–445. doi:10.1519/R-16194.1

### 2.4. Different Types of Concurrent Dry-Land Resistance and Sprint Swimming Training on Swimming Performance

Gavriil Arsoniadis ^1,^*, Ioannis Chalkiadakis ^1^, Petros Botonis ^1,2^, Gerasimos Terzis ^3^, Gregory Bogdanis ^3^ and Argyris Toubekis ^1,3^

^1^ Division of Aquatic Sports, School of Physical Education and Sports Science, National and Kapodistrian University of Athens, 17237 Dafne, Greece; garsoniadis@phed.uoa.gr; giannischalk@phed.uoa.gr; pboton@phed.uoa.gr; atoubekis@phed.uoa.gr

^2^ Division of Sports Medicine and Biology of Exercise, School of Physical Education and Sports Science, National and Kapodistrian University of Athens, 17237 Dafne, Greece

^3^ Sports Performance Laboratory, School of Physical Education and Sports Science, National and Kapodistrian University of Athens, 17237 Dafne, Greece; gterzis@phed.uoa.gr; gbogdanis@phed.uoa.gr

* Correspondence: garsoniadis@phed.uoa.gr; Tel.: +306983599847

**Abstract:** Swimmers apply concurrent dry-land and swimming training (SWT) with different training types (1–4). Aim of the study was to examine the effect of concurrent dry-land and sprint SWT or SWT only on swimmers’ performance, physiological and biomechanical variables following six weeks of training. Twenty-four swimmers (N = 24, age: 16.5 ± 2.9 years, 12 males and 12 females) were assigned to three groups of equal performance level, (i) maximum strength group (G-MS) performed 3 sets × 4 repetitions, load 90% of one repetition maximum (1RM), 3 min rest between sets, prior to SWT, (ii) strength endurance group (G-SE) performed 2 sets × 20 repetitions, load 55% of 1RM, 20 s rest between sets prior to SWT, (iii) control group (G-CON) performed only SWT (1000-m warm-up, 4 × 50-m front crawl and 4 × 50-m in the preferred stroke starting every 2 min). These sessions were applied 3 times per week for 6-weeks. Swimmers were evaluated in 1RM before (pre) and after (post) the 6-week training period in bench press, seated pull rowing, half-squat, critical speed in front crawl swimming using 200 and 400-m distances. Moreover, performance time (T), arm-stroke rate (SR), arm-stroke length (SL), arm-stroke index (SI) were measured during 50, 100, 200, 400, and 4 × 50-m front crawl tests. Additionally, force was measured during tethered swimming (TF) and shoulder isometric strength (ISO), and hand grip strength (HG) were evaluated. Blood lactate concentration [La^−^] and heart rate (HR) were measured before and after 400-m and 4 × 50-m test. Pre vs. post 1RM was increased in bench press for all groups, while seated-pull rowing and half squat only for G-MS and G-SE (*p* = 0.01). Critical speed improved in all groups (*p* = 0.01). Pre vs. post T improved in all groups (G-MS, 50-m: 4.9 ± 2.4%; 100-m: 5.0 ± 2.2%; 200-m: 4.8 ± 3.9%; 400-m: 3.4 ± 2.3%, 4 × 50-m: 3.1 ± 4.6%; G-SE, 50-m: 4.7 ± 2.9%; 100-m: 4.4 ± 2.2%; 200-m: 6.0 ± 3.5%; 400-m: 4.2 ± 3.3%, 4 × 50-m: 4.0 ± 2.3%; G-CON, 50-m: 2.6 ± 2.7%; 100-m: 2.1 ± 3.2%; 200-m: 3.3 ± 1.5%; 400-m: 3.7 ± 3.3%, 4 × 50-m: 1.2 ± 4.8%, *p* = 0.01). Post, SR was increased in 50, 100, and 400-m in all groups (*p* = 0.01), while SL only in 200-m (*p* = 0.01), and SI in 400-m (*p* = 0.01). TF and ISO increased in all groups (*p* = 0.01) while HG increased to a higher extent in G-MS (14.9 ± 8.4%) and G-SE (13.0 ± 9.4%) compared to G-CON (5.0 ± 20.0%; *p* = 0.01). [La^−^] increased after 400-m in all groups (*p* = 0.01). Post HR increased only in G-SE during 4 × 50-m (*p* = 0.01). Both types of dry-land resistance training and swimming training only are equally effective in performance improvement following a 6-week training period.

**Keywords:** sprint swimming training; dry-land resistance training; concurrent training.

**Funding:** This research received no external funding.

**References**

1. Amaro, N.M.; Marinho, D.A.; Marques, M.C.; Batalha, N.P.; Morouço, P.G. Effects of Dry-Land Strength and Conditioning Programs in Age Group Swimmers. *J Strength Cond Res.* **2017**, *31*, 2447–2454.

2. Aspenes, S.; Kjendlie, P.L.; Hoff, J.; Helgerud, J. Combined strength and endurance training in competitive swimmers. *J Sports Sci Med* **2009**, *8*, 357–365.

3. Arsoniadis, G.; Botonis, P.; Bogdanis, G.C.; Terzis, G.; Toubekis, A. Acute and Long-Term Effects of Concurrent Resistance and Swimming Training on Swimming Performance. *Sports* **2022**, *10*, 29.

4. Crowley, E.; Harrison, A.J.; Lyons, M. The Impact of Resistance Training on Swimming Performance: A Systematic Review. *Sports Med* **2017**, *47*, 2285–2307.

### 2.5. Effects of 30 km Military Endurance March on Neuromuscular System in Spanish Army Marines

Beltrán Cáceres ^1,^*, Cristian Marín ^2^, Pablo Martínez de Baños ^3^ and Pedro E. Alcaraz ^1,2^

^1^ Faculty of Sport Sciences, Catholic University of Murcia, UCAM, 30107 Murcia, Spain; beltran_2301@hotmail.com; palcaraz@ucam.edu

^2^ UCAM Research Center for High Performance Sports, Catholic University of Murcia, UCAM, 30107 Murcia, Spain; cmarin@ucam.edu

^3^ Department of Instruction and Training, Marine Infantry School “General Albacete y Fuster”, 30201 Cartagena, Spain; pmarma9@mde.es

* Correspondence: beltran_2301@hotmail.com

**Abstract:** Infantry soldiers must cover long distances carrying heavy and bulky combat equipment [1]. At times, the success of the mission or survival depends on reaching one point from another as quickly as possible, despite multiple stressful factors that hinder their work [2,3]. Since the beginning of their training, Marines have undergone this characteristic and demanding test. However, little is known about how it affects the neuromuscular level and recovery in the days following the test. Twenty-six Marines completed the test, of which three experienced severe discomfort, resulting in a final sample of twenty-three Marines (age, 28.3 ± 4.98 years; height, 176 ± 5.79 cm; weight, 80.0 ± 7.79 kg) who underwent pre, post, post-24 h, and post-48 h evaluations after the 30 km endurance march carrying their 34 kg combat equipment. Grip strength (HG), isometric mid-thigh pull (IMTP), countermovement jump height (Hmax CMJ), blood lactate (La), body weight (BW), rating of fatigue scale (ROF), maximum pull-ups in two minutes (PUmax), and incident reports (INRE) during the test were assessed. Repeated measures ANOVA, paired samples *t*-test, and effect size (ES) analysis were conducted, presenting the results as mean ± SD. The variable, *p*-value of changes over time, means ± SD and the percentage decrease or increase [pre-post, pre-post-24 h, pre-post-48 h] are presented. ROF (*p* ≤ 0.001) 1.96 ± 0.56, 7.57 ± 0.66, 4.96 ± 1.19, 3.43 ± 1.12 [+74.11% (*p* ≤ 0.001), +60.48% (*p* ≤ 0.001), +42.86% (*p* ≤ 0.001)]; BW (*p* ≤ 0.001) 80.00 ± 7.79, 77.30 ± 7.88, 78.4 ± 7.81, 78.5 ± 8.03 [−3.49% (*p* ≤ 0.001), −2.04% (*p* ≤ 0.001), −1.91% (*p* ≤ 0.001)]; La (*p* = 0.266) 2.36 ± 0.79, 3.05 ± 2.84 [+22.62% (*p* = 0.266)]; PUmax (*p* ≤ 0.001) 17.70 ± 3.23, 13.90 ± 3.48, 15.7 ± 2.72, 16.7 ± 3.19 [−21.47% (*p* ≤ 0.001), −11.30% (*p* ≤ 0.001), −5.65% (*p* ≤ 0.001)]; HG left (*p* = 0.344) 47.00 ± 6.88, 49.1 ± 7.08, 47.7 ± 6.48, 47.9 ± 6.54 [+4.28% (*p* = 0.149), +1.47% (*p* = 0.456), +1.88% (*p* = 0.487)]; HG right (*p* = 0.375) 49.90 ± 6.97, 50.90 ± 7.18, 51.2 ± 7.19, 50.3 ± 6.75 [+1.97% (*p* = 0.224), +2.54% (*p* = 0.145), +0.80% (*p* = 0.641)]; IMTP (*p* = 0.004) 2612 ± 291, 2637 ± 293, 2463 ± 301, 2486 ± 248 [+0.98% (*p* = 0.682), −5.70% (*p* ≤ 0.01), −4.82% (*p* ≤ 0.001)]; Hmax CMJ 32.2 ± 5.96, 27.8 ± 6.23, 28.5 ± 4.81, 27.7 ± 4.50 (*p* ≤ 0.001) [−13.67% (*p* ≤ 0.001), −11.49% (*p* ≤ 0.001), −13.98% (*p* ≤ 0.001)]. INRE showed a progressive and significant increase over time (*p* value) and between first 10 km and last 5 km [%] test in perceived variables of fatigue (*p* ≤ 0.001) 30.0 ± 22.2 and 54.8 ± 29.5 [+45.3% (*p* ≤ 0.001)], muscle pain (*p* ≤ 0.001) 37.8 ± 24.3 and 70.4 ± 19.4 [+46.3% (*p* ≤ 0.001)], joint pain (*p* ≤ 0.001) 33.9 ± 26.1 and 60.0 ± 28.6 [+43.5% (*p* ≤ 0.001)], shortness of breath (*p* ≤ 0.001) 24.8 ± 14.7 and 35.2 ± 23.3 [+29.6% (*p* ≤ 0.01)], excessive sweating (*p* ≤ 0.001) 27.4 ± 17.9 and 48.3 ± 25.2 [+43.3% (*p* ≤ 0.001)], and muscle tremors (*p* = 0.028) 24.8 ± 14.7 and 30.0 ± 18.1 [+17.3% (*p* = 0.103)], except for palpitations (*p* = 0.189) 24.8 ± 14.7 and 30.0 ± 20.2 [+17.3% (*p* = 0.162)]. In conclusion, the subjective perception of fatigue in the military personnel showed optimal recovery after the test; nevertheless, the results obtained indicate that this one had a significant impact on the neuromuscular level, with no recovery observed in overall strength and lower limb power after 48 h. The resilient spirit of operational military units and their philosophy of being always ready for combat could increase the injury rate.

**Keywords:** military; load carriage; neuromuscular fatigue; performance; physical readiness and conditioning.

**Funding:** This research received no external funding.

**References**

1. Nindl, B.C.; Alvar, B.A.; R. Dudley, J.; Favre, M.W.; Martin, G.J.; Sharp, M.A.; et al. Executive Summary From the National Strength and Conditioning Association’s Second Blue Ribbon Panel on Military Physical Readiness: Military Physical Performance Testing. *J Strength Cond Res* **2015**, *29* (Suppl. 11), S216–220.
2. Kyröläinen, H.; Pihlainen, K.; Vaara, J.P.; Ojanen, T.; Santtila, M. Optimising training adaptations and performance in military environment. *J Sci Med Sport* **2018**, *21*, 1131–8.
3. Boffey, D.; Harat, I.; Gepner, Y.; Frosti, C.L.; Funk, S.; Hoffman, J.R. The Physiology and Biomechanics of Load Carriage Performance. *Mil Med* **2019**, *184*, e83–90.

### 2.6. Neuromuscular Responses to 5 km Time Trial Load Carried in Spanish Army Marines

Beltrán Cáceres ^1,^*, Cristian Marín ^2^, Pablo Martínez de Baños ^3^ and Pedro E. Alcaraz ^1,2^

^1^ Faculty of Sport Sciences, Catholic University of Murcia, UCAM, 30107 Murcia, Spain; beltran_2301@hotmail.com; palcaraz@ucam.edu

^2^ UCAM Research Center for High Performance Sports, Catholic University of Murcia, UCAM, 30107 Murcia, Spain; cmarin@ucam.edu

^3^ Department of Instruction and Training, Marine Infantry School “General Albacete y Fuster”, 30201 Cartagena, Spain; pmarma9@mde.es

* Correspondence: beltran_2301@hotmail.com

**Abstract:** One of the requirements for Marines is to cover a specified distance while carrying their individual combat gear, supplies, or other military equipment through challenging terrain [1,2]. The serviceman must be capable of moving from one point to another in the shortest time possible, both in offensive actions to reach their objective and in defensive actions where the operational team must reach a secure extraction point. Training for this distinctive physically and psychologically demanding test [3,4] is part of the routine for Marines. Nevertheless, further studies are necessary to determine the neuromuscular impact of such demanding tests, as well as their recovery in the days following. Twenty-nine Marines (age, 28.7 ± 4.74 years; height, 176 ± 5.39 cm; weight, 78.4 ± 7.99 kg) performed the 5 km time trial carrying their 24 kg combat gear. They were evaluated pre, post, post-24 h, and post-48 h for grip strength (HG), isometric mid-thigh pull (IMTP) performance, countermovement jump height (Hmax CMJ), blood lactate (La) levels, body weight (BW), rating of fatigue scale (ROF), maximum pull-ups in two minutes (PUmax), and incident reports (INRE) during the test. Repeated measures ANOVA, paired samples t-test, and effect size (ES) analysis were conducted, presenting the results as mean ± SD. The variable, the *p*-value of changes over time, means ± SD and the percentage decrease or increase [pre-post, pre-post-24 h, pre-post-48 h] are presented. ROF (*p* ≤ 0.001) 2.34 ± 0.81, 8.95 ± 0.76, 4.12 ± 1.02, 3.59 ± 0.63 [+73.86% (*p* ≤ 0.001), +43.20% (*p* ≤ 0.001), +34.82% (*p* ≤ 0.001)], BW (*p* ≤ 0.001) 78.4 ± 7.99, 78.0 ± 7.94, 78.0 ± 8.06, 78.4 ± 8.11 [−0.51% (*p* ≤ 0.001), −0.51% (*p* ≤ 0.001), 0% (*p* = 0.915)], La (*p* ≤ 0.001) 2.20 ± 0.00, 10.53 ± 0.73 [+79.11% (*p* ≤ 0.001)], PUmax (*p* ≤ 0.001) 17.3 ± 4.73, 15.3 ± 4.41, 15.0 ± 3.66, 15.7 ± 3.67 [−11.56% (*p* ≤ 0.001), −13.30% (*p* ≤ 0.001), −9.25% (*p* ≤ 0.001)], HG left (*p* = 0.918) 49.2 ± 6.67, 49.2 ± 7.52, 48.8 ± 6.76, 49.4 ± 6.81 [0% (*p* = 0.981), −0.81% (*p* = 0.626), +0.41% (*p* = 0.803)], HG right (*p* = 0.179) 52.0 ± 7.55, 50.1 ± 8.03, 51.2 ± 7.06, 51.3 ± 6.59 [−3.65% (*p* = 0.047), −1.54% (*p* = 0.273), −1.35% (*p* = 0.0446)], IMTP (*p* ≤ 0.001) 2590 ± 301, 2633 ± 340, 2427 ± 301, 2472 ± 312 [+1.63% (*p* = 0.197), −6.29% (*p* ≤ 0.001), −4.56% (*p* ≤ 0.01)], Hmax CMJ (*p* ≤ 0.001) 31.3 ± 5.00, 32.1 ± 5.4, 30.3 ± 5.12, 30.3 ± 4.97 [+2.49% (*p* = 0.137), −3.20% (*p* ≤ 0.05), −3.20% (*p* ≤ 0.05)]. INRE showed a progressive and significant (*p* ≤ 0.001) increase over time in all perceived variables and between first 10 km and last 5 km [%] test of fatigue 14.5 ± 15.3 and 75.5 ± 28.5 [+80.8% (*p* ≤ 0.001)], muscle pain 13.4 ± 15.2 and 45.5 ± 37.3 [+70.6% (*p* ≤ 0.001)], joint pain 11.4 ± 14.8 and 27.9 ± 38.4 [+59.1% (*p* ≤ 0.01)], shortness of breath 12.4 ± 15.0 and 60.0 ± 34.9 [+79.3% (*p* ≤ 0.001)], excessive sweating 13.4 ± 17.2 and 73.4 ± 23.5 [+81.7% (*p* ≤ 0.001)], muscle tremors 11.4 ± 14.8 and 23.8 ± 32.4 [+52.1% (*p* ≤ 0.01)], and perceived palpitations 10.3 ± 14.5 and 29.0 ± 37.2 [+64.5% (*p* ≤ 0.001)]. In conclusion, the military test had a significant neuromuscular impact on the body, resulting in a potentiation of absolute global isometric strength and lower limb power immediately after the test, with values remaining below baseline at 24 and 48 h.

**Keywords:** military; soldier; military personnel; load carriage; neuromuscular fatigue; performance; physical readiness and conditioning.

**Funding:** This research received no external funding.

**References**

1. Knapik, J.J.; Reynolds, K.L.; Harman, E. Soldier load carriage: historical, physiological, biomechanical, and medical aspects. *Mil Med* **2004**, *169*, 45–56.
2. Vaara, J.P.; Groeller, H.; Drain, J.; Kyröläinen, H.; Pihlainen, K.; Ojanen, T.; et al. Physical training considerations for optimizing performance in essential military tasks. *Eur J Sport Sci* **2022**, *22*, 43–57.
3. Faghy, M.A.; Shei, R.J.; Armstrong, N.C.D.; White, M.; Lomax, M. Physiological impact of load carriage exercise: Current understanding and future research directions. *Physiol Rep* **2022**, *10*, e15502.
4. Tornero-Aguilera, J.F.; Robles-Pérez, J.J.; Clemente-Suárez, V.J. Effect of Combat Stress in the Psychophysiological Response of Elite and Non-Elite Soldiers. *J Med Syst* **2017**, *41*, 100.

### 2.7. Glycated Hemoglobin Improvements by Diet and Physical Exercise Are Not Linked to Interleukin-6 Changes in Type 2 Diabetes

Cristina Casals ^1,^*, Manuel Costilla ^1^, María Rebollo-Ramos ^1^, Adrián Montes-de-Oca-García ^1^, Alba Mier-Perulero ^1^, Rafa Baturone-Servan ^1^, Alejandro Perez-Bey ^2^, Jesus G. Ponce-Gonzalez ^1^ and Juan Corral-Pérez ^1^

^1^ ExPhy Research Group, University of Cadiz, Spain; cristina.casals@uca.es; manueljesus.costilla@uca.es; maria.rebollo@uca.es; adrian.montesdeoca@uca.es; alba.mierperulero@alum.uca.es; rafa.baturoneservan@alum.uca.es; jesusgustavo.ponce@uca.es; juan.corral@uca.es

^2^ GALENO Research Group, University of Cadiz, Spain; alejandro.perezperez@uca.es

* Correspondence: cristina.casals@uca.es; Tel.: +34-677-18-05-97

**Abstract:** Molecules originating from adipose tissue regulate various physiological mechanisms, including energy metabolism, insulin sensitivity, and the inflammatory response (1). Indeed, a higher degree of obesity seems to be associated with a chronic increase in interleukin-6 (IL-6), and this elevation may be even more pronounced in individuals with type 2 diabetes (T2D) and obesity (2). However, the impact of exercise and diet on IL-6 levels and their potential relationship with the reduction of adipose tissue and glycated hemoglobin in adults with T2D is not well-understood. Therefore, the aim of this study was to examine the relationship between changes in IL-6, body fat mass, and glycated hemoglobin induced by a 12-week intervention involving physical exercise, diet modification, or both in adults with T2D. In fasting conditions, blood samples were collected for the immediate assessment of IL-6 and glycated hemoglobin levels, and body composition was evaluated according to international protocols using an 8-electrode bioimpedance method (TANITA) in 81 adults with T2D and overweight/obesity. Participants were randomly assigned to one of three groups: high-intensity intermittent training (HIIT), moderate-intensity continuous training (MICT), or the inactive group. Within these three groups, participants were further randomized into either the dietary group or the control group. The training groups conducted three physical exercise sessions per week, and the dietary groups adhered to weekly menus featuring a Mediterranean diet with a targeted reduction of approximately 300 calories with sessions each two weeks, during 12 weeks. According to the factorial ANOVA, glycated hemoglobin was significantly reduced (*p* < 0.05) by the diet intervention (for the HIIT, MICT, or inactive groups), but there were no significant changes in IL-6 levels. Similarly, the bivariate correlations of the pre-post differences (Δ) revealed no significant relationships between Δ IL-6, Δ glycated hemoglobin, or Δ body fat mass. Hence, it is possible that IL-6 originating from adipose tissue may not be a main factor contributing to impaired T2D control. In fact, substantial increases in IL-6 induced by acute physical exercise are known to diminish relatively quickly, even in individuals who are obese and have T2D (3). Consequently, there is an ongoing scientific debate regarding the pro-inflammatory or anti-inflammatory role of IL-6 and other cytokines (4). Finally, it’s important to emphasize that, despite the fact that only the dietary groups showed significant improvements in T2D control based on glycated hemoglobin levels, physical exercise enhances physical fitness and overall health, although other outcomes were not included in this study.

**Keywords:** inflammatory markers; obesity; Mediterranean diet; body fat; body composition.

**Funding:** This research was funded by Supreme Council for Sports (CSD) of Spanish Ministry through the Recovery Plan, Transformation and Resilience, grant number EXP_74977.

**References**

1. Dludla, P.V.; Nkambule, B.B.; Mazibuko-Mbeje, S.E.; Nyambuya, T.M.; Mxinwa, V.; Mokgalaboni, K; Ziqubu, K.; Cirilli, I.; Marcheggiani, F.; Louw, J.; Tiano, L. Adipokines as a therapeutic target by metformin to improve metabolic function: A systematic review of randomized controlled trials. *Pharmacol Res* **2021**, *163*, 105219.
2. Rodrigues, K.F.; Pietrani, N.T.; Bosco, A.A.; Campos, F.M.F.; Sandrim, V.C.; Gomes, K.B. IL-6, TNF-α, and IL-10 levels/polymorphisms and their association with type 2 diabetes mellitus and obesity in Brazilian individuals. *Arch Endocrinol Metab* **2017**, *61*, 438–446.
3. Robbins, J.M., Gerszten, R.E. Exercise, exerkines, and cardiometabolic health: from individual players to a team sport. *J Clin Invest* **2023**, *133*, e168121.
4. Bobbo, V.C.D.; Jara, C.P.; Mendes, N.F.; Morari, J.; Velloso, L.A.; Araújo, E.P. Interleukin-6 Expression by Hypothalamic Microglia in Multiple Inflammatory Contexts: A Systematic Review. *Biomed Res Int* **2019**, *2019*, 1365210.

### 2.8. Mechanical Deviations in Running Kinematics during Fatigue Accumulation: An Association with Physiological Indices of Muscle Exhaustion

Christos Chalitsios ^1,2,^*, Thomas Nikodelis ^1^ and Vassilis Mougios ^3^

^1^ AUTh Biomechanics Laboratory; School of Physical Education and Sport Science at Thessaloniki, Aristotle University of Thessaloniki, Greece; cchalits@phed.auth.gr

^2^ AUTh Biomechanics Laboratory; School of Physical Education and Sport Science at Thessaloniki, Aristotle University of Thessaloniki, Greece; cchalits@phed.auth.gr

^3^ Laboratory of Evaluation of Human Biological Performance, School of Physical Education and Sport Science at Thessaloniki, Aristotle University of Thessaloniki, Greece; mougios@auth.gr

* Correspondence: cchalits@phed.auth.gr; Tel.: +30-2310-992202

**Abstract:** Biomechanical studies often overlook physiological metrics, such as muscle effort, thereby missing out on insights into how muscle physiology impacts movement, especially during strenuous exercise. A key metric is muscle oxygen saturation (SmO_2_), which measures the percentage of oxygen-bound heme molecules in muscle tissue. SmO_2_ is assessed using wearable devices employing near-infrared spectroscopy (NIRS). The aims of this study were to (a) examine changes in the mechanical characteristics of running as fatigue accumulates, using time series data of lower extremity kinematics, and (b) associate these changes with SmO_2_ as an index of muscle fatigue. We hypothesized that deviations from typical running patterns would increase as runners approached fatigue and that changes in SmO_2_ would account for a significant portion of this variance. Sixteen youth competitive runners participated in the study. Biomechanical and physiological data were collected in an outdoor track during an 8-min running test near exhaustion. Inertial measurement units were placed on the right tibia and lower foot, and a NIRS sensor was affixed to the rectus femoris. Data from the initial 40% of the trial were used to establish each runner’s typical movement pattern via a one-class support vector machine model. The percentage of outlier strides, based on the remaining data segmented into 15% test sets, defined the overall change in each individual’s movement pattern. The model revealed a significant increase in outliers (*p* < 0.001) in the last 15% of the trial. Linear regression showed that the average SmO_2_ values during the same time interval accounted for 40% of the variation in the percentage of outlier (R^2^ = 0.40, *p* = 0.009). These findings offer a proof-of-concept and a novel methodological approach for integrating machine learning and wearable sensor data to objectively monitor deviations in human movement patterns in response to fatigue during high-intensity exercise.

**Keywords:** running patterns; fatigue; muscle oxygen saturation; one-class support vector machine; outliers.

**Funding:** This research received no external funding.

**References**

1. Perrey, S.; Ferrari, M. Muscle Oximetry in Sports Science: A Systematic Review. *Sports Med* **2018**, *48*, 597–616.
2. Kirby, B.S.; Clark, D.A.; Bradley, E.M.; Wilkins, B.W. The balance of muscle oxygen supply and demand reveals critical metabolic rate and predicts time to exhaustion. *J Appl Physiol* **2021***, 130*, *1915–1927*.
3. Schölkopf, B.; Platt, J.C.; Shawe-Taylor, J.; Williamson, R.C. Estimating the support of a high-dimensional distribution. *Neural Comput* **2001**, *13*, 1443–1471.
4. Kobsar, D.; Ferber, R. Wearable Sensor Data to Track Subject-Specific Movement Patterns Related to Clinical Outcomes Using a Machine Learning Approach. *Sensors* **2018**, *18*, 2828.

### 2.9. Long COVID-19 Syndrome: Does Gender Affect the Outcomes of a Rehabilitation Programme?

Nikolaos Chynkiamis ^1,2,^*, Angelos Vontetsianos ^1^, Christina Anagnostopoulou ^1^, Christiana Lekka ^1^, Christos F. Kampolis ^3^, Ioannis Vogiatzis ^1,4^ and Nikolaos Koulouris ^1^

^1^ Rehabilitation Unit, 1st Respiratory Medicine Department, “Sotiria” Hospital, National and Kapodistrian University of Athens, Greece; nikchy@thorax.org.gr; agelvonte@gmail.co; christinaanagnosto@gmail.com; christiana.lekka@gmail.com; ioannis.vogiatzis@northumbria.ac.uk; koulnik@med.uoa.gr

^2^ Thorax Research Foundation, Athens, Greece

^3^ Department of Emergency Medicine, “Hippokration” General Hospital of Athens, Athens, Greece; chkamp77@gmail.com

^4^ Department of Sport, Exercise and Rehabilitation, Faculty of Health and Life Sciences, Northumbria University Newcastle, Newcastle upon Tyne, United Kingdom

* Correspondence: nikchy@thorax.org.gr

**Abstract:** Female sex is associated with higher prevalence of long COVID-19 syndrome following hospital admission six months after discharge. Patients with long COVID-19 syndrome exhibit improvement in exercise tolerance, quality of life and functional capacity following the implementation of pulmonary rehabilitation programmes. The benefits of rehabilitation on the two genders remain inconclusive. Accordingly, the aim of the study was to compare the effect of a hybrid Pulmonary Rehabilitation (PR) program on functional capacity, quality of life and exercise tolerance in men and women with long COVID-19 syndrome. Twenty-seven patients (15 men mean ± SD (53 ± 16 years) and 12 women (54 ± 9 years)) completed PR consisting of 8 outpatient PR sessions (twice weekly for 4 weeks) and 24 home-based sessions (3 times/week for 8 weeks), 5 ± 2 months post-hospital discharge. QoL (fatigue, symptoms, anxiety, depression and post-traumatic stress disorder), functional capacity (six-minute walk test, peripheral muscle strength and daily physical activity) and maximum inspiratory (PImax) were assessed prior and following the completion of the programme. Exercise tolerance was assessed via an incremental ramp cardiopulmonary exercise test. Following the completion of a hybrid PR programme, the magnitude of improvement in PImax as a fraction of predicted normal was identical between men and women (from 91 ± 24% to 101 ± 27% and from 34 ± 17 to 44 ± 15% (*p* = 0.452), respectively). Fatigue, assessed via Functional Assessment of Chronic Illness Therapy (FACIT) questionnaire, was similarly improved in men and women (from 30 ± 8 to 44 ± 8 and from 23 ± 11 to 40 ± 8, (*p* = 0.570), respectively). The improvement in the COPD Assessment Tool score was comparable between men and women (from 13 ± 8 to 7 ± 5 and from 15 ± 9 to 7 ± 5 (*p* = 0.449), respectively). Only women exhibited clinically meaningful improvement in HADS anxiety (from 7 ± 5 to 3 ± 4, *p* = 0.001) and depression (from 7 ± 4 to 4 ± 4, *p* = 0.006) scores. Six-minute walk distance was improved in both men and women (from 418 ± 91 metres to 506 ± 84 metres and from 385 ± 94 to 437 ± 121 metres, (*p* = 0.143), respectively). Exercise tolerance was similarly improved in men and women (from 110 ± 47 Watts to 132 ± 50 Watts and from 73 ± 37 Watts to 89 ± 42 Watts (*p* = 0.231), respectively). Finally, VO_2_peak was similarly improved in men and women (from 17.5 ± 6.0 mL/kg/min to 20.4 ± 6.3 mL/kg/min and from 15.2 ± 17.0 mL/kg/min to 17.0 ± 7.5 mL/kg/min, (*p* = 0.198), respectively). In conclusion, the application of a hybrid PR in patients with long COVID-19 is equally effective in both men and women, particularly for those with high anxiety and depression.

**Keywords:** long COVID-19 syndrome; quality of life; functional capacity; exercise tolerance.

**Funding:** This research received no external funding.

**References**

1. Goërtz, Y.M.J.; Van Herck, M.; et al. Persistent symptoms 3 months after a SARSCoV-2 infection: the post-COVID-19 syndrome? *ERJ Open Res* **2020**, *6*, doi:10.1183/23120541.00542-2020.
2. Carfì, A.; Bernabei, R.; et al. Persistent Symptoms in Patients After Acute COVID-19. *JAMA* **2020**, *324*, 603–605, doi:10.1001/jama.2020.12603.
3. Evans, R.A.; McAuley, H.; et al. Physical, cognitive, and mental health impacts of COVID-19 after hospitalisation (PHOSP-COVID): a UK multicentre, prospective cohort study. *Lancet Respir Med* **2021**, *9*, 1275–1287, doi:10.1016/s2213-2600(21)00383-0.
4. Evans, R.A.; Leavy, O.C.; et al. Clinical characteristics with inflammation profiling of long COVID and association with 1-year recovery following hospitalisation in the UK: A prospective observational study. *Lancet Respir Med* **2022**, *10*, 761–775, doi:10.1016/s2213-2600(22)00127-8.

### 2.10. Effects of Moderate-Intensity Continuous and High-Intensity Interval Training on Gut Microbiota Composition in Type 2 Diabetes Mellitus

Juan Corral-Pérez ^1,^*, Jesus G. Ponce-Gonzalez ^1^, Alberto Marín-Galindo ^1^, Manuel Costilla ^1^, Adrián Montes-de-Oca-García ^1^, Andrea González-Mariscal ^1^, Julio Plaza-Díaz ^2^ and Cristina Casals ^1,^*

^1^ ExPhy Research Group, University of Cádiz, Spain; juan.corral@uca.es; jesusgustavo.ponce@uca.es; alberto.marin@uca.es; manueljesus.costilla@uca.es; adrian.montesdeoca@uca.es; andrea.gonzalezmariscal@alum.uca.es; cristina.casals@uca.es

^2^ Department of Biochemistry and Molecular Biology II, University of Granada, Spain; jrplaza@ugr.es

* Correspondence: juan.corral@uca.es; cristina.casals@uca.es

**Abstract:** Type 2 diabetes (T2D) patients have an altered dysbiotic gut microbiota that increases fat mass and insulin resistance (1). Despite some articles have shown a positive relationship between physical exercise and gut microbiota (2), research in T2D is scarce. This study aims to compare the effects of 12-week High-Intensity Interval (HIIT) and Moderate Intensity Continuous (MICT) training programs on skeletal muscle mitochondrial function in adults with T2D. This randomized controlled trial was conducted with 33 participants with T2D, aged between 40 and 65 years (57.56 ± 7.32 years), with overweight/obesity (Body Mass Index = 33.50 ± 7.02 kg/m^2^), no antibiotics taken in the last 6 months, and no diseases that could alter the measurements (irritable bowel). Participants were randomly assigned to three groups: HIIT (10 × 1 intervals of pedalling at 90% of peak power output, n = 11, including 3 females), MICT (continuous pedalling at 10% above the first ventilatory threshold for 50 min, n = 12, including 5 females), or a control group (n = 10, including 2 females). Both exercise groups engaged in three sessions per week for 12 weeks. Diet was controlled during the past 7 days of the faecal sample collection using a 7-day dietary rec-ord. Faecal samples were collected both pre- and post-intervention for microbiome composition analysis. DNA extraction utilized the mini QIAamp DNA stool kit (QIAGEN, Barcelona, Spain), while quantification employed the NanoDrop ND-1000 spectrophotometer (Thermo Fisher Scientific, DE, USA). Statistical analysis employed mixed factorial ANOVA with Bonferroni post hoc comparisons to evaluate the changes in microbial mean relative abundance. Additionally, correlations (Pearson’s R) between changes in microbiota distribution and species richness were used. A time effect was observed for Escherichia fergusoni_ATCC35466 (F = 8.58, η2p = 0.15, *p* = 0.032), with the HIIT group showing a significant increase (+6.2 ± 4.2%). A time-group effect was found for Marseillibacter massiliensis (F = 2.58, η2p = 0.210, *p* = 0.032), with both HIIT (+10.2 ± 4.3%) and MICT (+12.3 ± 3%) groups exhibiting significant increases. The increase in Marseillibacter massiliensis correlated significantly with species richness (r = 0.58, *p* = 0.042). This study demonstrates that both HIIT and MICT induce an increase in Marseillibacter massiliensis, associated with enhanced species richness. Given that species richness has been associated with lower insulin resistance and low-grade inflammation in T2D patients (3) this enhancement could potentially ameliorate these two important markers for T2D. However, it is essential to note that HIIT training also elevates the mean relative abundance of Escherichia fergusoni_ATCC35466, linked to a higher T2D risk (4), which may offset the benefits of increased species richness.

**Keywords:** intestinal bacterial communities; intestinal health; metabolic diseases; physical activity.

**Funding:** This preliminary study is part of the EDUGUTION/APETEX randomized controlled trials funded by the Ministry of Science and Innovation (10.13039/501100011033PID2019-110063RA-I00, PID2020-120034RA-I00).

**References**

1. Le Chatelier, E.; Nielsen, T.; Qin, J.; Prifti, E.; Hildebrand, F.; Falony, G.; Almeida, M.; Arumugam, M.; Batto, J.-M.; Kennedy, S.; et al. Richness of Human Gut Microbiome Correlates with Metabolic Markers. *Nature* **2013**, *500*, 541–546, doi:10.1038/nature12506.
2. Rojas-Valverde, D.; Bonilla, D.A.; Gómez-Miranda, L.M.; Calleja-Núñez, J.J.; Arias, N.; Martínez-Guardado, I. Examining the Interaction between Exercise, Gut Microbiota, and Neurodegeneration: Future Research Directions. *Biomedicines* **2023**, *11*, 2267, doi:10.3390/biomedicines11082267.
3. Tilg, H.; Zmora, N.; Adolph, T.E.; Elinav, E. The Intestinal Microbiota Fuelling Metabolic Inflammation. *Nat. Rev. Immunol.* **2020**, *20*, 40–54, doi:10.1038/s41577-019-0198-4.
4. Wu, X.; Park, S. Fecal Bacterial Community and Metagenome Function in Asians with Type 2 Diabetes, According to Enterotypes. *Biomedicines* **2022**, *10*, 2998, doi:10.3390/biomedicines10112998.

### 2.11. Effects of 12-Weeks of Moderate-Intensity Continuous and High-Intensity Interval Training on Mitochondrial Function in Type 2 Diabetes

Manuel Costilla ^1,^*, Cristina Casals ^1^, Alberto Marín-Galindo ^1^, Sonia Ortega-Gómez ^1^, Laura Ávila-Cabeza-de-Vaca ^1^, Steen Larsen ^2^, Jesus G. Ponce-Gonzalez ^1^ and Juan Corral-Pérez ^1,^*

^1^ ExPhy Research Group, University of Cádiz, Spain; manueljesus.costilla@uca.es; cristina.casals@uca.es; alberto.marin@uca.es; sonia.ortega@uca.es; laura.avilacabezadevaca@alum.uca.es; jesusgustavo.ponce@uca.es; juan.corral@uca.es

^2^ Xlab, Center for Healthy Aging, Department of Biomedical Sciences, University of Copenhagen, Copenhagen Denmark; stelar@sund.ku.dk

* Correspondence: manueljesus.costilla@uca.es; juan.corral@uca.es

**Abstract:** The pathophysiology of Type 2 Diabetes Mellitus (T2D) involves skeletal muscle mitochondrial dysfunction and insulin resistance (1). Despite it is well known that exercise can improve mitochondrial function, few studies compare the efficacy of different exercise models, including High-Intensity Interval Training (HIIT) and Moderate-Intensity Continuous Training (MICT). This study aims to compare the effects of 12-week HIIT and MICT training programs on skeletal muscle mitochondrial function in adults with T2D. This randomized controlled trial was conducted with 32 participants with T2D, aged between 40 and 65 years (56.31 ± 7.46 years), with overweight/obesity (Body Mass Index = 33.73 ± 6.04 kg/m^2^), and no diseases that could alter the measurements. Participants were assigned to three groups: HIIT (10 × 1 intervals of pedalling at 90% of peak power output n = 9, including 3 females), MICT (continuous pedalling at 10% above the first ventilatory threshold for 50 min, n = 13, including 5 females), or a control group (n = 9, including 2 females). Both exercise groups engaged in three sessions per week for 12 weeks. Mitochondrial respiration was assessed in permeabilized skeletal muscle fibres using an Oxygraph-2k. The testing protocol included the sequential addition of pyruvate (2 M) + malate (0.4 M) + glutamate (2 M) and ADP (0.5 M) + Mg (0.3 M) to stimulate the electron transport chain complex I (CI). Succinate (1 M) was subsequently added to stimulate maximal respiratory capacity (CI + II). Statistical analysis employed mixed factorial ANOVA with Bonferroni post hoc comparisons. No significant differences in CI or CI + II were observed between groups at the pre-intervention assessment. For CI activity, there was a time effect for CI activity (F = 12.58, η2p = 0.30, *p* = 0.001) with a significant increase in the MICT group after the intervention (18.82 ± 10.80 vs. 37.03 ± 17.21 pmol s^−1^ [mg w.w.]^−1^), with no significant time-group effect. For CI + II, a significant time effect was shown (F = 29.87, η2p = 0.516, *p* < 0.001), a trend to signification in time-group effect (F = 2.58, η2p = 0.156, *p* = 0.69) with both HIIT and MICT groups exhibited significant increases in CI + II activity (HIIT: 38.22 ± 12.38 vs. 66.88 ± 27.84 pmol s^−1^ [mg w.w.]^−1^, *p* = 0.001; MICT: 38.46 ± 17.02 vs. 75.40 ± 20.98 pmol s^−1^ [mg w.w.]^−1^, *p* < 0.001). This preliminary study suggests that both HIIT and MICT effectively enhance maximal respiratory capacity in adults with T2D. However, MICT appears to be more effective as it significantly increases the capacity of both CI and CI + II, while HIIT primarily improves CI + II.

**Keywords:** muscle metabolism; high-resolution respirometry; aerobic exercise; exercise intensity; muscle biopsy.

**Funding:** This preliminary study is part of the EDUGUTION/APETEX/MITOX randomized controlled trials funded by the Ministry of Science and Innovation (10.13039/501100011033PID2019-110063RA-I00, PID2020-120034RA-I00) and INiBICA (PP11-007-2023).

**References**

1. Pinti, M. V.; Fink, G.K.; Hathaway, Q.A.; Durr, A.J.; Kunovac, A.; Hollander, J.M. Mitochondrial Dysfunction in Type 2 Diabetes Mellitus: An Organ-Based Analysis. *Am J Physiol Metab* **2019**, *316*, E268–E285, doi:10.1152/ajpendo.00314.2018.

### 2.12. 1RM Prediction Using Different Models and MVT in Female Athletes

Emanuele Dello Stritto ^1^, Ruggero Romagnoli ^1^ and Maria Francesca Piacentini ^1,^*

^1^ Department of Human Movement and Health Sciences, University of Rome “Foro Italico”, 00135 Rome, Italy; e.dellostritto@studenti.uniroma4.it; mariafrancesca.piacentini@uniroma4.it

* Correspondence: mariafrancesca.piacentini@uniroma4.it; Tel.: +39-0636733245

**Abstract:** The load-velocity (LV) relationship is commonly used to estimate one repetition maximum (1RM) and to prescribe and monitor velocity-based resistance training sessions (1,2). The aim of the study was to evaluate differences between measured 1RM and 1RM estimated from different models, using LV data. 26 resistance-trained females (age: 24.2 ± 3.5 years; BW: 57.11 ± 5.1 kg; height: 164 ± 4.50 cm) performed a maximal incremental test (1RM) on the Back Squat. Mean propulsive velocity (MPV) of the barbell was recorded by a linear position transducer (Vitruve). Load-velocity profiles were constructed for each subject using linear and quadratic regression models to estimate 1RM using different minimum velocity thresholds (MVT): the Generalized MVT (GMVT), where the MVT used to predict 1RM was 0.30 m·s^−1^; the Individualized MVT (IMVT) corresponding to the MVT measured experimentally for each subject, the Equation MVT (EQMVT), using the MVT provided by the regression model of each subject. Repeated measures ANOVA was used to investigate differences in 1RM prediction between the three equations of each model. Furthermore, the agreement between measured 1RM and predicted 1RM was explored using the Bland-Altman plots. Lastly, Pearson correlation coefficients (r) was investigated. Our analysis revealed how real 1RM and 1RM predicted by GMVT were significantly different in both linear and quadratic equations. Meanwhile, 1RM provided by IMVT and real 1RM showed no significant difference in both cases. Furthermore, no significant difference was found between real 1RM and 1RM provided by EQMVT in linear equations, but, differences were found in quadratic equations. Based on our analysis we can point out that IMVT is a better predictor of real 1RM when a quadratic equation is used. Meanwhile, no differences between IMVT and EQMVT were found with linear equation condition. Overall, we suggest to use IMVT when possible, with no distinction between linear or quadratic equations. On the other hand, if IMVT cannot be measured, EQMVT could be utilized, but only using a linear equation.

**Keywords:** load-velocity relationship; 1RM prediction; 1RM estimation; resistance training; velocity-based training.

**Funding:** This research received no external funding.

**References**

1. Marston, K.J.; Forrest, M.R.L.; Teo, S.Y.M.; Mansfield, S.K.; Peiffer, J.J.; Scott, B.R. Load-velocity relationships and predicted maximal strength: A systematic review of the validity and reliability of current methods. *PLoS One* **2022**, *17*, e0267937, doi:10.1371/journal.pone.0267937.
2. Thompson, S.W.; Rogerson, D.; Ruddock, A.; Greig, L.; Dorrell, H.F.; Barnes, A. A Novel Approach to 1RM Prediction Using the Load-Velocity Profile: A Comparison of Models. *Sports* **2021**, *9*, 88, doi:10.3390/sports9070088.

### 2.13. Pro- and Anti-Inflammatory Cytokine Responses Following Three Distinct Exercise Conditions in Young Active Males

Panagiotis Ferentinos ^1,^*, Michelle Swainson ^2^, Matthew Lees ^3^, Antonis Elia ^4^, Costas Tsakirides ^1^ and Theocharis Ispoglou ^1^

^1^ Carnegie School of Sport, Leeds Beckett University, Leeds, UK; P.Ferentinos@leedsbeckett.ac.uk; c.tsakirides@leedsbeckett.ac.uk; t.ispoglou@leedsbeckett.ac.uk

^2^ Lancaster Medical School, Faculty of Health and Medicine, Lancaster University, Lancaster, UK; m.swainson1@lancaster.ac.uk

^3^ Faculty of Kinesiology and Physical Education, University of Toronto, Toronto, Ontario, Canada; matthew.lees@utoronto.ca

^4^ Division of Environmental Physiology, Royal Institute of Technology, Stockholm, Sweden; antonise@kth.se

* Correspondence: P.Ferentinos@leedsbeckett.ac.uk

**Abstract:** Acute exercise triggers the synthesis of several pro- and anti-inflammatory markers via skeletal muscle contraction. Physiological responses to different types of exercise vary, influencing the production of pro- and anti-inflammatory cytokines (1). This study aimed to compare the acute effects of different exercise conditions on interleukin (IL)-6, IL-10 and C-reactive protein (CRP). Ten young active healthy adults (24.6 ± 2.1 years) completed three different exercise protocols: (a) high intensity interval exercise (HIIE, 4 sets × 4 min at 85–95% HRmax with 3 min recovery at 50–55% HRmax), (b) moderate-intensity continuous exercise (MICON, 70–80% HRmax), and (c) whole-body resistance exercise (RE), consisting of six exercises (3 sets of 10 repetitions at 70% of 1RM with 90 s recovery between sets and 3 min between exercises). A venous blood sample was taken pre-exercise, at 10 min post-exercise and at 2 h post-exercise. All laboratory visits took place a week apart, in a randomised cross-over fashion. There was a significant difference in percentage change of IL-6 from pre-exercise to 10 min post-exercise between the three exercise conditions, (*p* = 0.013). Post-hoc analysis showed that there was a large significant difference between HIIE and RE (137.1 ± 38.6% vs. 41.9 ± 9.1%, *p* = 0.03, *d* = 1.07), a moderate difference between HIIE and MICON (137.1 ± 38.6% vs. 64.4 ± 26.2%, *p* > 0.05, *d* = 0.70) and a small difference between MICON and RE (64.4 ± 26.2% vs. 41.9 ± 9.1% *p* > 0.05, *d* = 0.36). Percentage change of IL-6 from pre-exercise to 2 h post-exercise was not statistically significant (*p* > 0.05). For IL-10 the percentage change from pre-exercise to 10 min post-exercise differed significantly between conditions, *p* = 0.003. Post-hoc comparisons revealed a significant large difference between HIIE and MICON (56.0 ± 25.1% vs. −23.5 ± 8.4%, *p* = 0.002, *d* = 1.34), a large difference between HIIE and RE (56.0 ± 25.1% vs. −4.2 ± 5.5%, *p* > 0.05, *d* = 1.04) and a large difference between MICON and RE (−23.5 ± 8.4% vs. −4.2 ± 5.5%, *p* > 0.05, *d* = 0.86). No statistically significant differences were observed for the percentage changes from pre-exercise to 2 h post-exercise (*p* > 0.05). For CRP no significant differences were found for the percentage changes in any time-point between the exercise conditions (*p* > 0.05). The results of the present study indicate that IL-6 and IL-10 responses depend on the exercise modality, with HIIE appearing to induce the largest increases.

**Keywords:** high intensity interval exercise; resistance exercise; inflammation; interleukin, myokine.

**Funding:** This research received no external funding.

**References**

1. Brown, W.M; Davison, G.W; McClean, C.M; Murphy, M.H. A Systematic Review of the Acute Effects of Exercise on Immune and Inflammatory Indices in Untrained Adults. *Sports Med Open* **2015**, *1*, 35.

### 2.14. Post-Activation Potentiation Mechanisms with Temperature Influence: A Randomized Study

Joana Ferreira ^1^, Carolina Vila Chã ^2^, Pedro Fonseca ^3^, Miguel Correia ^4^ and Filipe Conceição ^5,^*

^1^ Faculty of Sport, University of Porto, Portugal, up201706385@up.pt

^2^ Polytechnic of Guarda, CIDESD, Portugal, cvilacha@ipg.pt

^3^ LABIOMEP, Portugal, pedro.labiomep@fade.up.pt

^4^ Faculty of Engineering, University of Porto, Portugal, mcorreia@fe.up.pt

^5^ LABIOMEP, CIFI2D, Faculty of Sport, University of Porto, Portugal, filipe@fade.up.pt

* Correspondence: filipe@fade.up.pt

**Abstract:** Post-activation potentiation (PAP) is defined by Sale (1) as an increase in the force/torque of an electrically evoked contraction after submaximal and maximal conditioned contractions. In other words, it is an increase in peak isometric force contraction or low frequency/torque tetanic force after (i) a series of evoked stimuli, (ii) an evoked tetanic contraction, or (iii) a maximal voluntary contraction (MVC) (e.g., conditioned contraction). The study and analysis of the relationship between the physiological phenomena associated with PAP and temperature manipulation could be an asset for the targeted intervention of professionals, particularly coaches and strength and conditioning coaches. The objectives were to identify the relevance of this phenomenon for the increase of performance in explosive sports athletes and explore the relationship between temperature and the occurrence of the PAP phenomenon. For that, a quantitative, cross-sectional, descriptive-correlational study with 14 athletes was carry out at the Biomechanics Laboratory of Porto (LABIOMEP). The protocols consisted of 3MVC before 10 stimuli with the current defined by the analysis of the M-wave and H-reflex at the beginning of the session. Then, the cooling or heating packs (depending on the protocol) were placed around the leg for 5 min, or the subjects remained that time at room temperature (protocol control). The protocol ended with the performance, again, of the 10 stimuli under the same conditions of current. With that, no significant differences were found in the variables in the PAP activation exploration protocol except for the MVC values (MVC with *p* = 0.041; H-reflex with *p* = 0.362; M-wave with *p* = 0.258). Furthermore, no advantages were found in the manipulation of temperature under the mechanisms of activation of this phenomenon. In conclusion, further studies are needed to understand the activation of PAP mechanisms and the importance to sports performance and if cold and local heat applications genuinely modify the mechanisms of PAP activation with the exploration of different temperature manipulation methods and different exposure times to these instruments.

**Keywords:** potentiation post-activation; temperature; h-reflex; maximal voluntary contraction; high performance training.

**Funding:** This research received no external funding.

**References**

1. Sale, D. G. Postactivation potentiation: Role in human performance. *Exerc Sport Sci Rev* **2002**, *30*, 138–143.

### 2.15. Comparative Effects on Quadriceps Function and Posterior Chain Flexibility of an Acute Bout of Self Myofascial Release on Plantar Fascia with Auramat^®^ vs. Traditional Warm-Up

Danilo Gaias ^1^, Antonio Martínez-Serrano ^1,2,3^ and Luis Manuel Martiínez-Aranda ^4,5,^*

^1^ UCAM Research Center for High Performance Sport, Catholic University of Murcia, Murcia, Spain.; danilo.gaias26@gmail.com; amartinez30@ucam.edu

^2^ Strength and Conditioning Society, Murcia, Spain

^3^ Faculty of Sport Sciences, Catholic University of Murcia, Murcia, Spain

^4^ Physical and Sports Performance Research Centre, Faculty of Sports Sciences, Pablo de Olavide University, Seville, Spain; lmmarara@upo.es

^5^ SEJ-680: Science-Based Training (SBT) Research group, Pablo de Olavide University, Seville, Spain

* Correspondence: lmmarara@upo.es; Tel.: (+34)-954977599

**Abstract:** Self-myofascial release (SMFR) is a treatment that can be done with a large variety of tools and whose main benefits are enhanced recovery and increased flexibility without the impairment of performance parameters (1). Many natural surface SMFR tools have entered the market in recent years, Auramat^®^ being one of them, but they have not been investigated until now (1). The aim of this study was to determine the effects of Auramat^®^ Platform (AUR) on posterior chain flexibility and knee extensor’s function. A second aim was to compare those effects with the effects of a traditional warm-up (TW) comprising of low-intensity cardiovascular exercise, dynamic stretching, and bodyweight resistance training (2). 20 healthy physically active subjects (12 male and 8 female subjects; age = 27.2 ± 4.98 years; weight = 76.4 ± 12.14 kg; height = 1.73 ± 0.09 m), participated in this research. The study was a randomized, counterbalanced, cross-over design were participants attended the laboratory three times over a two-weeks period. The first week took place the familiarization session to prepare the subject to perform the tests. The second week the groups that were randomly assigned at two different interventions performed the two protocols separated by 48h. During the testing sessions Straight Leg Raise Test, knee extensors MVIC and RFD were performed only on the dominant limb pre and post intervention. A 2-way ANOVA with repeated measures for two factors (group and time) was performed on all dependent variables recorded in the precondition and postcondition. No significant differences in MVIC and RFD for group (*p* = 0.910), time (*p* = 0.240), group*time (*p* = 0.410) were found. Both groups improved posterior chain flexibility after the intervention (*p* < 0.001; **↑**5.75%). RPE for TW showed significant higher values compared to AUR RPE (*p* < 0.001; ES = 2.32; TW = 4.3 ± 1.45 vs. AUR = 1.55 ± 0.82). A bout of SMFR on the plantar fascia with AUR increased flexibility while maintaining knee extensors force production. TW and AUR seem to have the same effects on flexibility and force production, with AUR being a less time-consuming strategy with less perceived effort.

**Keywords:** superficial back line; knee extensor; range of motion; performance.

**Funding:** This research received no external funding.

**References**

1. Ferreira, R.M.; Martins, P.N.; Goncalves, R.S. Effects of Self-myofascial Release Instruments on Performance and Recovery: An Umbrella Review. *Int J Exerc Sci* 2022, *15,* 861–83.
2. Safran, M.R.; Seaber, A.V.; Garrett, W.E.J. Warm-up and muscular injury prevention. An update. *Sports Med Auckl NZ* 1989, *8,* 239–49.

### 2.16. Changes in Football Players’ Competitive Level of Exertion throughout the Season Modifies Correlation Strength between Mechanical and Subjective Load Variables

Carlos Galiano ^1,^* and Iván Asín-Izquierdo ^1,2,3^

^1^ Physical Performance and Sports Research Center, Department of Sports and Computer Sciences, Faculty of Sport Sciences, Pablo de Olavide University, Seville, Spain; galianodelarocha@gmail.com; ivanasizq@gmail.com

^2^ Department of Biomedical Sciences, Faculty of Medicine and Health Sciences, University of Alcalá, Madrid, Spain

^3^ Department of Musical, Plastic and Corporal Expression, Faculty of Social and Human Sciences, University of Zaragoza, Teruel, Spain

* Correspondence: galianodelarocha@gmail.com; ivanasizq@gmail.com

**Abstract:** Session-RPE (RPEs) method has been demonstrated to be valid, reliable and very useful on the field scenarios. Studies show that the correlation level of RPEs with mechanical variables is high and significant. However, due to the changes in physical and mental exertion that occur throughout the season, we do not know if the correlation value changes as the team approaches the end of the season. The aim of the study is to test if the correlation degree of RPEs with mechanical variables changes throughout the season in its three stages: preseason, regular season, and playoffs. The study was conducted in a Spanish semi-professional football team during the 2020/2021 season. The team consisted of 22 football players (23.21 ± 04.22 years, 74.98 ± 5.78 kg and 1.79 ± 0.06 m). Data were recorded throughout all training sessions and competitive matches of the season. The Borg subjective scale was used in its version: RPE 6–20 and this number was multiplied for session or match (time played) duration (min) in order to get RPEs. RPE data were requested prior to the session at the same time as the GPS-GNSS waistcoats were assigned using Playertek+ Catapult devices. GPS-GNSS data were loaded into the Playertek software and aggregated into spreadsheets. Correlations analyzed were: week-RPEs with total distance (TD (m)), high-intensity distance (HS (>21 km/h)), acceleration (ACC (number)) and deceleration (DEC (number). Data were processed globally and in three periods of the season (pre-season, regular season and playoffs) RPEs showed significant correlation with TD, HS, ACC y DEC (r = 0.82 to 0.89; *p* < 0.001) for whole season data. During preseason, RPEs showed significant correlation for TD, HS, ACC and DEC (r = 0.54 to 0.83; *p* < 0.001). On regular season, TD, HS, ACC and DEC correlated with RPEs (r = 0.56 to 0.79; *p* < 0.001) and, during playoffs, the strength of the correlation was even larger (r = 0.62 to 0.91; *p* < 0.001). RPEs is a low cost, viable and reliable tool to estimate on-field mechanical variables during the season. However, coaches must be cautions since changes in players’ level of exertion throughout season might alter the strength of correlation between RPEs and mechanical load variables.

**Keywords:** RPE; GPS-GNSS; workload; external load; internal load**;** monitoring; soccer.

**Funding:** This research received no external funding.

**References**

1. Haddad, M.; Stylianides, G.; Djaoui, L.; Dellal, A.; Chamari, K. Session-RPE method for training load monitoring: validity, ecological usefulness, and influencing factors. *Front Neurosci* **2017**, *11*, 612.
2. Impellizzeri, F.M.; Rampinini, E.; Coutts, A.J.; Sassi, A.L.D.O.; Marcora, S.M. Use of RPE-based training load in soccer. *Med Sci Sports Exerc* **2004**, *36*, 1042–1047.
3. Marynowicz, J.; Kikut, K.; Lango, M.; Horna, D.; & Andrzejewski, M. Relationship between the session-RPE and external measures of training load in youth soccer training. *J Strength Cond Res* **2020**, *34*, 2800–2804.
4. Casamichana, D.; Castellano, J.; Calleja-Gonzalez, J.; San Román, J.; Castagna, C. Relationship between indicators of training load in soccer players. *J Strength Cond Res* **2013**, *27*, 369–374.

### 2.17. Has Covid-19 Pandemic Influenced the Performance of Top-Class Duathletes of the World Duathlon Championship?

Pablo García-González ^1^ and José Antonio González-Jurado ^1,^*

^1^ University Pablo de Olavide, Seville, Spain; pablogartri@gmail.com

* Correspondence: pablogartri@gmail.com

**Abstract:** Triathlon is the most studied multi-sport from the ITU. Triathlon performance has been the subject of extensive analysis in other Championships (1), as well as in other distances, including Long Distance (2), Ironman (3) or the Triple or Deca Iron, not belonging to ITU the last two (4). World Championships are attractive to the scientific community and have been investigated in a wide range of sports, duathlon among them (5). The ITU World Duathlon Championship is the top championship for this sport. The influence of COVID-pandemic on duathlon is not yet clear. The aim of the present study is to provide a comprehensive analysis of the effect of COVID-19 pandemic on performance in the World Duathlon Championship focusing on male and female categories; The dataset for this study was obtained from the ITU World Triathlon Series (WTS) website (http://wts.triathlon.org/). Individual discipline times and overall times from 2018 and 2022 were collected for analysis, excluding 2020 due to the COVID-19 outbreak. The total number included athletes in this study was 265 subjects (177 males and 88 females). The mean age of subjects overall was 27.01 for males and 29.11 for females. The Student’s t-test for independent samples comparing sex was used for normal variables, whereas the Mann-Whitney U-test was used for non-normal variables.; It displayed significant changes in performance in most of the analyzed variables. The analysis revealed significant changes in many studied variables for either sex. Of those, cycling performance had greater values after COVID-19 pandemic for either sex (*p*-values < 0.000) whereas swimming, running and final performance showed worse values (*p*-values < 0.000 for the first two and 0.002; 0.335 for final performance. Women’s performance decreased less than men’s in second running performance and final performance (*p*-values < 0.01; <0.000). These results suggest that athletes participating in the World Duathlon Championship experienced a decrease in their performance after COVID-19 pandemic in all variables except for cycling and first transition performance. This decline is comparatively less pronounced in the women’s category.

**Keywords:** SARS-CoV-2; World Championship; transition.

**Funding:** This research received no external funding.

**References**

1. Etter, F.; Knechtle, B.; Bukowski, A.; Rüst, C.A.; Rosemann, T.; Lepers, R. Age and gender interactions in short distance triathlon performance. *J Sports Sci* **2013**, *31*, 996–1006.
2. Knechtle, B.; Rüst, C.A.; Knechtle, P.; Rosemann, T.; Lepers, R. Age-related changes in ultra-triathlon performances. *Extreme Physiol Med* **2012**, *1*.
3. Gallmann, D.; Knechtle, B.; Rüst, C.A.; Rosemann, T.; Lepers, R. Elite triathletes in “Ironman Hawaii” get older but faster. *Age* **2014**, *36*, 407–416.
4. Lenherr, R.; Knechtle, B.; Rüst, C.; Rosemann, T.; Lepers, R. From Double Iron to Double Deca Iron Ultra-Triathlon—A Retrospective Data Analysis from 1985 to 2011. *Phys Cult Sport Stud Res* **2012**. *54*, 55–67.
5. Nikolaidis, P.T.; Villiger, E.; Knechtle, B. Participation and performance trends in the ITU Duathlon World Championship from 2003 to 2017. *J Strength Cond Res* **2018**, *00*, 1–7.

### 2.18. Has COVID-19 Pandemic Influenced the Performance of Top-Class Triathletes in the Sprint Distance of the World Triathlon Series?

Pablo García-González ^1,^* and José Antonio González-Jurado ^1^

^1^ University Pablo de Olavide, Seville, Spain; pablogartri@gmail.com

* Correspondence: pablogartri@gmail.com

**Abstract:** World Championships are attractive to the scientific community, and have been investigated in a wide range of sports (1), sometimes related somehow to triathlon (2) but also very similar sports as duathlon (3) The World Triathlon Series (WTS) is the top championship for triathlon in sprint (SPD) distance. Sex differences in triathlon performance have been found to depend on discipline and distance, as previous studies have shown (4). Lepers & Stapley (5) have asserted that the sex gap is narrower in the Olympic distance when compared to other larger distances. Even though there are indeed studies that demonstrate the harm the virus caused to endurance athletes, other studies provide overwhelmingly positive evidence regarding the influence of lockdown. For instance, it has been demonstrated that lockdown was beneficial in maintaining the physical fitness of gymnasts in the United Kingdom and was perceived as time for rest and recovery. The influence of COVID-pandemic on Triathlon is not yet clear. The aim of the present study is to provide a comprehensive analysis of the effect of COVID-19 pandemic on performance in the WTS focusing on male and female categories; The dataset for this study was obtained from the ITU World Triathlon Series (WTS) website (http://wts.triathlon.org/). Individual discipline times and overall times from 2018 and 2022 were collected for analysis, excluding 2020 due to the COVID-19 outbreak. The total number included athletes in this study was 1153 subjects (606 males and 547 females). The mean age of subjects overall was 26.28 for males and 26.90 for females. The Student’s t-test for independent samples comparing sex was used for normal variables, whereas the Mann-Whitney U-test was used for non-normal variables.; It displayed significant changes in performance in most of the analyzed variables. Men and women improved all the analyzed variables that showed significant differences. All variables improved for men, women and when both were analyzed together (except for swimming performance in women). Men showed greater change values in swimming and cycling performance (*p*-values ˂ 0.000) whereas women showed them in running performance (*p*-value ˂ 0.000).; These results suggest that triathletes participating in WTS of both sex were able to not only maintain but also improve their final performance level despite COVID-19 pandemic.

**Keywords:** SARS-CoV-2; World Championship; transition.

**Funding:** This research received no external funding.

**References**

1. De La Rubia, A.; Bjørndal, C.T.; Sánchez-Molina, J.; Yagüe, J.M.; Calvo, J.L.; Maroto-Izquierdo, S. The relationship between the relative age effect and performance among athletes in World Handball Championships. *PLoS ONE* **2020**, *15*, 1–22.
2. Haupt, S.; Knechtle, B.; Knechtle, P.; Rüst, C.A.; Rosemann, T.; Lepers, R. The age-related performance decline in ultraendurance mountain biking. *Res Sports Med* **2013**, *21*, 146–158.
3. Nikolaidis, P.T.; Villiger, E.; Knechtle, B. Participation and performance trends in the ITU Duathlon World Championship from 2003 to 2017. *J Strength Cond Res* **2018**, *00*, 1–7.
4. Lepers, R. Sex difference in triathlon performance. *Front Physiol* **2019**, *10*, 1–7.
5. Lepers, R.; Stapley, P.J. Differences in gender and performance in off-road triathlon. *J Sports Sci* **2010**, *28*, 1555–1562.

### 2.19. Physiological Determinants in Rhythmic Gymnastics: A Systematic Review with Training Considerations

Vasiliki Gaspari ^1,^*, Gregory, C. Bogdanis ^1^, Ioli Panidi ^1^, Andreas Konrad ^2^, Anastasia Donti ^1^, Gerasimos Terzis ^1^ and Olyvia Donti ^1^

^1^ School of Physical Education & Sport Science, National and Kapodistrian University of Athens, 17237 Athens, Greece; vgaspari@phed.uoa.gr; gbogdanis@phed.uoa.gr; ipanidi@phed.uoa.gr; adonti@phed.uoa.gr; gterzis@phed.uoa.gr; odonti@phed.uoa.gr

^2^ Institute of Human Movement Science, Sport and Health, University of Graz, Graz, Austria; andreas.konrad@uni-graz.at

* Correspondence: vgaspari@phed.uoa.gr; Tel.: +30-21-0973-5494

**Abstract:** Rhythmic gymnastics (RG) is an “early specialization” sport, characterized by high training load (1), and participation in year-round intense training from an early age (i.e., 7–8 years old). Physical conditioning is an integral part of rhythmic gymnasts’ preparation (2), aiming to improve performance while reducing the risk of sports-related injuries. However, evidence on the development of physical fitness parameters in RG is limited. The purpose of this systematic review was to provide an overview of fitness parameters related with RG and to examine if they depend on gymnasts’ age, and level of performance. In addition, the association between fitness tests scores and rhythmic gymnastics performance were examined. PubMed, Scopus, and Sport Discus databases were searched using a structured algorithm. Cross-sectional and intervention studies were included in this systematic review. No restrictions to language or date of publication were applied. From the 586 records retrieved, 47 studies were included in this study (N = 2087 participants, aged 13.6 ± 1.8 years). The included studies examined flexibility (n = 16), muscle power (n = 21), balance (n = 11), aerobic capacity (n = 9), coordination (n = 11), muscle endurance (n = 8), muscle strength (n = 2), sprint speed (n = 3), and agility (n = 4), assessed using both, general and sport-specific tests. It was found that, higher-level gymnasts outperformed lower-level gymnasts in all the fitness parameters examined and the same was found for rhythmic gymnasts compared to athletes from other sports or controls. Furthermore, older gymnasts demonstrated better fitness scores than younger gymnasts. No difference between gymnasts’ levels was found in agility when measured using a sport-specific shuttle run test. General tests were not sensitive enough to detect differences between performance levels and their results correlated poorly with performance. From a young age, performance scores were associated with flexibility, leaping ability, balance, aerobic capacity, coordination, and agility. Muscular strength, speed, and agility are largely under-researched in RG. Although flexibility, lower limb muscle power, aerobic capacity, balance, coordination, speed, and agility are important performance determinants in RG, there is limited evidence on the possible role of muscular strength, endurance, speed, and agility development on injury risk. This is important because of the demands of training in RG from a very young age, placing high stress on skeletally immature athletes, who possibly have sub-optimal strength levels. Further research should examine developmentally appropriate programs of muscular fitness development in youth rhythmic gymnasts.

**Keywords:** strength; physical abilities; physical fitness, development.

**Funding:** This research received no external funding.

**References**

1. Douda, H.; Toubekis, A.; Avloniti, A.; Tokmakidis, S. Physiological and anthropometric determinants of rhythmic gymnastics performance. *Int J Sports Physiol Perform* **2008**, *3*, 41–54.
2. Donti, O.; Bogdanis, G. C.; Kritikou, M.; Donti, A.; Theodorakou, K. The Relative Contribution of Physical Fitness to the Technical Execution Score in Youth Rhythmic Gymnastics. *J Hum Kinet* **2016**, *51*, 143–152.

### 2.20. Comparison of Lifestyle Factors among Students of Three Tertiary Education Institutions

Kalliopi Georgakouli ^1^ and Athanasios Z. Jamurtas ^2,^*

^1^ Department of Nutrition and Dietetics, University of Thessaly; kgeorgakouli@uth.gr

^2^ Department of Physical Education and Sport Science, University of Thessaly; ajamurt@uth.gr

* Correspondence: ajamurt@uth.gr; Tel.: +30-24310-47054

**Abstract:** Many higher education students have an unhealthy lifestyle, leading to, among others, reduced academic performance. The aim of this study was to compare body mass index (BMI), alcohol use parameters and physical activity levels among higher education students in different disciplines, related or not to health promotion through lifestyle changes. One hundred students (age 20.9 ± 0.2; BMI 22.2 ± 0.2) in physical education and sport science (PESS), 100 students (age 21.3 ± 0.5; BMI 22.8 ± 0.3) in nutrition and dietetics (ND), and 100 students (age 20.5 ± 0.3; BMI 23.5 ± 0.3) in departments whose discipline is not related to health promotion through lifestyle changes (engineering, accounting) (EA) completed a demographic data form, and questionnaires on alcohol consumption, Alcohol Use Disorder Identification Test (AUDIT; 1), and physical activity levels (International Physical Activity Questionnaire—IPAQ; 2). Results are shown as mean ± standard error, statistical significance threshold is *p* < 0.05. It appeared that the BMI of EA students was significantly higher than that of PESS students. Regarding the physical activity levels, ND students had lower IPAQ score, both compared to PESS students (1852 ± 182 vs. 3457 ± 250 MET-minutes/week; *p* = 0.001) and EA students (1852 ± 182 vs. 4086 ± 446 MET-minutes/week; *p* = 0.000). There was no difference in physical activity levels between PESS and EA students. Regarding alcohol, there was no difference in the number of drinks they reported to consume per day among all groups of students. However, ND students reported that they used to drink fewer drinks per week compared to both PESS students (2.1 ± 0.5 vs. 3.8 ± 4.5; *p* = 0.001) and EA students (2.1 ± 0.5 vs. 3.8 ± 4.5; *p* = 0.035). Nevertheless, the AUDIT score was <8 (indicating absence of alcohol use disorder) in all the three groups (PESS students 4.5 ± 0.4 vs. ND students 3.6 ± 0.3 vs. EA students 4.5 ± 0.4), among which no differences were observed. In conclusion, the discipline of studies could influence lifestyle parameters, in some cases in a positive way (e.g., limited alcohol use in ND students). More research on the association between discipline of studies and lifestyle parameters may contribute to targeted interventions to improve students’ health and academic performance.

**Keywords:** alcohol; physical activity; body mass index; academic performance; quality of life.

**Funding:** This research received no external funding.

**References**

1. Moussas, G.; Dadouti, G.; Douzenis, A.; Poulis E.; Tzelembis A.; Bratis D.; Christodoulou C.; Lykouras L. The Alcohol Use Disorders Identification Test (AUDIT): reliability and validity of the Greek version. *Ann Gen Psychiatry* **2009**, *8*, 11.
2. Papathanasiou, G.; Georgoudis, G.; Papandreou, M.; Spyropoulos, P.; Georgakopoulos, D.; Kalfakakou, V.; Evangelou, A. Reliability measures of the short International Physical Activity Questionnaire (IPAQ) in Greek young adults. *Hellenic J Cardiol* **2009**, *50*, 283–294.

### 2.21. Effects of Combined Uphill–Downhill Sprinting versus Resisted Sprinting Methods on Sprint Performance: A Systematic Review and Meta-Analysis

Maziar J. Hamad ^1,^*, Pedro E. Alcaraz ^1,2^ and Eduardo Sáez de Villareal ^3^

^1^ UCAM Research Center for High Performance Sport- Catholic University of Murcia (UCAM), Murcia Spain; mjhamad@alu.ucam.edu; palcaraz@ucam.edu

^2^ Faculty of Sport Sciences, Catholic University of Murcia (UCAM), Murcia, Spain

^3^ Physical Performance Sports Research Center (PPSRC), Universidad Pablo de Olavide, Seville, Spain

* Correspondence: mjhamad@alu.ucam.edu

**Abstract:** Two specific sprint training methods that are combined uphill–downhill sprinting (UDS) and resisted sprint training methods (RS). Both methods seem to improve sprint performance, but to the author’s knowledge a comparison does not exist investigating the differences between the two training protocols and traditional sprinting. The present systematic review and meta-analysis investigated sprint performance changes between combined uphill–downhill sprinting and resisted sprinting methods (sleds, cables/bands, vests, uphill) and how these compared with traditional sprinting. A literature search was performed on 19 December 2022, in the databases PubMed, SPORTDiscus, Web of Science and SCOPUS, which from 22 studies yielded a total of 24 eligible groups (UDS, *n* = 6; RS, *n* = 18). Studies that measured sprint performance, had a traditional sprinting control, and used either training intervention in healthy individuals of any age for ≥ 4 weeks were eligible for the meta-analysis. The change in sprint performance from baseline to post intervention was compared between the interventions and their traditional sprinting control group. Outcomes were expressed as standardized mean differences (SMD). The standardized changes in sprint performance between intervention groups and traditional-sprinting controls (negative in favour of intervention, positive in favour of traditional sprint) and 95% confidence interval (CI) were as follows: small for UDS (SMD − 0.41 [−0.79, −0.03]; *p* = 0.03), trivial for RS (SMD − 0.14 [−0.36, 0.07]; *p* = 0.19). Combined uphill–downhill sprinting was more effective than traditional sprinting, while resisted sprinting was not. It appears that resisted sprint interventions do not increase sprint performance any more than traditional sprinting. Sub- group analysis and meta-regression appear to show differences between sled loads and possible differences across distances tested. The results of this review and meta-analysis seem to warrant further investigations into the possibility that UDS may be a superior sprint training method to resisted and traditional sprinting.

**Keywords:** performance; resisted sprint; hill sprint.

**Funding:** This research received no external funding.

**References**

1. Alcaraz, P.E.; Carlos-Vivas, J.; Oponjuru, B.O.; Martínez-Rodríguez, A. The effectiveness of resisted sled training (RST) for sprint performance: a systematic review and meta-analysis. *Sports Med* **2018**, 48, 2143–65.
2. Bissas, A., Paradisis, G.P., Nicholson, G., Walker, J., Hanley, B., Havenetidis, K. et al. Development and maintenance of sprint training adaptations: an uphill-downhill study. *J Strength Cond Res* **2022**, 36, 90–8.
3. Fernández-Galván, L.M.; Casado, A.; García-Ramos, A.; Haff, G.G. Effects of vest and sled resisted sprint training on sprint performance in young soccer players: a systematic review and meta- analysis. *J Strength Cond Res* **2022**, 36, 2023–34.
4. Rumpf, M.C.; Lockie, R.G.; Cronin, J.B.; Jalilvand F. Effect of different sprint training methods on sprint performance over various distances: a brief review. *J Strength Cond Res* **2016**, 30, 1767–85.

### 2.22. The Relationship between Training Load Variables and Non-Contact Injury Incidences in Soccer Players: A Systematic Review

Bartu İnal ^1^, Tomás Freitas ^1,2,3,^* and Konstantinos Spyrou ^1,2,3^

^1^ UCAM Research Center for High Performance Sport, UCAM Universidad Católica de Murcia, Murcia Spain; bartuinalscifoot1@gmail.com; tfreitas@ucam.edu; kspyrou@ucam.edu

^2^ Facultad de Deporte UCAM Universidad Católica de Murcia, Murcia Spain

^3^ Strength and Conditioning Society, Murcia, Spain

* Correspondence: tfreitas@ucam.edu

**Abstract:** Muscle injuries comprise of 31% of the total injuries in soccer (1) and decreased injury occurrences in the squad is related with a higher ranking in the league (2). Thus, monitoring training load has been an essential concept in the soccer environment to prevent players from potential non-contact injury incidences. However, selecting sensitive workload variables and indices to detect injury risk is a complex process for practitioners and sport scientists. This systematic review aimed to investigate the relationship between training load variables and non-contact injury incidences in soccer players. The studies were retrieved through three databases: SportDISCUS, PubMed and Web of Science. The search was limited to studies published prior to May 2022 and Newcastle-Ottawa Scale (NOS) was used to assess the methodological quality of the included studies. A total of the 18 included studies, 13 showed significant relationship between workload variables and non-contact injury incidences in soccer players. The use of sRPE (session-rating of perceived exertion) combined with ACWR (acute:chronic workload ratio) and weekly cumulative load was correlated with non-contact injury incidences while GPS-derived load variables were shown to have a conflicting evidence to be utilized through injury prevention purpose in practical soccer environment.

**Keywords:** football; training load; injury risk; monitoring.

**Funding:** This research received no external funding.

**References**

1. Ekstrand, J.; Hägglund, M.; Waldén, M. Epidemiology of muscle injuries in professional football (soccer). *Am J Sports Med* **2011**, *39*, 1226–32.
2. Eirale, C.; Tol, J.L.; Farooq, A.; Smiley, F.; Chalabi, H. Low injury rate strongly correlates with team success in Qatari professional football. *Br J Sports Med* **2013**, *47*, 807–8.

### 2.23. Effect of Training on Different Surfaces in Physical Performance Measures in Youth Tennis Players

**Kyr** **iaki Kakamouka ^1^ Katerina Tsoulfa ^1^ Dalamitros Athanasios ^2^ and Vasiliki Manou ^2^,** *

^1^ School of Physical Education & Sport Sciences, Aristotle University of Thessaloniki, Thessaloniki, Greece; kikikak3005@gmail.com; tsoulfaa@gmail.com

^2^ Laboratory of Evaluation of Human Biological Performance, School of Physical Education & Sport Sciences, Aristotle University of Thessaloniki, Thessaloniki, Greece; dalammi@phed.auth.gr; vmanou@phed.auth.gr

* Correspondence: vmanou@phed.auth.gr

**Abstract:** Tennis is a high-intensity intermittent sport characterized by multidirectional movements, while performance is influenced by the playing surface. This study aimed to compare performance in physical measures during training on different court surfaces in young tennis players. Twenty-nine male and female tennis players from different tennis clubs volunteered to participate in this study. Fourteen participants trained mainly in a hard court surface (Group A; mean ± SD: chronological age 13.96 ± 1.53 years, height 165.79 ± 15.37 cm, body mass 57.16 ± 13.77 kg, Tanner stage 3.07 ± 1.07, PHV 0.61 ± 1.57 years), while 15 had clay court as their basic training surface (Group B; mean ± SD: chronological age 12.61 ± 1.38 years, height 156.50 ± 13.53 cm, body mass 45.21 ± 12.82 kg, Tanner stage 2.00 ± 0.85, PHV −0.98 ± 1.63 years). The physical performance measures included acceleration (5 m), running speed (20 m), vertical jumping ability (CMJ), change of direction (Spider test), agility (505 test), and aerobic endurance (Hit & Run test). Both groups were evaluated in both court surfaces. According to the results, the performance of Group A was higher during all field tests on the respective training court surface (hard court) (*p* ≤ 0.01; 0.05), while Group B showed higher performance in the clay court only during the Hit and Turn test (*p* ≤ 0.05). In addition, both groups showed enhanced performance in all tests when evaluated on hard court (*p* ≤ 0.05), compared to clay court, with the exception of Hit and Run test where no differences were observed. The main training surface can affect physical performance measures and should be considered by coaches when organizing physical conditioning programs or during the performance assessment for young tennis players.

**Keywords:** court surface; physical fitness; tennis.

**Funding:** This research received no external funding.

**References**

1. Fleming, J.A.; Field, A. The demands of training and match-play on elite and highly trained junior tennis players: A systematic review. *Int J Sports Sci Coach* **2023**, *18*, 1365–1376.
2. Girard O.; Eicher F. Effects of the playing surface on plantar pressures and potential injuries in tennis. *Br J Sports Med* **2007**, *41*, 733–738.
3. Pluim, B.M.; Jansen, M.G.T. Physical Demands of Tennis Across the Different Court Surfaces, Performance Levels and Sexes: A Systematic Review with Meta-analysis. *Sports Med* **2023**, *53*, 807–836.
4. Xiao W.; Geok SK; Effect of Exercise Training on Physical Fitness Among Young Tennis Players: A Systematic Review. *Front Public Health* **2022,** *10*, 843021.

### 2.24. Physiological Responses to Repeated Maximum-Intensity Efforts in Finswimming

Gregory Kalaitzoglidis ^1,^*, Ioannis D. Kostoulas ^2^, Argyris G. Toubekis ^3^, Konstantina Karatrantou ^4^ and Vassilis Gerodimos ^4^

^1^ Department of Medicine, University of Thessaly

^2^ Department of Physical & Cultural Education, Hellenic Army Academy

^3^ Department of Physical Education & Sport Science, National & Kapodistrian University of Athens

^4^ Department of Physical Education & Sport Science, University of Thessaly

* Correspondence: gkalaitzoglidis@gmail.com; Tel.: 00306973070602

**Abstract:** Finswimming (FS) athletes often train by swimming short distances under apnoea conditions, which results in the gradual development of arterial hypoxaemia and hypercapnia, but also in the manifestation of the diving reflex (1). The purpose of this study was to compare physiological responses to repeated maximum-intensity efforts under two different finswimming conditions: surface finswimming and apnoea finswimming. The participants were elite finswimming athletes (n = 13, age: 17.3 ± 2.7 years, body mass: 70.6 ± 16.4 kg, height: 176 ± 7 cm). They performed four 50-m swimming maximum-intensity efforts with monofin (4 × 50 m) under two different conditions: (i) on the surface (S), with a snorkel, and (ii) underwater (U) (total apnoea). Recovery time between each effort was 2 min (2). The swimming time was recorded for each effort. Heart rate (HR) was recorded continuously in both conditions. Performance time in the U condition was better than in the S condition (U:17.73 ± 1.18 vs. S:19.94 ± 1.41 s, *p* < 0.01). Mean heart rate during four 50-m was decreased in U compared to S as it was also observed in peak heart rate of each 50-m sprint (U: range 167–177 vs. S: range 183–185 b/min, *p* < 0.05). Interestingly, heart rate during U showed a progressive decrease during the last 10 s of each sprint and maintained at lower level during 20 s of recovery compared to S session (U: range 141–151 vs. S: range 166–174 b/min, *p* < 0.05). Heart rate subsequently increased to a second peak within the first 30 s of recovery in U to reach values similar to S session. Upon comparison of the two conditions, we conclude that FS athletes moved with a higher velocity underwater (3). This observation can be explained by the fact that underwater swimming involves less hydrodynamic drag. Moreover, heart rate was decreased because of the apnoea activated diving reflex (4).

**Keywords:** arterial hypoxia; hypercapnia; diving reflex; apnoea.

**Funding:** This research received no external funding.

**References**

1. Vašíčková, J.; Neumannová, K.; Svozil, Z. The effect of respiratory muscle training on fin-swimmers’ performance. *J Sports Sci Med* **2017**, *16*, 521.
2. Schagatay, E.; Kampen, M.; Andersson, J. Effects of repeated apneas on apneic time and diving response in non-divers. *Undersea Hyperb Med* **1999**, *26*, 143–149.
3. Nicolas, G.; Bideau, B. A kinematic and dynamic comparison of surface and underwater displacement in high level monofin swimming. *Hum Mov Sci* **2009**, *28*, 480–493. doi:10.1016/j.humov.2009.02.004.
4. Andersson, J.P.A.; Linér, M.H.; Rünow, E.; Schagatay, E.K.A. Diving response and arterial oxygen saturation during apnea and exercise in breath-hold divers. *J Appl Physiol* **2002**, *93*, 882–886.

### 2.25. Effect of a Balance and Multi-Directional Plyometric Training on Vertical Jumping and Lower-Limb Explosive Performance in Young Male and Female Soccer Players

Evangelos Kanioris ^1^ and Maria-Elissavet Nikolaidou ^2,^*

^1^ School of Physical Education and Sport Science, National and Kapodistrian University of Athens; vagelispre@gmail.com

^2^ School of Physical Education and Sport Science, National and Kapodistrian University of Athens; mnikola@phed.uoa.gr

* Correspondence: mnikola@phed.uoa.gr; Tel.: +302107276182

**Abstract:** Multi-directional plyometric training (MPT) has been shown to optimize training adaptations in youth soccer (1,2), however studies conducted in female athletes are limited (3). This study assessed the effect of a combined balance and MPT on jumping and explosive performance in young male and female soccer players. Twenty (10 male, 10 female, age: 12.6 ± 1.6 years, body mass: 47.1 ± 10.8 kg) under-13 (U-13) young soccer players with 4.5 ± 2.7 years of training experience followed a 6-week balance and MPT (2 sessions/week, 20 min/session) consisting of static and dynamic balance and stabilization as well as multi-directional plyometric exercises applied at progressively increasing difficulty with regards to base of support, visual information, speed of movement. Training sessions were carried out before the typical soccer training, following 5–7 min of standardized warm-up and 48-h apart. Before (PRE) and after (POST) training, the vertical ground reaction force was recorded by means of a custom-made uniaxial force platform (Wii, 1000 Hz, Biovision) to evaluate vertical jumping and lower-limb explosive performance by measurements of countermovement (CMJ) and squat jumps (SJ) with no arm swing (3 trials/jump). Off-line, the best trial per jump was selected and performance was determined by the parameters of push-off duration (ms), jump height (cm), maximum force (N), maximum power (watt) and maximum impulse (N·s^−1^). Two-way (training x sex) repeated-measures ANOVA with Bonferroni post-hoc comparisons were applied to compare anthropometric, vertical jumping (VJP) and explosive performance parameters. The Cohen’s d was used to calculate the magnitude of change (calculated as (POST-PRE/PRE)*100%)) between male and female players. Significant (*p* < 0.01) differences in age, body mass and height were found PRE and POST training regardless of sex. Neither training nor sex had a significant (*p* > 0.05) main effect on any of the VJP and explosive parameters either in CMJ or SJ. No significant (*p* > 0.05) training by sex interaction was found in any examined parameter either in CMJ or SJ. In the CMJ, small to large ES (d = 0.27–0.85) across nearly all parameters and small to medium ES (d = 0.20–0.68) in SJ respectively suggested that the magnitude change was higher for the young female than their male counterparts. Conclusively, the 6-week balance and multi-directional plyometric training program was not effective to improve jumping and explosive performance of U-13 soccer players, however the differentiated magnitude of training between male and female players suggests that gender may be considered as possible mediator when designing training interventions.

**Keywords:** multi-directional plyometric training; balance; soccer; preadolescence.

**Funding:** This research received no external funding.

**References**

1. Ramirez-Campillo, R.; Alvarez, C.; Garcia-Hermoso, A.; Ramirez-Velez, R.; Gentil, P.; Asadi, A.; et al. Effect of vertical, horizontal, and combined plyometric training on explosive, balance, and endurance performance of young soccer players. *J Strength Cond Res* **2015**, *29*, 1784–1795.
2. Jlid, M.C.; Coquart, J.; Maffulli, N.; Paillard, T.; Bisciotti, G.N.; Chamari, K. Effects of in Season Multi-Directional Plyometric Training on Vertical Jump Performance, Change of Direction Speed and Dynamic Postural Control in U-21 Soccer Players. *Front Physiol* **2020**, *11*, 374.
3. Ramirez-Campillo, R.; Alvarez, C.; Garcia-Hermoso, A.; Ramirez-Velez, R.; Gentil, P.; Asadi, A.; et al. Methodological characteristics and future directions for plyometric jump training research: a scoping review. *Sports Med* **2018**, *48*, 1059–1081.

### 2.26. The Specificity of Regulated Blood Circulation in the Limbs during Resistance Training

Marek Kokinda ^1,^*, Tomáš Kozák ^1^, Pavel Ružbarský ^1^, Bibiana Vadašová and Michal Fečík ^2^

^1^ Faculty of Sports, University of Presov, Presov, Slovakia; marek.kokinda@unipo.sk

^2^ Musculoskeletal and Sports Medicine Clinic, Pavol Jozef Šafárik University, Košice, Slovakia; michal.fecik@nke.agel.sk

* Correspondence: marek.kokinda@unipo.sk

**Abstract:** The purpose of this study was to elucidate the effect of resistance training with regulated blood circulation in the limbs on changes in body weight (BW), body fat mass (BFM), skeletal muscle mass (SMM) and muscle strength. Six males aged 25 ± 2.6 years, who are studying at the Faculty of Sport and can be classified as strength athletes, participated in the study. The experimental period lasted 10 weeks and was divided into measuring changes with a pretest, an 8-week intervention period and a posttest. The research methodology was divided into two stages with an emphasis on body composition and muscle strength. The parameters measured and assessed included body composition using the direct segmental multi-frequency bioelectrical impedance analysis which was complemented by the measurement of circumferential characteristics with emphasis on the biceps in elbow flexion, quadriceps femoris in knee extension and chest in inspiration. Muscle strength was assessed by maximal hand grip dynamometry, flexed-arm hang, front squat with barbell and bench press with emphasis on speed in the concentric phase of the movement for two repetitions. The applied intervention program was implemented 3 times per week for 40 min and consisted of 8 exercises performed in a horizontal sequence. The loading intensity was at 20% of the 1RPM with increasing the cuff pressure by 20 SKUs each week. The parameters were assessed by the Wilcoxon signed rank test. The results showed that there are no changes in somatic parameters BW, BFM, SMM and included circumference characteristics by the applied intervention. Concerning muscle strength, there was a significant improvement (*p* < 0.05) in power in the front squat by 8 kg and 75.5 W with a 0.02 m/s increase in velocity during the concentric phase of the movement. On the other hand, there was a deterioration (*p* < 0.05) in flexed-arm hang by 7.5 s. Based on these results, the periodization of explosive strength development can be modified.

**Keywords:** strength abilities; kaatsu; combat sports; safety; health.

**Funding:** This research was funded by the Scientific Grant Agency of the Ministry of Education, Science, Research and Sport of the Slovak Republic and Slovak Academy of Sciences, grant number VEGA 1/0544/23 entitled *Optimalization of muscle strength and hypertrophy by training with blood flow restriction in the limbs in combat athletes*.

**References**

1. Cook, C.J. et al. Improving strength and power in trained athletes with 3 weeks of occlusion training. *Int J Sports Physiol Perform* **2014**, *9*, 166–172.
2. Miller, B.C. et al. The Systemic Effects of Blood Flow Restriction Training: A Systematic Review. *Int J Sports Phys Ther* **2021,** *16,* 978–990.
3. Scott, B.R. et al. Exercise with Blood Flow Restriction: An Update Evidence-Based Approach for Enhanced Muscular Development. *Sports Med* **2015**, *45*, 313–325.
4. Takada, S. et al. Blood flow restriction exercise in sprinters and endurance runners. *Med Sci Sports Exerc* **2012**, *44*, 413–419.

### 2.27. Evaluation of the Force-Angle Relationship of the Knee Joint Using Different Measurement Systems

Christina Kosti ^1,^*, Athanasios Tsoukos ^1^, Iakovos Pelekis ^2^, Vassilis Paschalis ^1^, Gerasimos Terzis ^1^ and Gregory Bogdanis ^1^

^1^ School of Physical Education and Sports Science, National and Kapodistrian University of Athens, Greece; chkosti@phed.uoa.gr

^2^ School of Physical Education and Sports Science, Democritus University of Thrace, Greece

* Correspondence: chkosti@phed.uoa.gr

**Abstract:** This study compared knee flexor and extensor strength using two muscle strength assessment systems: isokinetic dynamometry (ISD) and isometric handheld dynamometry (HHD). The aim was to assess the feasibility of using low-cost measurement tools, such as HHD, to evaluate muscle strength across the entire range of motion (ROM) of the knee joint, as an alternative to ISD, which is considered as the “gold” standard. Twelve healthy athletes (7 men, 5 women) aged 24 ± 2 years performed three maximal concentric flexion-extension efforts on the ISD at and angular velocity of 60°/s and two isometric efforts at various angles (10°, 30°, 50°, 70°, 90°, 110°) in the prone and seated positions. In a separate session, the corresponding isometric efforts were evaluated using the HHD in the prone position. The knee angle vs. force relationships were modeled using third order polynomials (r^2^ = 0.995), and comparisons of the peak force at each knee angle, as well as the calculated hamstring-to-quadriceps (H/Q) ratios for each knee angle were made between the measurement systems. The HHD measurements for knee extension did not significantly differ from the isometric ISD results in the prone position. Also, the HHD measurements for knee flexion were similar with the ISD results in the seated position (*p* > 0.05). H/Q ratios calculated for each knee angle ranged from 0.2 to 4.7 and were different between angles (*p* < 0.001). However, when calculated conventionally (i.e., peak flexion divided by peak extension strength) were similar between HHD and ISD-seated positions (50–55%). The wide range of H/Q ratios between angles was attributed to variations in the force-length characteristics of the rectus femoris (1) and hamstring muscles (2), due to the different hip angle depending on the evaluation position (prone or seated) (3). This study demonstrated that HHD may be a practical and affordable tool for sports professionals to conduct fast, accurate, and reliable strength measurements on athletes, emphasizing the importance of assessing a joint’s entire ROM, so as to quantify the force-angle relationship of the knee joint according to each sport’s specific demands.

**Keywords:** handheld dynamometry; isokinetic dynamometry; H/Q ratio; angle-specific force.

**Funding:** This research received no external funding.

**References**

1. Ema, R.; Wakahara, T.; Kawakami, Y. Effect of hip joint angle on concentric knee extension torque. *J Electromyogr Kinesiol* **2017**, *37*, 141–146.
2. Kellis, E.; Blazevich, A. J. Hamstrings force-length relationships and their implications for angle-specific joint torques: a narrative review. *BMC Sports Sci Med Rehabil* **2022**, *14*, 166.
3. Baumgart, C.; Kurz, E.; Freiwald, J.; Hoppe, M.W. Effects of Hip Flexion on Knee Extension and Flexion Isokinetic Angle-Specific Torques and HQ-Ratios. *Sport Med—Open* **2021**, *7*.

### 2.28. The Conjoined Impact of Balance and External Load on Marks-Manship Ability

Ioannis Kostoulas ^1,^*, Anastasios Karagiannis ^1^ Konstantinos Havenetidis ^1^ and Stylianos Kounalakis ^1^

^1^ Hellenic Army Academy, Faculty of Physical and Cultural Education; jkost@otenet.gr

* Correspondence: jkost@otenet.gr

**Abstract:** The marksmanship ability of biathletes and military personnel is affected by postural sway, which is influenced by both balance and external load (1, 2). Nonetheless, the impact of their synergy on marksmanship ability remains uncharted territory. The aim of our study was to examine the combined impact of balance disruptions and increased load on marksmanship ability. The research involved 17 males and 1 female officer cadets from the Hellenic Army Academy, who aged 18 ± 3 years old. Their body mass was 75 ± 5 kg and their stature was 178 ± 7 cm. The rifle shooting test involved firing 30 shots while standing, divided into three sets of 10 shots each. The test was conducted twice on different days. All assessments performed in a shooting simulator across four circumstances: one on a stable (onB) and another on an unstable (offB) surface, and with subjects attired in either military-inspired gear and equipment (L—mean weight of 22.5 ± 1.7 kg) or athletic clothing (noL). The variables under analysis were the score and the % center of gravity (COG) of shots in the proximity of a designated point. Although balance and load had individually adverse effects on both shooting score and COG, their combined impact was more substantial. Specifically, the score was significantly lower for offB-L in comparison to all other conditions [63 ± 14, 55 ± 13, 56 ± 14 and 46 ± 14 for onB-noL, offB-noL onB-L and offB-L, respectively; F(1,17): 15, *p* < 0.01, η^2^ = 0.60]. Likewise, offB-L displayed a lower COG compared to the other three conditions [77 ± 10, 66 ± 10, 72 ± 11 and 59 ± 13 for onB-noL, offB-noL onB-L and offB-L, respectively; F(1,17): 15, *p* < 0.00, η^2^ = 0.76]. By causing a disturbance in postural sway, balance and external load were seen to have a negative impact on shooting accuracy. The combined effect of these factors was found to exacerbate the decline in performance. Consequently, training programs aimed at improving shooting ability should emphasize a comprehensive approach that includes balance and core stability exercises with external load, rather than exclusively focusing on isolated training of these elements.

**Keywords:** load carriage; military activities; simulated shooting.

**Funding:** This research received no external funding.

**References**

1. Hung, M.H.; Lin, K.C.; Wu, C.C.; Juang, J.H.; Lin, Y.Y.; Chang, C.Y. Effects of Complex Functional Strength Training on Balance and Shooting Performance of Rifle Shooters. *Appl Sci* **2021**, *11*, 6143. doi:10.3390/ app11136143.
2. Heller, M.F.; Challis, J.H.; Sharkey, N.A. Changes in postural sway as a consequence of wearing a military backpack. *Gait Posture* **2009**, *30,* 115–7. doi:10.1016/j.gaitpost.2009.02.015.

### 2.29. Association between Baseline Interleukin 6 Levels and Responsiveness to Resistance Training in Hemodialysis Patients

Lorena Lopes ^1,^*, João Felipe Mota ^2^ and Maria do Rosário Gondim Peixoto ^2^

^1^ Centro Universitário de Mineiros, Goiás, Brazil; lorena.lopes @unifimes.edu.br

^2^ School of Nutrition, Federal University of Goias, Brazil. jfemota@gmail.com; maria_rosario_gondim@ufg.br

* Correspondence: lorena.lopes @unifimes.edu.br

**Abstract:** Hemodialysis (HD) patients often experience a decline in both muscle mass and function, a phenomenon that can be linked to elevated levels of pro-inflammatory proteins, particularly interleukin 6 (IL-6). Nevertheless, the resistance training (RT) emerges as a promising strategy not only to enhance muscle function and preserve overall functional capacity but also to potentially improve the inflammatory profile in these patients (1, 2). It’s important to note, however, that the responses to RT can vary among individuals due to several individual factors. Thus, the aim of this study is to identify if the gain in muscle mass after 12 weeks of RT is associated with inflammatory markers levels. Methods: Fifty HD patients (51% of males, aged 30–75 years old), undergoing HD treatment for at least three months, were enrolled in a 12-week intradialytic RT program per-formed three times per week. The training groups consisted of a high-load intradialytic group (HLG n = 14, 8–10 repetitions), moderate-load intradialytic group (MLG n = 16, 16–18 repetitions), and control group (CG n = 20, stretching exercise). The total training volume was equalized among training groups. Results: The HLG had superior gains in lean leg mass compared to the CG (*p* = 0.04; +2.84%; effect size = 0.92). There were no differences in serum concentration of IL-6, IL-10 and tumour necrosis factor α (TNFα) any group after the RT period. However, when analyzing all individuals who trained, it was noted that those who responded to the training had 59.2% lower baseline serum concentration of IL-6 compared to non-responders (1.6 ± 0.9 pg/mL versus 2.7± 2.4 pg/mL, *p* = 0.041). Moreover, there was an inverse correlation between changes in muscle mass and baseline concentra-tion of IL-6 (r= −0.31 *p* = 0.036). Among the subjects who performed the RT, 73% were considered responders, with 4 subjects each in the HLG and MLG groups being considered non-responders (28.5% versus 22.2%). This interesting data could be related to the role of IL-6 in muscle mass (3). Studies in health subjects have shown that the infusion of IL-6 causes an increase in net muscle protein breakdown and ~50% reduction in total muscle protein turnover (4). Conclusion: Inflammatory conditions impair the ability of the muscle to respond to RT

**Keywords:** resistance training; hemodialysis; inflammation.

**Funding:** This research received no external funding.

**References**

1. Kirkman, D.L.; Mullins, P.; Junglee, N.A.; Kumwenda, M.; Jibani, M.M.; Macdonald, J.H. Anabolic exercise in haemodialysis patients: A randomised controlled pilot study. *J Cachexia Sarcopenia Muscle* **2014**, *5*, 199–207. doi:10.1007/s13539-014-0140-3.
2. Cheema, B.S.B. Review article: Tackling the survival issue in end-stage renal disease: Time to get physical on haemodialysis. *Nephrology* **2008**, *13*, 560–9. doi:10.1111/j.1440-1797.2008.01036.x.
3. Muñoz-Cánoves, P.; Scheele, C.; Pedersen, B.K.; Serrano, A.L. Interleukin-6 myokine signaling in skeletal muscle: A double-edged sword? *FEBS J* **2013**, *280*, 4131–48. doi:10.1111/febs.12338.
4. Van Hall, G.; Steensberg, A.; Sacchetti, M.; Fischer, C.; Keller, C.; Schjerling, P.; et al. Interleukin-6 stimulates lipolysis and fat oxidation in humans. *J Clin Endocrinol Metab* **2003**, *88*, 3005–10. https://doi.org/10.1210/jc.2002-021687.

### 2.30. Jump Height or Reactive Strength Index: Which Is a Better Indicator of Neuromuscular Readiness in Youth Basketball Players?

Elena Marín-Cascales ^1,2,3,^*, Riccardo Guerrieri ^1^, Konstantinos Spyrou ^1,2,3^ and Tomás T. Freitas ^1,2,3,4^

^1^ UCAM Research Center for High Performance Sport, UCAM Universidad Católica de Murcia, Murcia, Spain; emarin@ucam.edu; rguerrieri@alu.ucam.edu; kspyrou@ucam.edu; tfreitas@ucam.edu

^2^ Facultad de Deporte, UCAM Universidad Católica de Murcia, Murcia, Spain

^3^ Strength and Conditioning Society, Murcia, Spain

^4^ NAR-Nucleus of High Performance in Sport, São Paulo, Brazil

^*^ Correspondence: emarin@ucam.edu

**Abstract:** Basketball is an intermittent team-sport characterized by repeated high-intensity efforts (e.g., sprints or jumps) that put a high demand on the neuromuscular system (1). Thus, during the season, practitioners frequently assess neuromuscular function and readiness (2). The countermovement jump (CMJ) is one of the most utilized tests, and jump height is the most reported metric (1). However, previous studies have argued that: (i) CMJ height may not accurately portrait neuromuscular readiness as players may modify movement strategy to achieve similar jump performances (3); and (ii) in basketball, fast stretch-shortening cycle actions are predominant and these may not be properly assessed with the CMJ (2). Therefore, the repeated jump test (RPJ) has been recently proposed as an alternative but it remains unknown whether variables like the reactive strength index (RSI) are actually better indicators of neuromuscular readiness. The aim of this study was to compare the acute changes in RPJ RSI and CMJ height to determine which metric is a better indicator of neuromuscular readiness in young basketball players. Seventeen players (10 females, age = 16.3 ± 1.3 years, height = 172.4 ± 7.4 cm, body mass = 63.9 ± 7.0 kg and 7 males, age = 15.0 ± 1.1 years, height = 188.7 ± 4.9 cm, body mass = 75.5 ± 6.8 kg) from an elite basketball academy volunteered to participate. Twice per week, during a one-month off-season training camp, players completed 3 CMJs and a RPJ test (consisting of 5 reactive jumps) on a force platform before and after a standardized warm-up (performed prior to a resistance training session). The best CMJ performance and the average of the 3 best reactive jumps were kept for analysis. A linear mixed model was used to examine the differences in jumping variables pre- and post-warm-up accounting for individual repeated measures. A significant interaction was found for RPJ RSI (*p* = 0.012) but not for CMJ height (*p* = 0.143). Specifically, the former variable increased from pre- to post-warm-up whereas the latter did not significantly change. The results suggest that RSI was more sensible than CMJ height to assess neuromuscular readiness, most likely because, during the CMJ, players may have modified their jumping strategy (e.g., different countermovement depth or movement velocity) to achieve similar outputs. In conclusion, practitioners should consider utilizing the RSI to obtain more accurate information about young basketball players neuromuscular priming and readiness instead of CMJ height.

**Keywords:** team-sport; countermovement jump; CMJ; priming; performance; testing.

**Funding:** This research received no external funding.

**References**

1. Petway, A.J., Freitas, T.T., Calleja-González, J., Medina Leal, D., Alcaraz, P.E. Training load and match-play demands in basketball based on competition level: A systematic review. *PloS one* **2020**, *15*, e0229212.
2. Petway, A.J., Freitas, T.T., Calleja-González, J., Alcaraz, P.E. Match day-1 reactive strength index and in-game peak speed in collegiate division I basketball. *Int J Environ Res Public Health* **2021**, *18*, 3259.
3. Bishop, C., Turner, A., Jordan, M., Harry, J., Loturco, I., Lake, J., Comfort, P. A framework to guide practitioners for selecting metrics during the countermovement and drop jump tests. *Strength Cond J* **2022**, *44*, 95–103.

### 2.31. Influence of Human Knee Flexors Contraction Intensity on Ham-String Load-Sharing Response during an Endurance Task until Exhaustion

Antonio Martínez-Serrano ^1,3,4,^*, Régis Radaelli ^2^, Tomás T. Freitas ^1,3,4,5^, Pedro E. Alcaraz ^1,3,4^ and Sandro R. Freitas ^6^

^1^ UCAM Research Center for High Performance Sport, Catholic University of Murcia, Murcia, Spain; amartinez30@ucam.edu, tfreitas@ucam.edu, palcaraz@ucam.edu

^2^ Centro de Investigação Interdisciplinar Egas Moniz; (CiiEM), Egas Moniz-Cooperativa de Ensino Superior, Monte da Caparica, Portugal; rradaelli@egasmoniz.edu.pt

^3^ Strength and Conditioning Society, Murcia, Spain

^4^ Faculty of Sport Sciences, Catholic University of Murcia, Murcia, Spain

^5^ NAR—Nucleus of High Performance in Sport, São Paulo, Brazil

^6^ Laboratório de Função Neuromuscular, Faculdade de Motricidade Humana, Universidade de Lisboa, Estrada da Costa, Cruz Quebrada, Dafundo, Lisboa, Portugal; pt.sandrofreitas@gmail.com

* Correspondence: amartinez30@ucam.edu; Tel.: +34-968-27-85-66

**Abstract:** The mechanical characteristics of muscles have been studied using ultrasound-based shear wave elastography (SWE), revealing information on muscle stiffness and load distribution (1–3). However, the active stiffness of biceps femoris long head (BFlh) and semitendinosus (ST) muscles under fatigue conditions at various contraction intensities have not been explored. This study aimed to compare the effects of knee flexor’s isometric contraction until exhaustion performed at 20% vs. 40% of maximal voluntary isometric contraction (MVIC), on the active stiffness responses of BFlh and ST. Eighteen recreationally active males performed two experimental sessions. The knee flexors’ MVIC was assessed before the fatiguing task, which involved a submaximal isometric contraction until failure at 20% or 40% of MVIC. Mean active stiffness of BFlh and ST for each percentile during the fatigue task was determined (i.e., 10 percentiles were calculated, each corresponding to 10% of the contraction duration). BFlh active stiffness remained unaltered in all experimental conditions (20% MVIC: *p* = 0.190 η2 = 0.087; 40% MVIC: *p* = 0.458 η2 = 0.054), while ST active stiffness decreased from 60% contraction time (*p* = 0.006) in the 20% MVIC session. These results suggest that contraction duration could play a major role to induce changes on hamstrings mechanical properties during fatigue tasks when compared to contraction intensity.

**Keywords:** shear wave elastography; biceps femoris; semitendinosus; mechanical; performance.

**Funding:** This research received no external funding.

**References**

1. Avrillon, S.; Hug, F.; Guilhem, G. Between-muscle differences in coactivation assessed using elastography. *J Electromyogr Kinesiol* **2018**, *43*, 88–94.
2. Evangelidis, P.E.; Shan, X.; Otsuka, S.; Yang, C.; Yamagishi, T.; Kawakami, Y. Hamstrings load bearing in different contraction types and intensities: A shear-wave and B-mode ultrasonographic study. *PLoS One* **2021**, *16*.
3. Mendes, B.; Firmino, T.; Oliveira, R.; Neto, T.; Cruz-Montecinos, C.; Cerda, M.; Correia, J.P.; Vaz, J.R.; Freitas, S.R. Effects of knee flexor submaximal isometric contraction until exhaustion on semitendinosus and biceps femoris long head shear modulus in healthy individuals. *Sci Rep* **2020**, *10*.

### 2.32. Effects of Cueing on Vertical Jump Performance in Professional Volleyball Players

Sebastian Masel ^1,^* and Marcin Maciejczyk ^1^*

^1^ University of Physical Education in Kraków, Poland; sebas-tian.masel@doctoral.awf.krakow.pl; marcin.maciejczyk@awf.krakow.pl

* Correspondence: sebastian.masel@doctoral.awf.krakow.pl; Tel.: +48-665-992-426

**Abstract:** The strength and conditioning specialists seek the optimal ways to control the physical performance of the athletes and to monitor their neuromuscular fatigue (1). One of the frequently implemented tests to measure lower-body explosive power is a countermovement jump (CMJ) (2,3). Various technologies have been introduced to perform the measurements with force platforms being considered as ‚gold standard’ and contact mats being more affordable option (4). In this study 9 professional volleyball players (age: 23.8 ± 1.5 years; body height: 192.9 ± 8.3 cm; body mass: 83.6 ± 7.1 kg) performed with two different cueing approaches 1008 CMJs throughout 2 competitive seasons (S1 and S2)-540 (60 ± 10.4 by each athlete) in S1 and 468 (52 ± 6.2 by each athlete) in S2 respectively. In both seasons the measurements were performed 1 to 2 times per week after the standardized warm-up. The athletes performed 2 attempts of CMJ without arm swing (hands placed on the hips) on the contact mat in the same order with approximately 90s rest period between attempts. In both seasons the athletes could choose their stance width and were instructed to descend quickly until they reach approximately 90 degrees of knee flexion and jump as high as possible. In S1 the athletes were instructed to perform the jump in the same manner every testing session while having more freedom in the movement (i.e., bending the hips during the flight as in the volleyball block). In S2 additional cues included forbidding athletes any movement during the flight and an additional quick rebound jump immediately after landing to ensure soft landing on the mat in the same position as during the take-off (full plantar flexion). The mean jump height (46.7 ± 3.53 cm in S1 and 44.5 ± 3.32 cm in S2) and standard deviation were calculated. Significance of difference was determined by Wilcoxon’s test and effect size (ES) using Cohen’s d. The significant difference between S1 and S2 in jump height (p: 0.01; ES: 0.64) was noted. After introducing additional cueing in S2 mean jump height decreased by 4.6% and standard deviation by 30.4%. The results of the study indicate that the cueing and subsequent execution of the jump may impact vertical jump height using contact mat. Thus, the practitioners should consider introducing more rigorous testing procedures using this type of equipment in order to obtain more repeatable measurements and detect the potential neuromuscular fatigue more efficiently.

**Keywords:** power; testing; monitoring; sports performance.

**Funding:** This research received no external funding.

**References**

1. Weakley, J.; Black, G.; Mclaren, S.; Scantlebury, S.; Suchomel, S.J.; McMahon, E.; Watts, D.; Read D. Testing and Profiling Athletes: Recommendations for Test Selection, Implementation, and Maximizing Information. *Strength Cond J* **2023**, *publish ahead of print*. doi:10.1519/SSC.0000000000000784
2. Anicic, Z.; Janicijevic D.; Knezevic, O.M.; Garcia-Ramos, A.; Petrovic, M.R.; Cabarkapa, D.; Mirkov, D.M. Assessment of Countermovement Jump: What Should We report? *Life* **2023**, *13*, 190. doi:10.3390/life13010190
3. Claudino, J.G.; Cronin, J.; Mezêncio, B.; McMaster, D.T.; McGuigan, M.; Tricoli, V.; Amadio, A.C.; Serrao, J.C. The counter-movement jump to monitor neuromuscular status: A meta-analysis. *J Scie Med Sport* **2016**, *20*, 4, 397–402. doi:10.1016/j.jsams.2016.08.011
4. Jiménez, C.A.; Moreno-Doutres, D.; Peña, J. Trends Assessing Neuromuscular Fatigue in Team Sports: A Narrative Review. *Sports* **2022**, *10*, 33. doi:10.3390/sports10030033

### 2.33. Muscle Perfusion and Hemodynamic Responses during Resistance Exercise

Konstantinos Mavridis ^1,^*, Anatoli Petridou ^1^, Athanasios Chatzinikolaou ^2^ and Vassilis Mougios ^1^

^1^ School of Physical Education and Sport Science at Thessaloniki, Laboratory of Evaluation of Human Biological Performance, Aristotle University of Thessaloniki; mavridisk98@gmail.com

^2^ School of Physical Education and Sport Science, Laboratory of Physical Performance, Democritus University of Thrace

* Correspondence: mavridisk98@gmail.com

**Abstract:** Near-infrared spectroscopy (NIRS) is widely employed in the study of muscle oxygenation and perfusion in exercise, with use also in resistance exercise in recent years. Muscle perfusion during resistance exercise depends on training parameters, type of muscle activity, and muscle (1–4). Data separating the eccentric and concentric phases are lacking. We have recently reported that muscle oxygenation undulates during the two phases of resistance exercise (3). The aim of this study was to examine whether this is also true with muscle perfusion. Eighteen young resistance-trained males performed 5 sets of parallel high-bar back squat in a Smith machine, with 15 repetitions each: two warm-up sets [at 14% and 45% of 15-repetition-maximum (15RM)] and three main sets at 100% 15RM. NIRS devices were attached to vastus lateralis (VL), vastus medialis (VM) and rectus femoris (RF). During the protocol, total heme (muscle perfusion index) and heart rate (HR) were continuously monitored. After each set arterial blood pressure were measured. Repeated measures ANOVA was performed for data analysis. The significance level was set at α = 0.05. Total heme kinetics showed a slight decrease during the first seconds of each set, an increase in VL, relative stabilization in VM and decrease in RF during the rest of the set, and a slight decrease in VL or increase in VM and RF during recovery. RF had lower total heme starting and final values than VL and VM. From set to set, the starting value increased, while the final value did not change. Also, there was an interaction of muscle and set in the final value (*p* = 0.027). Importantly, total heme undulated during each repetition, decreasing during the descending phase and increasing during the ascending phase. Starting, final, and mean HR values within each set had an increasing trend in the warm-up sets, while remaining relatively stable in the main sets. Systolic pressure gradually decreased after each set, while diastolic pressure remained constant. In conclusion, quadriceps muscle perfusion during back squats displayed diverse patterns depending on the specific muscle, undulating within each repetition in a way that is in accord with the decreased oxygen demand during the descending phase and the increased oxygen demand during the ascending phase. This highlights the rapid response of the circulatory system according to the needs of the exercising muscles.

**Keywords:** barbell squat; concentric phase; eccentric phase; NIRS; resistance exercise; muscle perfusion; undulation of muscle perfusion.

**Funding:** This research received no external funding.

**References**

1. Baudry, S.; Sarrazin, S.; Duchateau, J. Effects of load magnitude on muscular activity and tissue oxygenation during repeated elbow flexions until failure. *Eur J Appl Physiol* **2013**, *113*, 1895–1904.
2. Formenti, D.; Perpetuini, D.; Iodice, P.; Cardone, D.; Michielon, G.; Scurati, R.; Alberti, G.; Merla, A. Effects of knee extension with different speeds of movement on muscle and cerebral oxygenation. *PeerJ* **2018**, *6*, e5704.
3. Mavridis, K.; Petridou, A.; Mougios, V. Muscle oxygenation undulates during each repetition of resistance exercise. 28th Annual Congress of the European College of Sport Science–Book of Abstracts, **2023**, 129.
4. Muthalib, M.; Lee, H.; Millet, G.Y.; Ferrari, M.; Nosaka, K. Comparison between maximal lengthening and shortening contractions for biceps brachii muscle oxygenation and hemodynamics. *J Appl Physiol* **2010**, *109*, 710–720.

### 2.34. Not All Power Training Volumes Result in Muscle Power in-Crease: The Role of Muscle Damage/Inflammation, Oxidative Stress on Muscle Fiber Composition and Power Performance Adaptations

Spyridon Methenitis ^1,^*, Thomas Mpampoulis ^1^, Stasinaki Angeliki-Nikoletta ^1^, Costantinos Papadopoulos ^2^, George Papadimas ^2^, Eleana Chalari ^3^, Tzortzis Nomikos ^3^ and Gerasimos Terzis ^1^

^1^ Sports Performance Laboratory, School of Physical Education & Sports Science, National and Kapodistrian University of Athens, Greece; smetheni@phed.uoa.gr; thompamp@phed.uoa.gr; agstasin@phed.uoa.gr; gterzis@phed.uoa.gr

^2^ A’ Neurology Clinic, Aiginition Hospital, Medical School, National and Kapodistrian University of Athens, Greece; constantinospapadopoulos@yahoo.com; gkpapad@yahoo.gr

^3^ Department of Nutrition and Dietetics, School of Health Science & Education, Harokopio University of Athens, Greece; e.chalari@gmail.com; tnomikos@hua.gr

* Correspondence: smetheni@phed.uoa.gr; Tel.: +30-210-7276173; Fax: +30-210-7276028

**Abstract:** Divergent data exist about the effect of power training volumes on muscle power adaptations. Increased training volumes, especially when eccentric or plyometric exercises are included in a power training session, could lead to significant muscle damage and oxidative stress, which may compromise the magnitude of adaptations. However, until now, this has not been investigated. The aim of the present study was to examine the effect of power training induced muscle damage/inflammation and oxidative stress on muscle fiber composition and power performance. Twenty-nine females were assigned into three groups and performed for 10 weeks either 3 (LV), 6 (MV) or 9 (HV) sets/session of 4 fast velocity eccentric-only half-squats against 70% of 1RM, followed by 3 maximum countermovement jumps (CMJ) after each set. Before and after the intervention half squat 1RM, CMJ, leg press maximum isometric force (MIF) and rate of force development (RFD), as well as vastus lateralis muscle fiber type composition and cross-sectional areas (CSA), were evaluated. One hour before, 2, 24 and 48 h after the 1st training session, blood samples were collected for the determination of muscle damage/inflammatory (CK, CRP, LDH) and of oxidative stress markers (GPX3, PC) concentrations. Half squat 1RM and MIF were increased in all groups (*p* < 0.05), with the highest increases observed in HV (30.1 ± 9.9%–42.2 ± 7.85% and the lowest in LV group (18.6 ± 10.0%–20.2 ± 8.6%). Εarly RFD increased only after LV and MV, with the largest changes observed after LV (*p* = 0.000; LV: 10.0 3 ± 7.59%–34.76 ± 13.45%, MV: 8.01 ± 4.12–25.39 ± 13.79%, HV: −18.61 ± 7.30%–12.75 ± 2.55%). Significant reductions in type IIx muscle fiber percentages and %CSAs after MV and HV, with concomitant increases in type IIa fibers were found (*p* = 0.001). HV, followed by MV induce significant increases in muscle damage/inflammation and oxidative stress markers after the 1st training session (16.6 ± 10.9%–259.1 ± 111.3%; *p* < 0.01). No significant changes were observed after LV. Significant correlations were observed between the changes of muscle damage/inflammation, oxidative stress markers after the 1st training session, and the training induced adaptations in muscle fiber composition, early RFD and CMJ performance (r: −0.678 to 0.821; *p* < 0.05). These results suggest that relatively large power training volumes result in significant increases in muscle damage/inflammation, oxidative stress, which compromise the magnitude of power training adaptations on power performance, probably because they mediate the reduction of type IIx muscle fibers percentage cross sectional areas.

**Keywords:** rate of force development; eccentric power training; explosive performance; resistance training volume; skeletal muscle morphology.

**Funding:** This research received no external funding.

**References**

1. Cormie, P.; McGuigan, M.R.; Newton, R.U. Developing maximal neuromuscular power: part 2—training considerations for improving maximal power production. *Sports Med* **2011**, *41*, 125–146. doi:10.2165/11538500-000000000-00000.
2. Methenitis, S.; Mpampoulis, T.; Spiliopoulou, P.; Papadimas, G.; Papadopoulos, C.; Chalari, E.; Evangelidou, E.; Stasinaki, A.N.; Nomikos, T.; Terzis, G. Muscle fiber composition, jumping performance and rate of force development adaptations induced by different power training volumes in females. *Appl Physiol Nutr Metab* **2020**, *45*, 996–1006. doi:10.1139/apnm-2019-0786.
3. Zacharia, E.; Spiliopoulou, P.; Methenitis, S.; Stasinaki, A.N.; Zaras, N.; Papadopoulos, C.; Papadimas, G.; Karampatsos, G.; Bogdanis, G.; Terzis, G. Changes in muscle power and muscle morphology with different volumes of fast eccentric half-squats. *Sports* **2019**, *7*, 164. doi:https://doi.org/10.3390/sports7070164.
4. Zaras, N.; Spengos, K.; Methenitis, S.; Papadopoulos, C.; Karampatsos, G.; Georgiadis, G.; Stasinaki, A.N.; Manta, P.; Terzis, G. Effects of Strength vs. Ballistic-Power Training on Throwing Performance. *J Sports Sci Med* **2013**, *12*, 130–137. doi:PMC3761775.

### 2.35. The Effect of Resistance Training and Detraining with Multi-Joint or Single-Joint Exercise on Muscle Strength and Muscle Hypertrophy

Thomas Mpampoulis ^1^,*, Aggeliki Stasinaki and Gerasimos Terzis ^1^

^1^ Sports Performance Laboratory, School of Physical Education & Sports Science, National and Kapodistrian University of Athens, Greece; thompamp@phed.uoa.gr

* Correspondence: thompamp@phed.uoa.gr; Tel.: 6977427089

**Abstract:** Resistance exercise is an effective method to increase muscle strength and mass but these adaptations retract after detraining. The use of a multi-joint or a single-joint resistance exercise to increase muscle strength and mass might result in different loss rates of these adaptations at de-training, however, this issue has not been investigated. The aim of the study was to compare the effect of resistance training using either a multi-joint or a single joint exercise on preserving muscle strength and mass gains after 8 weeks of detraining. Fourteen young moderately trained females (height 173 ± 6 cm, mass 62 ± 10 kg), unacquainted with resistance exercise, followed 8 weeks of unilateral leg strength training. One lower extremity was trained with the leg press (LP) and the opposite lower extremity with the knee extension (KE) for 2 sessions per week, 4 sets X 6 repetitions, 80% maximal strength. After 8 weeks of training, they entered a detraining period of 8 weeks. Unilateral maximum strength (1-RM) in leg press and knee extension as well as quadriceps’ muscle cross sectional area (CSA, via ultrasonography) at 3 different sites of the thigh length (20%, 40%, 60%) were evaluated before training (T1), at the end of systematic training (T2) and at the end of detraining (T3). Unilateral maximum strength (1-RM) in leg press and knee extension increased (36.5 ± 11.5% and 28.3 ± 14.8%, respectively, *p* < 0.05) after training, with no difference between interventions (*p* < 0.05). Quadriceps muscle CSA increased at 20% of thigh length (LP: 10.5 ± 5.1% and KE: 7.5 ± 3%, *p* < 0.05, significant difference between training protocols, *p* < 0.05), at 40% of thigh length (LP: 10.2 ± 3.6% and KE: 8.2 ± 3.4%, *p* < 0.05) and at 60% of thigh length (LP: 11.2 ± 4.5% and KE: 7.1 ± 3.2%, *p* < 0.05, significant difference between training protocols, *p* < 0.05), after systematic training. Maximum strength decreased between T2 and T3 (LP: −11.2 ± 6.3% and KE: −16.8 ± 6.5%, *p* < 0.05, significant difference between training protocols, *p* < 0.05) and quadriceps muscle CSA decreased at 20% of thigh length (LP: −6.2 ± 2.7% and KE: −5.7 ± 3.1%, *p* < 0.05) at 40% of thigh length (LP: −5.9 ± 2.1% and KE: −6.3 ± 3.4%, *p* < 0.05) and at 60% of thigh length (LP: −7.0 ± 2.8% and KE: −4.8 ± 2.1%, *p* < 0.05), with no difference between training protocols. There was no difference in the loss of muscle CSA between intervention, from T1 to T3. These results suggest that LP results in larger increases in quadriceps muscle mass compared to the KE. However, after 8 weeks of detraining a similar decrease in muscle mass is expected after the implementation of either exercise paradigms.

**Keywords:** leg press; knee extension; quadriceps; muscle hypertrophy; muscle atrophy.

**Funding:** This research received no external funding.

### 2.36. Comparison of the Economic Cost of Injuries between Different European Professional Football Leagues

Laura Nieto Torrejón ^1,^* and Pedro E. Alcaraz ^2,3,4^

^1^ Economic Faculty, Catholic University of Murcia, Murcia, Spain; lnieto@ucam.edu

^2^ UCAM Research Center for High Performance Sport, Catholic University of Murcia, Murcia, Spain; palcaraz@ucam.edu

^3^ Strength and Conditioning Society, Murcia, Spain

^4^ Faculty of Sport Sciences, Catholic University of Murcia, Murcia, Spain

* Correspondence: lnieto@ucam.edu; Tel.: +34-968-278-656

**Abstract:** The financial loss due to injury is severe in professional football (1). Nevertheless, when describing the economic impact of injuries in football, a statement by a CEO of a UCL’s club, not based on objectively quantified data, is used (2–3). Therefore, this study aims to determine the number of injuries (muscular, joint and remaining injuries), and their corresponding missed matches in order to analyze their economic impact in Bundesliga, LaLiga and Premier League clubs. For that, a cross-sectional study was carried out for the season 2021/2022, and for all players of all clubs of aforementioned leagues. The economic impact of injuries was estimated taking into consideration player´s availability, as a ratio between missed matches and total matches played by each club, and the mean salary cost per club. The high number of injuries, which vary from 1200 to 1400, implies an annual high cost for professional first division football clubs, specifically €110 M, €181 M, and €265 M in Bundesliga, Laliga and Premier League, respectively. Nevertheless, the differences among the three leagues related to the type of injury and the missed matches are statistically significant. For example, LaLiga seems to managed joint injuries better than the others leagues, but not with muscular ones, which are quite superior. Premier League shows the highest number of missed matches, being this difference statistically significant when compared to Bundesliga. These results highlight the importance of reducing the number of injuries through multidisciplinary teams as well as recovery times and let compare strong and weak points of these three leagues in regard to the analyzed variables.

**Keywords:** soccer; player availability; player cost; missed matches.

**Funding:** This research received no external funding.

**References**

1. Eliakim, E.; Morgulev E.; Lidor R.; et al. Estimation of injury costs: financial damage of English Premier League teams’ underachievement due to injuries. *BMJ Open Sport Exerc Med* **2020**, *6*, e000675.
2. Ekstrand, J. Keeping your top players on the pitch: the key to football medicine at a professional level. *Br J Sports Med* **2014**, *47*, 723.
3. Huygaerts, S.; Cos, F.; Cohen, D.D.; et al. Mechanisms of Hamstring Strain Injury: Interactions between Fatigue, Muscle Activation and Function. Sports **2020**, *8*, 65.

### 2.37. Effect of a Single Bout of Foam Rolling Exercise on Postural Balance Performance in Young Female Gymnasts

Maria-Elissavet Nikolaidou* and Leda-Ourania Korda

School of Physical Education and Sport Science, National and Kapodistrian University of Athens; mnikola@phed.uoa.gr; leda.kordas@gmail.com

* Correspondence: mnikola@phed.uoa.gr

**Abstract:** Foam rolling (FR) is acknowledged as an effective method substituting for an intense warm-up to increase the joint range of motion with some studies reporting small balance performance improvements in recreationally active young subjects (1, 2). This study examined the effect of a single bout of FR exercise on postural balance performance in female rhythmic gymnastics athletes. Seven female athletes (age: 22.8 ± 2.2 years, body mass: 61 ± 20 kg) with training experience >10 years and training frequency 4 days/week × 2 h, participated with a counterbalanced randomized order in 3 experimental conditions: (a) FR without vibration (NoVibr) (Live up™, 13 × 38 cm, 650 g), (b) FR with vibration (Vibr) (25 Hz, Pulseroll™, 15 × 38 cm, 1.100 g) and (c) control condition (resting in a comfortable sitting position). The NoVibr and Vibr FR sessions consisted of 3 sets of 30 sec FR bilaterally on the knee flexors—extensors and plantar flexors with 14 min total duration and 15 s rest between set series and muscle groups. FR pressure was applied parallel to the muscle fibers’ orientation at 40 beats/min controlled by a metronome and up to the athletes’ discomfort threshold. Before (PRE) and after (POST) the experimental conditions, the vertical ground reaction force was recorded by a custom-made uni-axial force platform (Wii, 1.000 Hz, Biovision™) during quiet barefooted stance trials of: (a) static two-legged, and (b) static one-legged (left-right) postural conditions (open eyes, 30 sec of 3 trials/condition). Off-line, the center of pressure (CoP) data were calculated (Δt analysis = 20 sec, average of 3 trials) and postural balance performance was assessed by: (a) CoP path length (PL, cm), and (b) CoP sway amplitude (cm) in the anteroposterior (AP_sway_) and mediolateral (ML_sway_) directions. Two-way repeated-measures ANOVA (condition x measure) with Bonferroni post-hoc comparisons were applied (a = 0.05). There was no significant (*p* > 0.05) main effect of condition either in two-legged (% change, PL: −4 ± 14, AP_sway_: 0.2 ± 33, ML_sway_: 17 ± 32) or one-legged (% change, Left: PL: −12 ± 13, AP_sway_: 6 ± 33, ML_sway_: −8 ± 20; Right: PL: 2 ± 19, AP_sway_: 2 ± 29, ML_sway_: 15 ± 28) stance in any CoP parameter. Similarly, no significant (*p* > 0.05) effect was found between PRE and POST measures and no significant (*p* > 0.05) condition by measure interaction was found in any postural condition. Conclusively, in a highly demanding for fine postural control sport such as rhythmic gymnastics (3), a single FR session with or without vibration does not appear to be a sufficient stimulus to invoke positive changes in postural balance.

**Keywords:** myofascial release; foam rolling; vibration; postural balance; gymnastics.

**Funding:** This research received no external funding.

**References**

1. Zhang, Q.; Trama, R.; Fouré, A.; Hautier, C.A. The immediate effects of self-myofacial release on flexibility, jump performance and dynamic balance ability. *J Hum Kinet* **2020**, *75*, 139–148.
2. Chia-Lun, L.; I-Hua, C.; Bo-Jhang, L.; Wen-Dien, C.; Nai-Jen, C. Comparison of vibration rolling, nonvibration rolling, and static stretching as a warm-up exercise on flexibility, joint proprioception, muscle strength, and balance in young adults. *J Sports Sci* **2018**, *36*, 2575–2582.
3. Vuillerme, N.; Teasdale, N.; Nougier, V. The effect of expertise in gymnastics on proprioceptive sensory integration in human subjects. *Neurosci Letters* **2001**, *311*, 73–76.

### 2.38. The Impact of Mental Fatigue on Artistic Swimmers’ Physiological Responses Following High-Intensity Choreography

Stavroula Ntomali ^1,^*, Argyris Toubekis ^1^ and Petros Botonis ^1^

^1^ School of Physical Education and Sports Science, National and Kapodistrian University of Athens, Division of Aquatic Sports; sdomali@phed.uoa.gr

* Correspondence: sdomali@phed.uoa.gr

**Abstract:** Artistic swimming training routinely involves various repeated high-intensity bouts that necessitate from athletes to learn and deliver the choreographed and technical movements required in competition. Mental fatigue may increase the rate of perceived exertion (RPE) without altering the physiological responses of athletes executing endurance-based exercise tasks (1). However, it remains unknown whether mental fatigue has similar effect following repeated, long-interval high-intensity efforts simulating competition. The aim of the present study was to assess the influence of mental fatigue on artistic swimmers’ physiological responses following long-interval, high-intensity choreography. Twelve female artistic swimming athletes (age: 17.5 ± 3.7 years, body mass: 20.7 ± 3.0 kg) completed a free routine of four repetitions and four-minute duration (4 × 4-min) with a two-min of passive rest in-between, on two occasions a week apart. Each occasion was preceded either by an implementation of a 30-min mental fatigue test (Stroop task; experimental condition; EC) or by watching an emotionally neutral video of similar duration (control condition; CC) in a randomized and counterbalanced order. Heart rate was continuously recorded during the routines (s610i, Polar electro, Finland). Fingertip blood samples were collected before the initiation, after the second, as well as at the end of the fourth 4-min routine and analyzed for blood lactate concentration (Lactate Scout+, SensLab GmbH). RPE was also recorded at the end of each 4-min routine. Heart rate was similar between conditions (EC: 172 ± 8 vs. CC: 174 ± 5 beats·min^−1^ *p* > 0.05, n = 8). Blood lactate concentration was higher following the second (6.4 ± 3.6 mmol·l^−1^) and the fourth bout (5.9 ± 3.7 mmol·l^−1^) compared to pre-exercise values (2.5 ± 1.5 mmol·l^−1^). Nonetheless, it was higher in CC compared to EC condition (6.6 ± 3.4 vs. 5.7 ± 3.8 mmol·l^−1^, *p* = 0.03). RPE was progressively increased over time being higher in the third (8.3 ± 1.2 a.u.) and fourth 4-min bout (8.4 ± 1.4 a.u.), compared to the first one (7.2 ± 0.9 a.u., *p* < 0.05), regardless of condition. Nevertheless, it was no different between conditions (EC: 7.7 ± 1.4 vs. CC: 8.1 ± 1.2 a.u., *p* > 0.05). Despite similar heart rate and perceived exertion values, the reduced blood lactate concentration following a stroop test indicates a lower activation of anaerobic metabolism; thus, suggesting that mental fatigue has likely a negative effect on repeated 4-min duration high-intensity efforts.

**Keywords:** mental fatigue; physiological characteristics; exercise performance; artistic swimming.

**Funding:** This research received no external funding.

**References**

1. Marcora, S.M.; Staiano, W.; Manning, V. Mental fatigue impairs physical performance in humans. *J Appl Physiol* **2009**, *106*, 857–864. doi:10.1152/japplphysiol.91324.2008.

### 2.39. The Effects of Six Weeks of High-Load Cluster Training versus Traditional High-Load Training on Half-Squat Strength and Power

Christos Paizis ^1,2,^*, Anthony Demoulin ^2^, Nicolas Amiez ^1,2^ and Nicolas Babault ^1,2^

^1^ CAPS, U1093 INSERM, Sport Science Faculty, University of Bourgogne Franche-Comté, 21078 Dijon, France; christos.paizis@u-bourgogne.fr; anthonydemoulin71@gmail.com; Nicolas.amiez@u-bourgogne.fr; nicolas.babault@u-bourgogne.fr

^2^ Center of Performance Expertise, Sport Science Faculty, 3 Allée des Stades Universitaires, BP 27877, CEDEX, University of Bourgogne Franche-Comté, 21078 Dijon, France

* Correspondence: christos.paizis@u-bourgogne.fr

**Abstract:** This study compares the effects of high-load cluster training (CLUST) versus traditional high-load training (TRAD) using the half-squat on changes in strength and power. The aim of this study is, therefore to find out whether, at heavier loads than those often studied in the literature, notably at 80% of 1RM or less, the cluster makes it possible to maintain a high level of power for longer than with work at equivalent loads in continuous effort (1,2). We hypothesize that the 9*1 repetition at 90% 1 repetition maximum (1RM) would enable greater power to be produced and therefore greater strength to be developed. Twenty resistance-trained individuals were divided into two matched groups. Group CLUST (n = 10) performed 9 sets of 1 repetition at 90% of 1RM with cluster having a 45-s inter-repetition, whereas Group TRAD (n = 10) performed the Traditional 3 sets of 3 repetitions at 90% of 1 repetition maximum (1RM) with a 3-min interset rest. The 1RM half-squat, the number of repetitions at 90% of the 1RM assessed in the pre-test, and the countermovement vertical jump (CMVJ) test were assessed 1 week before and 1 week after six weeks of training, including two sessions per week. Measures of changes of power were assessed during each repetition of all sessions. A 2-way analysis of variance was calculated to compare differences between-groups and Time (PRE, POST). 1RM half-squat strength, power, number of repetitions at 90% of 1RM assessed in the pre-test and CMVJ increased significantly in both the CLUST (*p* < 0.001 for all variables) and the TRAD (*p* < 0.001 for all variables) groups. No significant difference existed between groups for all variables (*p* > 0.05). Strength training in 90% of 1RM during six weeks can lead to similar gains in strength and power of half-squat despite differences in rest between the repetitions. We conclude that the CLUST would not be able to produce greater power and, therefore greater strength than TRAD. However, CLUST avoids any tendency to fail and is, therefore, safer and simpler for athletes to improve half-squat strength like TRAD.

**Keywords:** maximal strength; cluster; power; training.

**Funding:** This research received no external funding.

**References**

1. Iglesias-Soler, E.; Carballeira, E.; Sánchez-Otero, T.; Mayo, X.; Fernández-Del-Olmo, M. Performance of Maximum Number of Repetitions with Cluster-Set Configuration. *Int J Sports Physiol Perform* **2014**, *9*, 637–42.
2. Iglesias-Soler, E.; Mayo, X.; Río-Rodríguez, D.; Carballeira, E.; Fariñas J.; Fernández-Del-Olmo, M. Inter-Repetition Rest Training and Traditional Set Configuration Produce Similar Strength Gains without Cortical Adaptations. *J Sports Sci* **2016,** *34*, 1473–84.

### 2.40. Effect of Different Local Vibration Durations on Knee Extensors’ Maximal Isometric Strength

Christos Paizis ^1,2,^*, Stella Zwgrafou ^1^, Tom Timbert ^1^, Alain Martin ^1^, Spyridon Methenitis ^3,4^, Nicolas Babault ^1,2^ and Nicolas Amiez ^1^

^1^ INSERM UMR1093-CAPS, Université de Bourgogne, UFR des Sciences du Sport, F-21000, Dijon. Institut National de la Santé et de la Recherche Médicale: UMR1093, Université de Bourgogne, France; christos.paizis@u-bourgogne.fr; stella_zwgrafou@hotmail.com; tom.timbert@gmail.com; alain.martin@u-bourgogne.fr; nicolas.babault@u-bourgogne.fr; nicolas.amiez@u-bourgogne.fr;

^2^ Centre d’Expertise de la Performance, Université de Bourgogne, UFR des Sciences du Sport, F-21000, Dijon. Université de Bourgogne, France

^3^ Sports Performance Laboratory, School of Physical Education & Sports Science, National and Kapodistrian University of Athens, 15772 Athens, Greece; smetheni@phed.uoa.gr

^4^ Theseus, Physical Medicine and Rehabilitation Center, 17671 Athens, Greece

* Correspondence: christos.paizis@u-bourgogne.fr; Tel.: +33-3-80-39-67-29

**Abstract:** Prolonged application (>20 min) of local vibration (LV) on muscles or tendons is known to reduce maximal isometric strength (1–4). However, the effect of short vibration durations (<6 min) is still un-known. The aim of the present study was to identify the shortest duration that would induce a reduction in the knee extensor muscles’ force and to confirm the central origin of such losses. In fourteen participants, the changes in maximal voluntary isometric contraction (MVIC) were measured after 1, 3, and 6 min of rest (CONT) or local vibration (LV) over the quadricipital tendon (frequency: 100 Hz; amplitude: 0.5 mm). Before and after each condition, the amplitude of the twitch induced by a 100 Hz potentiated electrical doublet (PDPOT), relative electromyographic activity of the vastus medialis and rectus femoris muscle during the MVIC (RMSMVIC.M-1), the torque developed 50 ms after the onset of contraction (T50) and the voluntary activation level (VAL) were evaluated. Repeated measures ANOVAs with three within-subject factors [Condition (CONT, LV) × Duration (1, 3, 6) × Time (PRE, POST)] on raw data forall variables were performed. None of the three LV durations significantly changed MVIC compared with the control condition (*p* = 0.379). Indices of central (i.e., VAL, T50, RMS-MVIC.M-1) and peripheral (PDPOT) fatigue were unaffected (*p* > 0.147). In conclusion, a short duration of LV (<6 min) on a voluminous muscle group does not impair maximal force production nor induce any central or peripherical fatigue.

**Keywords:** local vibration; maximal voluntary isometric strength; knee extensor.

**Funding:** This research received no external funding.

**References**

1. Souron, R.; Besson, T.; McNeil, C.J.; Lapole, T.; Millet, G.Y. An Acute Exposure to Muscle Vibration Decreases Knee Extensors Force Production and Modulates Associated Central Nervous System Excitability. *Front Hum. Neurosci* **2017**, *11*.
2. Jackson, S.W.; Turner, D.L. Prolonged Muscle Vibration Reduces Maximal Voluntary Knee Extension Performance in Both the Ipsilateral and the Contralateral Limb in Man. *Eur J Appl Physiol* **2003**, *88*, 380–386.
3. Kouzaki, M.; Shinohara, M.; Fukunaga, T. Decrease in Maximal Voluntary Contraction by Tonic Vibration Applied to a Single Synergist Muscle in Humans. *J Appl Physio* **2000**, *89*, 1420–1424.
4. Konishi, Y.; Kubo, J.; Fukudome, A. Effects of Prolonged Tendon Vibration Stimulation on Eccentric and Concentric Maximal Torque and EMGs of the Knee Extensors. *J Sport Sci Med* **2009**, 8, 548–552.

### 2.41. Physiological Parameters Related to the Rate of Power Recovery during Resistance Exercise in Upper Limbs

Michail Panagiotopoulos ^1,^*, Athanasios Tsoukos ^1^, Charilaos Tsolakis ^1^, Gerasimos Terzis ^1^ and Gregory C. Bogdanis ^1^

^1^ School of P.E. and Sport Science, National and Kapodistrian University of Athens, Greece

* Correspondence: mixalispana@phed.uoa.gr

**Abstract:** The aim of this study was to examine the relationship between physiological parameters and the rate of power recovery during resistance exercise of the upper limbs. Fourteen men aged 25.0 ± 6.3 years (height: 1.78 ± 0.09 m, body mass: 78 ± 11 kg), with at least 2 years of experience in resistance training took part in this study. In a preliminary session, maximum bench press strength (1 repetition maximum, 1RM) and the optimum load for the highest power generation were measured in the horizontal bench press exercise on a Smith machine (95.0 ± 17.4 kg and 56.3 ± 1.4% of 1RM, respectively). Also, the slope of performance decline was calculated during a maximum number of repetitions (MNRslope) test, executed as fast as possible against the optimum load. On another visit maximum aerobic power of the upper limbs was determined via an increasing load protocol on an arm crank ergometer (−0.026 ± 0.006 m/s/rep and 104.6 ± 18.1 W, respectively). In the next five sessions, participants performed four 10 s sets of bench press executed as fast as possible, against an external load corresponding to the optimal load. The rest interval between sets was either 30 s or 1 or 2 or 3 or 5 min in a random and counterbalanced order. During the exercise, bar velocity and mean power of all repetitions were measured with a linear position transducer. The percentage of bench press performance recovery between the 1st and the 2nd set was calculated to provide the individual rate of power recovery. Multiple linear regression showed that 68% (*p* < 0.01) of the variation in the rate of power recovery could be explained by MNRslope and maximum theoretical velocity (V0). Furthermore, high correlations were found between power recovery and the rate of decrease in mean velocity per repetition in the MNR test (r = −0.605 to −0.633, *p* = 0.015–0.022), as well as between the aerobic power of the upper limbs expressed per kilogram of body mass and power recovery (r = 0.54, *p* = 0.047). Neural networks analysis showed that the time required for a person to achieve 95% power recovery from the 1st to the 2^n^ set could be predicted with an accuracy of 94–99%. Power recovery could be predicted by the combination of MNR slope, V0, and by the rate of decreasing of the mean velocity per repetition or aerobic power.

**Keywords:** resistance training; bench press; linear position transducer.

**Funding:** This research received no external funding.

**References**

1. Davó, J.L.H.; Ruiz, J.B.; Sabido, R. Influence of strength level on the rest interval required during an upper-body power training session. *J Strength Cond Res* **2017**, *31*, 339–347.
2. González-Hernández, J.M.; Jimenez-Reyes, P.; Janicijevic, D.; Tufano, J.J.; Marquez, G.; Garcia-Ramos, A. Effect of different interset rest intervals on mean velocity during the squat and bench press exercises. *Sports Biomech* **2020**, *1*, 14.

### 2.42. Gastrocnemius Medialis Fascicle Length in Female Athletes of Different Maturity Status

Ioli Panidi ^1,^ *, Gregory C. Bogdanis ^1^, Vasiliki Gaspari ^1^, Gerasimos Terzis ^1^, Anastasia Donti ^1^, Andreas Konrad ^2^ and Olyvia Donti ^1^

^1^ School of Physical Education & Sport Science, National and Kapodistrian University of Athens, 17237 Athens, Greece; ipanidi@phed.uoa.gr; gbogdanis@phed.uoa.gr; vgaspari@phed.uoa.gr; gterzis@phed.uoa.gr; adonti@phed.uoa.gr; odonti@phed.uoa.gr

^2^ Institute of Human Movement Science, Sport and Health, University of Graz, Graz, Austria; andreas.konrad@uni-graz.at

* Correspondence: ipanidi@phed.uoa.gr; Tel.: +30-21-0727-6109

**Abstract:** Fascicle length is a muscle architectural characteristic related to muscle extensibility and performance. Systematic flexibility training and maturational growth have a significant effect on muscle fascicle length (1), but data on growing athletes are sparse (2). The aim of this study was to examine gastrocnemius medialis fascicle length (FL) in female athletes of different ages and stages of maturity. Furthermore, the association between FL and athletes’ age, training experience and anthropometric characteristics was also examined. FL at the medial part of gastrocnemius medialis was assessed in 99 female volleyball athletes, aged 8–18 years, using ultrasonography. Athletes were assigned to three maturation groups: pre-pubertal (n = 25), circa-pubertal (n = 26) and post-pubertal (n = 48). Height, sitting height, body mass, leg- and calf length were also measured. As expected, muscle fascicles were longer in post-pubertal compared to pre- and circa-pubertal athletes. FL was similar between circa-pubertal compared to pre-pubertal girls (4.11 vs. 3.99 cm, respectively, *p* = 0.702), but was longer by 8% in post-pubertal compared to circa-pubertal (4.46 vs. 4.11 cm, respectively, *p* = 0.034) and by 12% in post-pubertal compared to prepubertal (4.46 vs. 3.99 cm, respectively, *p* = 0.003). Moderate correlations were found between FL and athletes’ age, training experience and anthropometric characteristics (*r* = 0.333 to 0.482, *p* < 0.001). Multiple regression analyses indicated that in pre-pubertal girls, calf length and training experience explained 28.1% of the variance in FL (*p* = 0.010), while in circa- and post-pubertal girls body mass accounted for 31 and 12.5% of the variance in FL, respectively (*p* = 0.002 and *p* = 0.008, respectively). In conclusion, FL in pre-pubertal athletes was associated with calf length and the mechanical loading imposed on the muscles during training. In circa-and post-pubertal athletes, FL was more related to body and muscle mass, possibly due to accelerated maturation.

**Keywords:** muscle architecture; growth; puberty; ultrasound; volleyball.

**Funding:** This research received no external funding.

**References**

1. Panidi, I.; Bogdanis, G.C.; Terzis; G.; Donti, A.; Konrad, A.; Gaspari, V.; Donti, O. Muscle architectural and functional adaptations following 12-weeks of stretching in adolescent female athletes. *Front Physiol* **2021**, *12*, 701338.
2. Panidi, I.; Donti, O.; Konrad, A.; Dinas, P.C.; Terzis, G.; Mouratidis, A.; Gaspari, V.; Donti, O.; Bogdanis, G.C. Muscle Architecture Adaptations to Static Stretching Training: A Systematic Review with Meta-Analysis. *Sports Med-Open* **2023**, *9*, 1–27.

### 2.43. Effects of an Extreme Ultra-Endurance Cycling Race on Muscle Damage Indices and Body Composition; a Case Study

Ioannis Papanikolaou ^1^ and Giannis Arnaoutis ^2,^*

^1^ BIKE Sale & Academy; ioannispapanikolaou@gmail.com

^2^ School of Health Sciences and Education, Department of Nutrition & Dietetics, Harokopio University; garn@hua.gr

* Correspondence: garn@hua.gr; Tel.: +302109549321

**Abstract:** Ultra-endurance events have gained significant popularity during the last decades worldwide. However, research concerning biochemical indices and performance data in racing athletes is scarce. The purpose of the present study was to present a case report of a well-trained ultra-endurance cyclist (years: 42, VO_2_ peak: 50.8 mL min ^−1^·kg ^−1^) during an extreme endurance cycling race (3779 km total distance, 302.3 ± 104.6 km/day) which was completed in 12 days, 11 h and 49 min. Body composition characteristics and blood samples were collected and analyzed pre- and post-race. Athlete’s raw data and environmental conditions were recorded daily throughout the race. Body fat mass reduced from 7.8 to 6.1 kg post-race while on the contrary, muscle mass increased (pre: 62.6; post 67.5 kg). Extracellular Body Water increased from 17.5 to 19.7 L after the completion of the race. Creatine Kinase (pre: 208; post: 358 U/L), Lactate DeHydrogenase (pre: 188; post: 266 U/L), SGOT (pre: 29; post: 48 U/L) and SGPT (pre: 31; post: 61 U/L) were all significantly elevated above reference values post-race. Mean cycling speed during the course was 18.7 km/h (ranging from 15.2 to 26.5 km/h), while mean cycling power was measured at 98 Watts (ranging from 83 to 125 watts). Mean environmental temperature throughout the 12 racing days was 23.4 °C (ranging from 0 to 45 °C). Although specific recommendations cannot be given on the basis of these data, this report indicates significant muscle damage, and possible edema due to high water retention during an extreme endurance event. Individual nutrition approaches and racing strategies should be implemented and investigated.

**Keywords:** ultra-endurance; cycling; nutrition; body composition.

**Funding:** This research received no external funding.

**References**

1. Rothschild, J.A.; Delcourt, M. Racing and Training Physiology of an Elite Ultra-Endurance Cyclist: Case Study of 2 Record-Setting Performances. *IJSPP* **2021**, *16*, 739–743.
2. Valenti, M.T.; Bragio, M.; et al. Effects of a 4400 km ultra-cycling non-competitive race and related training on body composition and circulating progenitors differentiation. *J Transl Med* **2022**, *20*, 397.

### 2.44. Sex Differences in Balance Control and Jumping Performance before the Age of Peak Height Velocity

Zacharoula Paschaleri ^1,^*, Theodoros Kannas ^1^, Georgios Chalatzoglidis ^1^ and Fotini Arabatzi ^1^

^1^ Laboratory of Neuromechanics, Department of Physical Education and Sports Science, Aristotle University of Thessaloniki, Greece; zpaschal@phed-sr.auth.gr; thkannas@phed-sr.auth.gr; gchalatzo@phed-sr.auth.gr; farabaji@phed-sr.auth.gr

* Correspondence: zpaschal@phed-sr.auth.gr

**Abstract:** Jumping and balancing are common activities for adolescents, not only in their daily lives, but also in competitive sports. While movement efficiency appears to improve during adolescence, the rapid increase in height around peak height velocity (PHV) may impede this improvement. These disturbances may be due to neuromuscular and sensorimotor adaptations, as well as structural and neural changes during the growth spurt. The full scope of the aforementioned subject is currently not entirely clear. Thus, this study seeks to investigate the potential mechanisms involved in performing jumping and balancing tasks during the maturation period of adolescence. Additionally, this study aims to examine the effects of the growth spurt on such movements. Twenty-three boys (average age: 12.5 ± 0.29) and twenty-two girls (average age: 10.5 ± 0.32) participated. We calculated each participant’s time relative to PHV. The study had three assessment points: 18 and 9 months before PHV, and at PHV. Participants completed balance tests (bilateral, unilateral, and with eyes closed) and a countermovement jump (CMJ). Additionally, Achilles tendon (AT) and plantar flexor muscle forces were measured using a dynamometer. Muscle activity in the medial gastrocnemius (MG) and tibialis anterior (TA) was measured with surface EMG electrodes during balance tasks and the jump’s phases. Regression analysis showed a correlation between increased AT stiffness and contraction amplitude of plantar flexors during a closed eyes task, 9 months before peak height velocity (PHV), in girls. Meanwhile, it demonstrated a link between increased AT stiffness and increased TA activity during bilateral, closed eyes and unilateral balance tasks, in boys, 18 and 9 months before PHV. Linear Mix Model analysis showed a significant interaction between sex and AT stiffness for both jump velocity and jump height, where boys had higher values than girls. We have discovered unique strategies that early adolescent boys and girls rely on to perform static balancing maneuvers. Boys adopt a peripheral mechanism characterized by high AT stiffness and increased TA activity. In contrast, girls exhibit central balance regulation with a more compliant AT and elevated co-activation of plantar flexors. Superior performance in boys regarding CMJ is associated with higher AT stiffness, though maturity growth designates a plateau phase in jump performance near PHV.

**Keywords:** adolescence; balance control; jumping performance; Achilles tendon stiffness; plantar flexors force; muscle activity.

**Funding:** This research received no external funding.

**References**

1. Lefevre. J.; Beunen, G.; Steens, G.; Claessens, A.; Renson R. Motor performance during adolescence and age thirty as related to age at peak height velocity. *Ann Hum Biol* **1990**, *17*, 423–35.
2. Blazevich, A.; Waugh, C.; Korff T. Development of musculoskeletal stiffness. *Paediatric Biomechanics and Motor Control: Routledge* **2013**, 96–118.
3. Warnica, M.J.; Weaver, T.B.; Prentice, S.D.; Laing, A.C. The influence of ankle muscle activation on postural sway during quiet stance. *Gait & Posture* **2014**, *39*, 1115–21.
4. Choi, Y.J.; Chalatzoglidis, G.; Trapezanidou, M.; Delmas, S.; Savva, E.; Yacoubi, B.; et al. Adolescent boys who participate in sports exhibit similar ramp torque control with young men despite differences in strength and tendon characteristics. *Eur J Appl Physiol* **2023**, 1–10.

### 2.45. Variations and Correlations of Neuromuscular Performance and Match External Load Metrics in Professional Football Players across the Season

Laura Payton ^1^, Konstantinos Spyrou ^1,2,3,^*, Antonio Martínez-Serrano ^1,2,3^ and Pedro E. Alcaraz ^1,2,3^

^1^ UCAM Research Center for High Performance Sport, UCAM Universidad Católica de Murcia, Murcia Spain; lkpayton@alu.ucam.edu; kspyrou@ucam.edu; amartinez30@ucam.edu; palcaraz@ucam.edu

^2^ Facultad de Deporte UCAM Universidad Católica de Murcia, Murcia Spain

^3^ Strength and Conditioning Society, Murcia, Spain

* Correspondence: kspyrou@ucam.edu

**Abstract:** In soccer, external match-load management and testing is used to facilitate training session planning and recovery, maintain performance, and reduce injury risk (1). However, to effectively monitor post-match changes in physical performance, valid, reliable and practical measures which are sensitive to change are required (2). Despite the previous research on post-game neuromuscular fatigue (NMF) and external match-load (3), their correlations and variation of these metrics across a season, have not been examined. The aim of this study was to determine the variation and correlations between match external load metrics, kinetic countermovement jump (CMJ), and 90:20 isometric posterior chain (IPC) test variables across the season. Match-day physical demands (total distance (TD), high-speed running distance (HSRD), and metres per minute) and 24 h post-game NMF (kinetic CMJ metrics and 90:20 IPC) performance testing data were collected by 19 footballers in Spanish La Liga Division Segunda B during the 2020–2021 season. Jamovi software (Jamovi version 2.3.24, Sydney, Australia) and costumed Microsoft Excel used for the statistical analysis. Coefficient variation (CV), intraclass coefficient variation (ICC), typical error (TE), and correlation for all the metrics was performed. The kinetic CMJ and 90:20 metrics CV was low (<10%) across the season, except eccentric peak power presented 13.4% CV. Regarding match external load, TD and HSRD metrics presented high 14.9% and 21.6% respectively, CV. ICC was good to excellent for all the variables. Significant positive correlations were observed between CMJ height and the maximal force produced in the 90:20 test (*p* < 0.001). HSRD and metres per minute (m/min) were negatively correlated with CMJ peak power (*p* < 0.001). m/min. Significant positive and negative correlations found between CMJ kinetic metrics. The variation for all fatigue-indicator variables across the season was relatively low, demonstrating low variation in NMF from the season start to end following a match. The correlations observed may help practitioners in monitoring season load and highlight variables to focus on when monitoring NMF.

**Keywords:** soccer; countermovement jump; isometric strength; sensitivity.

**Funding:** This research received no external funding.

**References**

1. Bengtsson, H.; Ekstrand, J.; Hägglund, M. Muscle injury rates in professional football increase with fixture congestion: an 11-year follow-up of the UEFA Champions League injury study. *Br J Sports Med* **2013**, *47*, 743–7.
2. Hader, K.; Rumpf, M.C.; Hertzog, M.; Kilduff, L.P.; Girard, O.; Silva, J.R. Monitoring the Athlete Match Response: Can External Load Variables Predict Post-match Acute and Residual Fatigue in Soccer? A Systematic Review with Meta-analysis. *Sports Med-Open* **2019**, *5*, 48.
3. Martínez-Serrano, A.; Freitas, T.T.; Franquesa, X.; Enrich, E.; Mallo, M.; Alcaraz, P.E. Does External Load Reflect Acute Neuromuscular Fatigue and Rating of Perceived Exertion in Elite Young Soccer Players? *J Strength Cond Res* **2023**, *37*, e1–e7.

### 2.46. Effect of Different High Intensity Interval Training Modalities on Aerobic Capacity and Lower Body Muscle Power

Ilias–Iason Psarras ^1^, Athanasios Tsoukos ^1^, Gerasimos Terzis ^1^, Giorgos Paradisis ^1^ and Gregory C. Bogdanis ^1,^*

^1^ School of Physical Education and Sports Science, National and Kapodistrian University of Athens, 17237, Athens, Greece; ipsarras@phed.uoa.gr; atsoukos@phed.uoa.gr; gterzis@phed.uoa.gr; gparadi@phed.uoa.gr; gbogdanis@phed.uoa.gr

* Correspondence: gbogdanis@phed.uoa.gr; Tel.: +30-210-7276115

**Abstract:** The time available for improving physical fitness in soccer is limited (1). This study investigated the physiological and neuromuscular adaptations of an integrated high intensity interval aerobic and power training protocol during the off-season period (2). Twenty-four young high level soccer players (age: 14.9 ± 0.2 years, maturity offset: 1.4 ± 0.6 years, height: 175.1 ± 8.3 cm, body mass: 64.9 ± 9.3 kg), were divided into three groups and performed a 2 × 6 min interval training protocol with 2 min passive recovery between sets, two times per week for 6 weeks. Each 6 min set included repeated periods of 15 s exercise interspersed with 15 s passive rest. One group (RUN) performed all exercise periods with running at 10% of anaerobic speed reserve. The second group (JUMP) performed these periods with jumping, i.e., each 6-min set included four periods of jumping exercise (9 drop jump per 15 s work interval separated with 75 s rest intervals). The third group performed an integrated protocol (INT), in which each 6-min set included four periods of jumping exercise (9 drop jump per 15 s work interval) in combination with running (i.e., one 15 s jumping period every two running 15 s periods) (3, 4). The Yo-Yo Intermittent Recovery Level 1 (YYIR1) test was conducted to evaluate aerobic capacity. Countermovement jump (CMJ), 10 m, and 30 m sprint were used to assess neuromuscular adaptations. A two-way mixed ANOVA was conducted to find the differences between the three groups. After 6 weeks of training, all groups improved similarly in all parameters evaluated, except 10 m sprint performance which decreased by 2.2% in all groups (main effect of time for YYIR1: 1828 m ± 32 m to 1963 m ± 79 m, *p* < 0.05; 10 m sprint: 1.81 s ± 0.07 s to 1.85 s ± 0.04 s, *p* = 0.016; 30 m sprint 4.46 s ± 0.2 s to 4.41 s ± 0.19 s, *p* = 0.018), while CMJ remained unchanged (43.4 cm ± 0.35 cm to POST: 43.7 cm ± 0.3 cm, *p* = 0.74). The results of this study suggest that all methods may be effective for improving aerobic fitness and maintaining leg power at high levels during the pre-season in elite youth soccer players but could not prevent a small decrease in sprinting ability.

**Keywords:** high intensity interval training; plyometric training; neuromuscular performance.

**Funding:** This research received no external funding.

**References**

1. Buchheit, M.; Laursen, P.B. High-Intensity Interval Training, Solutions to the Programming Puzzle: Part I: Cardiopulmonary Emphasis. *Sports Med* **2013**, *43*, 313–38.
2. Ducrocq, G.P.; Hureau, T.J.; Meste, O.; Blain, G.M. Similar Cardioventilatory but Greater Neuromuscular Stimuli with Interval Drop Jump Than with Interval Running. *Int J Sports Physiol Perform* **2020**, *15*, 330–9.
3. Enright, K.; Morton, J.; Iga, J.; Drust, B. The effect of integrated training organization in youth elite soccer players. *Eur J Appl Physiol* **2015**, *115*, 2367–81.
4. Kramer, A.; Poppendieker, T.; Gruber, M. Suitability of jumps as a form of high-intensity interval training: effect of rest duration on oxygen uptake, heart rate and blood lactate. *Eur J Appl Physiol* **2019**, *119*, 1149–56.

### 2.47. The Impact of Static and Dynamic Exercises on Cardiac Para-Sympathetic Modulation

Vinod R.K.B.M.R. Rajapakshe ^1^ and Sajith M. A. Udayanga ^1^*

^1^ University of Sri Jayewardenepura, Sri Lanka; sajithudayanga@sjp.ac.lk

* Correspondence: sajithudayanga@sjp.ac.lk; Tel.: +94717431349

**Abstract:** The present study explored how static and dynamic stretching exercises affect the cardiac parasympathetic modulation of the autonomic nervous system (ANS), particularly in the context of sports, where understanding these differences is crucial for greater performance. Thirty-two individuals participated in this study, comprising 16 males (Age: 22.81 ± 1.56 years, BMI: 22.4 ± 3.09 kg/m^2^) and 16 females (Age: 22.56 ± 1.31 years, BMI: 20.34 ± 3.11 kg/m^2^). Participants from the static stretching (SS) group engaged in SS routines lasting 5 min, while those in the dynamic stretching (DS) group performed DS exercises for the same duration. After a 48-h washout period, participants who initially practiced SS switched to DS for 5 min, while those who started with DS switched to SS for 5 min. Before engaging in their respective exercises, both groups ran for 5 min at a self-selected pace in both scenarios. To monitor changes in cardiac parasympathetic modulation, we used a Polar H10 heart rate monitor to record RR data for 5 min duration before and after the interventions. Subsequently, we analyzed the recorded data using the Kubios HRV version 3.3.1 software (Biosignal Analysis and Medical Imaging Group, Department of Physics, University of Kuopio, Kuopio, Finland). Before analyzing the data, an artifact was corrected using the medium threshold artifact correction function. If the number of sample artifacts of the relevant RR interval sample was more than 5%, the sample was not included for the study (1). The root mean square of successive differences between normal heartbeats (RMSSD) data were obtained following the analysis (2). In both situations, the post-test findings revealed a significant decrease in the RMSSD parameter ((SS: M = 35.32, SD = 28.10; *p* > 0.0001), (DS: M = 25.45, SD = 25.82; *p* > 0.0001)) when compared to the pre-test ((SS: M = 51.26, SD = 27.83), (DS: M = 55.78, SD = 31.50)) results. Additionally, when comparing the impact of the two treatments, it became evident that DS led to a greater and statistically significant reduction (*p* = 0.001) in the RMSSD parameter. The findings of the study show that both static and dynamic exercises decrease cardiac parasympathetic dominance in the autonomic nervous system. Interestingly, DS exercises demonstrated a greater effect on the reduction of cardiac parasympathetic modulation in healthy individuals. In conclusion, these findings may suggest that including DS exercises in the warm-up session has a more positive impact on an athlete’s readiness for physical activities compared to SS.

**Keywords:** dynamic stretching; HRV; parasympathetic modulation; RMSSD; static stretching.

**Funding:** This research received no external funding.

**References**

1. Tarvainen, M.; Lipponen, J.; Niskanen, J.; Ranta-Aho, P. Kubios HRV Version 3–User’s Guide. 2017.
2. Shaffer, F.; Ginsberg, J. An overview of heart rate variability metrics and norms. *Front Public Health* **2017**, 5, 258.

### 2.48. Variability in Mechanical Properties of Hamstring Tendons

Chrysostomos Sahinis ^1,^* and Elefterios Kellis ^1^

^1^ Laboratory of Neuromechanics, Department of Physical Education and Sport Sciences at Serres, Aristotle University of Thessaloniki; sachinic@phed-sr.auth.gr; ekellis@phed-sr.auth.gr

* Correspondence: sachinic@phed-sr.auth.gr; Tel.: +30-6988043758

**Abstract:** The biomechanical properties of human tendons are crucial in understanding their function and the vulnerability to injury (1). Previous studies have documented great variability between and within hamstrings in their architectural characteristics (2) and in tendon morphology (3). While the importance of hamstring tendons is well documented, there is a limited understanding regarding hamstrings tendon mechanical properties. Three-dimensional ultrasound (US) images of the medial and lateral hamstring tendons were obtained from fifteen healthy individuals both at rest and during 20%, 40%, 60%, and 80% of their maximum voluntary isometric contraction (MVC) of knee flexors. US images underwent a manual digitization process to create a three-dimensional representation of the hamstring tendons. Subsequently, the reconstructed tendon models were used to calculate tendon length, volume, and cross-sectional area (CSA) in three measurement locations across the tendon length. Tendon length, volume and CSA strain at the different force levels were quantified relative to the resting condition. Semitendinosus showed the greatest tendon elongation (strain) as well as the highest CSA strain while the biceps femoris short head presented the lowest tendon elongation compared to other tendons (*p* < 0.05). No differences in tendon volume strain were found between tendons and force levels (*p* > 0.05). Further, tendons deformation was region-specific with the proximal site demonstrating the lowest CSA strain compared to other measurement sites. The present results suggest that there were significant differences in the mechanical properties between hamstrings tendons, emphasizing the importance to consider their distinct biomechanical characteristics when assessing vulnerability to injury and function. These findings contribute to our understanding of hamstring tendon mechanics and may have implications for the development of specified injury prevention and rehabilitation strategies.

**Keywords:** hamstrings; tendon mechanics; semitendinosus; biceps femoris; tendon strain; ultrasound.

**Funding:** This research received no external funding.

**References**

1. Magnusson, S.P.; Kjaer, M. The impact of loading, unloading, ageing and injury on the human tendon. *J Physiol* **2019**, *597*, 1283–1298.
2. Kellis, E. Intra- and Inter-Muscular Variations in Hamstring Architecture and Mechanics and Their Implications for Injury: A Narrative Review. *Sport Med* **2018**, *48*, 2271–2283.
3. Sahinis, C.; Kellis, E.; Dafkou, K.; Ellinoudis, A. Reliability of Distal Hamstring Tendon Length and Cross-sectional Area Using 3-D Freehand Ultrasound. *Ultrasound Med Biol* **2021**, *47*, 2579–2588.

### 2.49. Effects of Velocity Loss Thresholds on the Evolution of 1RM during Four Resistance Training Programmes with Blood Flow Restriction Implementation

Juan Sánchez-Valdepeñas ^1,2,^*, Pedro J. Cornejo-Daza ^1,2^, Luis Rodiles-Guerrero ^1,3^, José Paez-Maldonado ^1^, Beatriz Bachero-Mena ^1,3^, Miguel Sánchez-Moreno ^1,3^, Eduardo Saez de Villareal ^1,2^ and Fernando Pareja-Blanco ^1,2^

^1^ Physical Performance & Sports Research Centre, University Pablo de Olavide, Sevilla, Spain; jsanmat@upo.es

^2^ Faculty of Sport Sciences, Department of Sports and Computers Sciences, University Pablo de Olavide, Sevilla, Spain

^3^ Department of Physical Education and Sport, Universidad de Sevilla, Sevilla, Spain

* Correspondence: jsanmat@upo.es

**Abstract:** The implementation of blood flow restriction (BFR) during resistance training (RT) has increased markedly due to the higher induced adaptations in relation with strength and muscle hypertrophy (1). Furthermore, velocity loss (VL) is well known as a critical variable to provide valid information about the degree of fatigue necessary to maximize strength gains and physical performance (2). However, the use of VL during BFR-RT programmes has not been studied yet. For that, the aim of this study was to analyze the effects of four different VL thresholds (0%, 10%, 20% and 40%) with BFR implementation on the strength performance measured by the evolution of 1RM during each training session throughout the different resistance training programmes. Forty-nine strength-trained men took part in the study (BFR_0_ (n = 12), BFR_10_ (n = 12), BFR_20_ (n = 12) and BFR_40_ (n = 13)). Participants carried out an eight-week (twice per week) VBRT program ranging from 55 to 70% of 1RM using the full squat (FS). Subjects performed 3 sets per training session including BFR implementation at 50% of individualized maximal arterial occlusion pressure with 2 min of inter-set recovery. The specific warm-up consisted of 2 set at 40% (six repetitions) and 50% (four repetitions) of 1RM respectively separated by 3 min. Before and after the RT programmes, participants performed an isoinertial progressive loading test in the FS exercise prior to estimate the individual load-velocity relationship and 1RM at pre- and post- training. 1RM estimations were obtained during each training sessions and at pre- and post- training using the common load employed during the specific warm-up (50% 1RM). Although no significant group x time interaction was observed, BFR_10_ reported significant differences in comparison with BFR_40_ at session seven (*p* = 0.008). Regarding intra-group changes, BFR_10_ attained significant increases in 1RM strength from pre- to session seven (*p* = 0.02) whereas BFR_20_ and BFR_40_ reported significant enhancements from pre- to post training (*p* < 0.001 and *p* = 0.03, respectively). It should be noted that during the training programmes there was a tendency to greater increases for BFR_0,_ BFR_10_ and BFR_20_ compared to BFR_40_. These results show that low to moderate velocity loss thresholds attained within the set with BFR implementation could be a better choice to improve 1RM strength in FS throughout and at the end of resistance training programmes.

**Keywords:** blood flow restriction; velocity based resistance training; velocity loss; full squat.

**Funding:** This research received no external funding.

**References**

1. Lixandrão, M.E.; Ugrinowitsch, C.; Berton, R.; Vechin, F.C.; Conceição, M.S.; Damas, F.; Libardi, C.A.; Roschel, H. Magnitude of Muscle Strength and Mass Adaptations Between High-Load Resistance Training Versus Low-Load Resistance Training Associated with Blood-Flow Restriction: A Systematic Review and Meta-Analysis. *Sports Med* **2018**, *48*, 361–378.
2. Pareja-Blanco, F.; Alcazar, J.; Sánchez-Valdepeñas, J.; Cornejo-Daza, P.J.; Piqueras-Sanchiz, F.; Mora-Vela, R.; Sánchez-Moreno, M.; Bachero-Mena, B.; Ortega-Becerra, M.; Alegre, L.M. Velocity Loss as a Critical Variable Determining the Adaptations to Strength Training. *Med Sci Sports Exerc* **2020**, *52*, 1752–1762.

### 2.50. The Effect of Tooth Loss on Walking Variability in Elderly People

Vasiliki Savva ^1,^*, Martha Trapezanidou ^2^, Georgios Chalatzoglidis ^2^, Alexandros Tsouknidas ^3^, Anastassios Philippou ^1,4^ and Fotini Arabatzi ^2^

^1^ School of Medicine at the National and Kapodistrian University of Athens; vickysavva48@gmail.com; tfilipou@med.uoa.gr

^2^ Laboratory of Neuromechanics, Department of Physical Education and Sports Science, Aristotle University of Thessaloniki, Greece; mtrapezan@phed-sr.auth.gr; gchalatzo@phed-sr.auth.gr; farabaji@phed-sr.auth.gr

^3^ Laboratory for Biomaterials and Computational Mechanics, Department of Mechanical Engineering, University of Western Macedonia, 50100 Kozani, Greece; atsouknidas@uowm.gr

^4^ Physiology Laboratory in the School of Medicine at the National and Kapodistrian University of Athens

* Correspondence: vickysavva48@gmail.com

**Abstract:** Gait instability are major risk factors for falling, particularly in elderly people (1,2). Several recent reports have indicated that tooth loss also has a negative impact on body balance and stride variability in elderly especially under dual task condition (3,4). The purpose of this study is to investigate the influence of tooth loss and wearing complete dentures on walking at three different conditions. Healthy fourteen dentulous (14) and twenty-one edentulous patients (21) over the age of 65 years. participated in this study. All the edentulous subjects were wearing complete dentures and executed the measurements under two conditions: wearing dentures and not wearing dentures. Gait stability was evaluated by measuring the parameters gait velocity and stride-time variability in a 20-m distance, during self-selected normal walking speed, fast walking speed and under dual-task performance, with a tri-axial accelerometer at a sampling rate of 128 Hz. Differences for the above variables were assessed with the two-way ANOVA (*p* < 0.05). Dentulous participants had a significantly lower stride-time variability compared to edentulous participants with denture wear in all conditions (*p* = 0.03). Only in dual task condition dentulous participants differenced significant with edentulous participants irrespective wearing and not wearing dentures exhibiting higher stride stability (*p* = 0.012). Complete dentures produced an effect on the gait velocity stability of edentulous patients under all conditions. The present results suggest that tooth loss in healthy seniors is associated with lower gait velocity and therefore may have a negative impact on gait stability.

**Keywords:** Gait–stride instability; elderly; dentulous; edentulous participants

**Funding:** This research received no external funding.

**References**

1. Okubo, M.; Fujinami, Y.; Minakuchi, S. Effect of complete dentures on body balance during standing and walking in elderly people. *J Prosthodont Res* **2010**, *54*, 42–47.
2. Brand, C.; Bridenbaugh, S.A.; Perkovac, M.; Glenz, F.; Besimo, C.E.; Sendi, P.; Kressig, R. W.; Marinello, C.P. The effect of tooth loss on gait stability of community-dwelling older adults. *Gerodontology* **2015**, *32*, 296–301.
3. Granacher, U.; Bridenbaugh, S.A.; Muehlbauer, T.; Wehrle, A.; Kressig, R.W. Age-related effects on postural control under multi-task conditions. *Gerontology* **2011**, *57*, 247–55.
4. Alcock, L.; O’Brien, T.D.; Vanicek, N. Association between somatosensory, visual and vestibular contributions to postural control, reactive balance capacity and healthy ageing in older women. *Health Care Women Int* **2018**, *39*, 1366–1380.

### 2.51. Bone Marrow Residual Tumor Cells after Medical Treatment of Multiple Myeloma Is Not Associated with Physical Function and Quality of Life

Polyxeni Spiliopoulou ^1,^*, Evangelos Eleutherakis Papaiakovou ^2^, Magda Migou ^2^, Nick Kanellias ^2^, Foteini Theodorakakou ^2^, Despina Fotiou ^2^, Ioannis Ntanassis-Stathopoulos ^2^, Amalia Andreatou ^2^, Pantelis Rousakis ^3^, Maria Gavriatopoulou ^2^, Ourania E. Tsitsilonis ^3^, Efstathios Kastritis ^2^, Meletios A. Dimopoulos ^2^ and Gerasimos Terzis ^1^

^1^ Sports Performance Laboratory, School of Physical Education and Sport Science, National and Kapodistrian University of Athens, Athens, Greece; spl@phed.uoa.gr

^2^ Department of Clinical Therapeutics, School of Medicine, National and Kapodistrian University of Athens, Alexandra General Hospital, Athens, Greece

^3^ Department of Biology, School of Sciences, National and Kapodistrian University of Athens, 15784 Athens, Greece

* Correspondence: spipolyxeni@phed.uoa.gr

**Abstract:** Multiple myeloma is a type of cancer detected in the bone marrow plasma cells affecting life quality and expectancy as well as everyday physical function. The presence of >10% of abnormal plasma cells in the bone marrow is a diagnostic criterion for multiple myeloma (1). After an effective first line medical treatment there are patients that no abnormal plasma cells are detected, while there are others with residual abnormal plasma cells in their bone marrow (about 0.001–0.01%). Yet, it is unknown if there is a correlation between the residual abnormal plasma cells and physical function or quality of life in multiple myeloma patients. The aim of the study was to examine if there is difference in physical function and quality of life between multiple myeloma patients with residual abnormal plasma cells and patients with undetectable abnormal plasma cells in the bone marrow, after the first line medical treatment. Fourteen multiple myeloma patients (4 females, 10 males; age 47.2 ± 5.9 years; mass 79.6 ± 21.4 kg) were included in the study. After completing the first line medical treatment, bone marrow samples were collected from the posterior iliac crest with a bone marrow aspiration needle after local anesthesia. Abnormal plasma cells of the bone marrow were evaluated with flow cytometry. Quality of life (SF-36 questionnaire), six-minute walking test, handgrip strength (sum of both hands) and maximal aerobic power in a cycle ergometer were assessed one week later. In seven patients no abnormal plasma cells were detected in the bone marrow (PC group), while in the other seven patients 0.009 ± 0.018% of the total bone marrow nucleated cells were abnormal plasma cells (APC group). Quality of life was similar between groups (*p* > 0.05). Six-minute walking test performance was 520 ± 64 m for the APC group and 497 ± 72 m for the PC group with no difference between groups (*p* = 0.535). Handgrip strength was similar between APC and PC (73 ± 8 kg, and 67 ± 17 kg, respectively, *p* > 0.05). No statistical difference was found in maximal aerobic power between groups (APC: 116.7 ± 20.4 Watt, PC: 89.3 ± 31 Watt, *p* = 0.098). Detection of abnormal plasma cells in the bone marrow after the first line medical treatment is a negative clinical outcome for multiple myeloma patients. Nevertheless, it does not affect quality of life and physical function of these patients. Further studies are needed to investigate these parameters in long term.

**Keywords:** multiple myeloma; physical exercise; exercise oncology.

**Funding:** This research was funded by Stavros Tsakirakis Scholarships, grant number 16257.

**References**

1. Rajkumar, S.V.; Kumar, S. Multiple Myeloma: Diagnosis and Treatment. *Mayo Clin Proc* **2016**, *91*, 101–119.

### 2.52. External Match Load and the Differences between Halves in Female Futsal

Konstantinos Spyrou ^1,2,3,^*, Pedro E. Alcaraz ^1,2,3^, Juan Alcaraz Ibáñez ^4^, Elena Marín-Cascales ^1,2,3^ and Tomás T. Freitas ^1,2,3^

^1^ UCAM Research Center for High Performance Sport, UCAM Universidad Católica de Murcia, Murcia Spain; kspyrou@ucam.edu; palcaraz@ucam.edu; emarin@ucam.edu; tfreitas@ucam.edu

^2^ Facultad de Deporte UCAM Universidad Católica de Murcia, Murcia Spain

^3^ Strength and Conditioning Society, Murcia, Spain

^4^ Independent Researcher

* Correspondence kspyrou@ucam.edu

**Abstract:** Futsal is an indoor sport characterized as a high-intensity intermittent modality with high physical, technical, and tactical demands (1). Furthermore, quantifying external load during futsal competition can provide objective data for the management of the athlete’s performance and late-stage rehabilitation, however, research in female futsal is very limited (2). This study aimed to report the match external load collected via wearable technology and analyze the differences time periods (i.e., halves 1st and 2nd half) in female futsal. Eight female futsal players competed in the 2nd Division of Spain used a GPS-accelerometer unit during all games of the 2022–2023 season. Player load (PL), PL·min^−1^, moderate- and high-intensity acceleration (ACC) and deceleration (DEC) data were collected. Linear Mixed Model used to analyze the differences between the halves via Jamovi software. During match, on average every player displayed values of: total PL 478 ± 126 a.u; PL·min^−1^: 10.4 ± 1.5 a.u; number of medium-intensity ACC 16 ± 6, high-intensity ACC 9 ± 5 and medium-intensity DEC 8 ± 3, high-intensity DEC 3 ± 3. Nonsignificant differences were found between halves. This study indicates that futsal players are exposed to great number of mechanical external loads, and perform a great number of ACC, and DEC. Coaches and sports scientists in female futsal are advised to implement high intensity exercises, with high number of ACC and DEC activities in the training sessions, and may use these reference values to design specific training and return-to-play plans.

**Keywords:** soccer; countermovement jump; isometric strength; sensitivity

**Funding:** This research received no external funding.

**References**

1. Spyrou, K.; Freitas, T.T.; Marín-Cascales, E.; Alcaraz, P.E. Physical and physiological match-play demands and player characteristics in futsal: a systematic review. *Front Psychol* **2020**, *11*, 569897.
2. Armendáriz, M.L.P.; Spyrou, K.; Alcaraz, P.E. Match demands of female team sports: a scoping review. *Biol Sport* **2023**, *41*, 175–199.

### 2.53. Activation of NFATc1 after Exercise in Human Skeletal Muscle: Preliminary Results

Angeliki-Nikoletta Stasinaki ^1,^*, Spyridon Methenitis ^1^, Thomas Mpampoulis ^1^, George Papadimas ^2^, Constantinos Papadopoulos ^2^ and Gerasimos Terzis ^1^

^1^ Sports Performance Laboratory, School of Physical Education & Sports Science, National and Kapodistrian University of Athens, 17237 Athens, Greece; agstasin@phed.uoa.gr

^2^ A’ Neurology Clinic, Aiginition Hospital, Medical School, National and Kapodistrian University of Athens, 11528 Athens, Greece

* Correspondence: agstasin@phed.uoa.gr

**Abstract:** It has been hypothesized, through animals’ studies, that depending on the type of “exercise” performed there is a specialization as to which nuclear factor of activated T-cells (NFAT) isoforms will function, migrate and enter the muscle nuclei, so that the different myosin heavy chain (MHC) isoforms are expressed as appropriate with the exercise-induced stimulus. However, to date this mechanism has not been studied in human skeletal muscle, following a session of physical exercise. The purpose of this study was to investigate the migration of the c1 isoform of NFAT in the nuclei of muscle cells after either one session of endurance exercise or one session of power exercise, in humans. Eight young, healthy males (age: 20.8 ± 0.3 years, body mass: 76.4 ± 7.7 kg, height: 180.2 ± 5.5 cm) were divided into two groups and performed a session of either aerobic exercise (30 min at 70% of maximum heart rate) or power training (16 countermovement jumps and then 16 drop jumps from a 32 cm box). Before and 50 min after the end of the training sessions, muscle biopsies were collected from vastus lateralis of the non-dominant limb. Muscle samples were ana-lyzed with immunohistochemistry, for fiber type composition and to identify the c1 isoform of NFAT within muscle nuclei. Fifty minutes after the end of each exercise session, no significant difference was found either in the number of muscle nuclei with NFATc1 (Power: Before 10.8 ± 13.0/After 8.8 ± 12.9; *p* = 0.463, Aerobic: Before 5.0 ± 1.0/After 6.7 ± 2.3; *p* = 0.789), nor in the ratio of the number of muscle nuclei with NFATc1/total number of cores (Power: Before 0.021 ± 0.008/After 0.017 ± 0.022; *p* = 0.397, Aerobic: Before 0.012 ± 0.007/After 0.021 ± 0.006; *p* = 0.285). No differences were found between groups at any time-point. In conclusion, the results of the present study indicate that 50 min after either aerobic or power exercise, there is no significant activation and migration of the c1 isoform of the NFAT transcription factor in muscle nuclei in human muscle tissue.

**Keywords:** muscle fiber type distribution; aerobic exercise; resistance exercise; power exercise.

**Funding:** This research is co-financed by Greece and the European Union (European Social Fund-ESF) through the Operational Programme «Human Resources Development, Education and Lifelong Learning» in the context of the project “Reinforcement of Postdoctoral Researchers—2nd Cycle” (MIS-5033021), implemented by the State Scholarships Foundation (ΙΚΥ).

**References**

1. Calabria, E.; Ciciliot, S. NFAT isoforms control activity-dependent muscle fiber type specification. *Proc Natl Acad Sci USA* **2009**, *106*, 13335–13340.
2. Blaauw, B.; Schiaffino, S. Mechanisms modulating skeletal muscle phenotype. *Compr Physiol* **2013**, *3*, 1645–87.
3. Liu, Y.; Shen, T. Signaling pathways in activity-dependent fiber type plasticity in adult skeletal muscle. *J Muscle Res Cell Motil* **2005**, *26*, 13–21.

### 2.54. Effect of Superimposed Local Vibration on Strength Production of the Quadriceps

Tom Timbert ^1,2,^*, Nicolas Amiez ^1,2^, Carole Cometti ^1,2^ and Christos Paizis ^1,2^

^1^ CAPS, U1093 INSERM, Sport Science Faculty, University of Bourgogne Franche-Comté, 21078 Dijon, France; tom.timbert@gmail.com; Nicolas.amiez@u-bourgogne.fr; carole.cometti@u-bourgogne.fr; christos.paizis@u-bourgogne.fr

^2^ Center of Performance Expertise, Sport Science Faculty, 3 Allée des Stades Universitaires, BP 27877, CEDEX, University of Bourgogne Franche-Comté, 21078 Dijon, France

* Correspondence: tom.timbert@gmail.com

**Abstract:** In recent years, vibration stimulation at rest had been widely investigated, and used to improve strength conditioning (1,2). Although, little is known about the effect of superimposed local vibration (SLV) on strength resistance. Thus, the aim of this study was to investigate the effect of SLV (100 Hz, 0.5 mm) on the quadricipital tendon during a fatiguing task. Each of the eleven participants had to realize, in three different sessions, twenty isometric knee extensions at 70% of the maximal strength with either; SLV at each contraction (VIBS_uperimposed_), SLV from when the participant could not sustain 70% of his maximal strength, to the end of the protocol (VIBF_atigue_) or control without vibration (CON). SLV was applied only during the 15 s of contraction and not during the 15 s rest time. Maximal strength, low and high frequency fatigue ratio (LFF/HFF) as well as volition activation level (VAL) has been tested before and after the fatiguing protocol (Post 0 min, 10 min, and 30 min). During the contractions, the torque and the electromyographic activity (EMGA) of the Vastus Medialis (VM), Vastus Lateralis (VL), Rectus Femoris (RF) have been studied. For the fatiguing task, the torque had been reported to a percentage of the maximal strength. Compared to CON, our results showed a significant increase for VIBF_atigue_ total mean torque (*p* = 0.018) but not for VIBS_uperimposed_ (*p* = 0.746), respectively (57.5 ± 13.5% and 56.5 ± 13.0% and CON: 55.4 ± 12.3%). ANOVAs did not reveal any significant differences for the EMGA for VM (*p* > 0.062), RF (*p* > 0.335) nor VL (*p* > 0.066) during the fatiguing task. No significant differences were found between *groups* in PRE and POST test for maximal strength, LFF/HFF and VAL (*p* > 0.14). However, significant decrease was found in *time* (*p* < 0.001) between PRE and POST0 and POST10 for maximal strength. The ratio LFF/HFF decreased significantly between PRE and POST0, POST10 and POST30 (*p* < 0.001). Our results suggest that SLV is an effective method to enhance strength during a fatiguing task when applied when the force is decreasing as fatigue appears. Furthermore, this increase in strength is not associated with increased loss of strength or fatigue markers compared to without SLV.

**Keywords:** local vibration; strength; neuromuscular; fatigue.

**Funding:** This research received no external funding.

**References**

1. Souron R.; Besson T.; Millet G.Y.; Lapole T. Acute and chronic neuromuscular adaptations to local vibration training. *Eur J Appl Physiol **2017**, 117*, 1939–1964.
2. Fattorini L.; Rodio A.; Pettorossi V.E.; Filippi G.M. Is the Focal Muscle Vibration an Effective Motor Conditioning Intervention? A Systematic Review. *J Funct Morphol Kinesiol* **2021**, *6*, 39.

### 2.55. Lean Body Mass, Muscle Architecture and Performance in Pow-Erlifters at Preseason and at Competition

Konstantinos Tromaras ^1^, Nikos Zaras ^2^, Aggeliki Stasinaki ^1^, Thomas Mpampoulis ^1^, Gerasimos Terzis ^1,^*

^1^ Sports Performance Laboratory, School of Physical Education and Sports Science, National and Kapodistrian University of Athens, Greece; spl@phed.uoa.gr

^2^ Human Performance Laboratory, Department of Life Sciences, School of Life and Health Sciences, University of Nicosia, Cyprus

* Correspondence: k.tromaras@phed.uoa.gr

**Abstract:** Lean body mass (LBM) is correlated with powerlifting performance (1). However, it remains uncertain whether training-induced changes in lean body mass may predict performance changes in powerlifting athletes. The purpose of the study was to investigate the correlation between changes in lean body mass and changes in powerlifting performance, before and after a short preparation period towards a major powerlifting event. Eleven well-trained powerlifters (8 males, 3 females, age 32 ± 11 years, height 171 ± 12.3 cm, weight 90.9 ± 21.6 kg), participated in the study. Athletes followed 12 weeks of training aiming to maximize their performance at the national competition event. Maximal strength (1-RM) in squat, bench press, and deadlift, body composition (dual x-ray absorptiometry), quadriceps cross sectional area and vastus lateralis muscle architecture (ultrasonography), were measured before and after the training period. Maximal strength in squat increased by 5.8 ± 7.0% (*p* < 0.05), bench press increased by 4.9 ± 9.8% (*p* = 0.05) and deadlift increased by 8.3 ± 16.7% (*p* < 0.05). Lean body mass of trunk increased by 3.1 ± 4.7% (*p* < 0.05). No alterations were observed for vastus lateralis muscle architecture after the training period (*p* > 0.05). Percentage changes in 1-RM in squat, bench press and deadlift, were highly correlated with percentage changes in LBM of the legs (r = 0.81, *p* = 0.003) and total body LBM (r = 0.63 and r = 0.69, *p* < 0.05), respectively. Also, changes in 1-RM in squat after the training intervention were significantly associated with changes in quadriceps cross sectional area (r = 0.67, *p* < 0.05). These results suggest that individual changes in lean body mass due to systematic resistance training toward a competition may predict increases in 1-RM performance in trained powerlifters.

**Keywords:** body composition; ultrasonography; resistance training; muscle strength.

**Funding:** This research received no external funding.

**References**

1. Ferland, P.M.; St-Jean Miron, F.; Laurier, A.; Comtois, A.S. The relationship between body composition measured by dual-energy X-ray absorptiometry and maximal strength in classic powerlifting. *J Sports Med Phys Fitness* **2020**, *60*, 407–16.

### 2.56. Physiological Responses in Winter Swimmers after a Swimming Session

**Nektaria Tseklima ^1^ and Ionas Papassotiriou ^1^**

^1^ Department of Physical Education and Sports Science, French College of Greece-IdEF College

**Abstract:** Winter swimming is a popular activity among swimmers, with some studies providing evidence of possible health benefits. However, there is a lack of comprehensive research regarding the acute physiological responses associated with winter swimming. Therefore, the aim of this study was to examine the physiological responses after winter swimming in a sample of amateur swimmers in Greece. The study involved 24 amateur winter swimmers aged 57.0 ± 11.7 years, who performed a free session of winter swimming during March with 15.6 °C in the water. Demographic information and anthropometrics, including weight, height, and body mass index were recorded in all participants. Body temperature from ear measurements, oxygen saturation, heart rate and subjective sensation of heat were recorded before and after swimmers entered the water. The results showed a significant decrease in body temperature, oxygen saturation and thermal sensation after swimming (*p* < 0.01), while correlation analysis revealed a positive significant relation between age and thermal sensation after swimming (rs = 0.405, *p* = 0.050). Also, a statistically significant difference was found between men and women in body temperature after swimming (Mann-Whitney U = 28.500, *p* = 0.010), without any significant difference in the other physiological parameters (*p* > 0.05); however, the mean BMI was 24.5 ± 4.9 kg/m^2^ for women and 26.8 ± 3.9 kg/m^2^ for men. The results of this study showed that swimming in cold water not only reduces body temperature, but also alters oxygen delivery, possible due to vasoconstriction and oxygen usage. In addition, older swimmers found to have a resistance to thermal sensation after swimming in cold water. Also, sex differences in temperature regulation or sensation may exist, since men reported higher values of thermal sensation than women, which could be partially explained by the fact that in this sample men were overweight, while women had normal weight.

**Funding:** This research received no external funding.

### 2.57. Acute Effects of Fast vs. Slow Tempo of Movement with Equated Time under Tension on Bench Press Throw Performance

Athanasios Tsoukos ^1,^*, Gerasimos Terzis ^1^ and Gregory C. Bogdanis ^1^

^1^ School of P.E. and Sport Science, National and Kapodistrian University of Athens, Greece; atsoukos@phed.uoa.gr

* Correspondence: atsoukos@phed.uoa.gr

**Abstract:** This study examined the acute effect of the bench press exercise executed with fast or slow tempo and equal time under tension (TUT) on bench press throw performance. Eleven men (age: 23.5 ± 5.4 years, height: 1.79 ± 0.04 m, body mass: 79.1 ± 6.4 kg, maximum strength 1-RM: 91.0 ± 12.0 kg) with at least 2 years of experience in the bench press (conditioning activity-CA) performed three conditions at 70% of 1-RM on a Smith machine in a randomized and counterbalanced order 5–7 days apart: (a) 2 sets of 6 repetitions with maximum velocity (FAST) or (b) 2 sets of 3 repetitions at half maximum velocity (SLOW) or (c) rest (CTRL). Before and at 0.75, 4, 8 and 12 min after the bench press exercise the participants performed three bench press throws (BPTs). Mean velocity of all the repetitions of the conditioning bench press as well as the BPTs were assessed with a linear position transducer. Peak force of the acceleration phase of the bench press (average peak forces of the 6 and 3 repetitions of the FAST and SLOW conditions respectively) was calculated based on measures obtained by an accelerometer. The tempo of the SLOW condition was determined during a preliminary session. During the main conditions a metronome used to pace the exact tempo of the SLOW condition. Total TUT of the FAST and SLOW conditions was equal between conditions (FAST: 12.9 ± 1.5 s vs. SLOW: 11.8 ± 1.3 s; *p* > 0.05). Peak force of the 1st and 2nd set of the bench press (CA) were 43.0 ± 28.9% and 52.0 ± 30.0% higher during the FAST compared with the SLOW (*p* < 0.001). Two-way ANOVA showed that mean velocities of the BPTs were greater at 4, 8 and 12 min compared with pre in both FAST and SLOW conditions (*p* < 0.001) and both were higher compared with the CTRL at the same time points (*p* < 0.001). No differences were observed between the experimental conditions (*p* > 0.05). Fast and slow tempo of movement with equated time under tension exhibit a similar acute improvement of BPT performance. This indicate that load, recovery time and the specificity of movement determine the acute improvement of performance and not the movement tempo.

**Keywords:** postactivation performance enhancement**;** resistance exercise; movement velocity; power training.

**Funding:** This research received no external funding.

**References**

1. Wilk, M.; Krzysztofik, M.; Drozd, M.; Zajac, A. Changes of Power Output and Velocity During Successive Sets of the Bench Press With Different Duration of Eccentric Movement. *Int J Sports Physiol Perform* **2020**, *15*, 162–167.
2. Wilk, M.; Jarosz, J.; Krzysztofik, M.; Filip-Stachnik, A.; Bialas, M.; Rzeszutko-Belzowska, A.; Zajac, A.; Stastny, P. Contrast Tempo of Movement and Its Effect on Power Output and Bar Velocity During Resistance Exercise. *Front Physiol* **2021**, *11*, 629199.

### 2.58. Validity of a Novel App for Estimating Jump Height from Audio Recordings

Amilton Vieira ^1^, Victor O. C. Macedo ^1^ and Roberto S. Baptista ^1,^*

^1^ Strength Training Laboratory, Faculty of Physical Education, University of Brasilia, Brazil

* Correspondence: amiltonvieira@unb.br

**Abstract:** Mobile apps can accurately and reliably measure jump height from slow-motion captured video, but the apps require a time-consuming manual identification of take-off and landing frames (1). We investigated the concurrent validity of a novel app automatically estimating jump height from audio recordings. Fifty university-aged participants (26 ± 9 years) jumped on a force platform (*i.e.,* criterion) while a smartphone recorded the jump sound. We compared the jump height estimates from audio against force platform applying flight time (FT) and impulse-momentum “gold-standard” (*J*) methods. Validity was determined by regression analysis, Pearson product-moment correlation coefficient (*r*), and standard error of estimates (SEE). Almost perfect correlations exist between the audio and FT (*r* = 0.99, SEE = 1.4 cm) and *J* (*r* = 0.96, SEE = 2.2 cm). The results show that jump height can be accurately and quickly measured from audio recording using an automatized mobile app.

**Keywords:** mobile application; exercise test; functional physical fitness.

**Funding:** This research was partially funded by Fundação de Apoio à Pesquisa do Distrito Federal (FAPDF) and Fundação de Apoio à Pesquisa (FUNAPE).

**References**

1. Vieira, A.; Ribeiro, G.L.; Macedo, V.; de Araújo Rocha Junior, V.; Baptista, R.S.; Gonçalves, C.; Cunha, R.; Tufano, J. Evidence of validity and reliability of Jumpo 2 and MyJump 2 for estimating vertical jump variables. *PeerJ* **2023**, *11*, e145585.

### 2.59. Can Dynamic Stretching Velocity Influence Acute Muscle Strength Adaptations?

Denis Vieira ^1,2,^*, Martim Bottaro ^2^, Marion Hitier ^1^, João Durigan ^3^ and Nicolas Babault ^1^

^1^ Centre d’expertise de la Performance & INSERM UMR1093-CAPS, Université de Bourgogne, UFR des Sciences du Sport, Dijon, France; denisclvieira@hotmail.com

^2^ Laboratório de Pesquisa em Treinamento de Força, Universidade de Brasília, Faculdade de Educação Física, Brasília, Brasil

^3^ Laboratório de Plasticidade Musculotendínea, Universidade de Brasília, Programa de Pós-Graduação em Ciências da Reabilitação, Brasília, Brasil

* Correspondence: denisclvieira@hotmail.com; Tel.: +33-03-80-39-67-89

**Abstract:** Dynamic stretching (DS) has been suggested as a warm-up strategy to improve muscle performance acutely. However, the impact of DS movement velocity on the magnitude of these improvements still needs clarification. Thus, this study aimed to verify the effects of DS velocity on isometric muscle strength. Fourteen individuals (11 men and 3 women, age 27 ± 4.4 years; mass: 74.9 ± 13.1 kg; height: 175.7 ± 11 cm) performed four experimental sessions in random order: (1) DS at fast velocity (90 beeps per minute-bpm); (2) DS at moderate velocity (70 bpm); (3) DS at slow velocity (50 bpm); (4) Control Session. The protocols were performed on plantar flexors of the right ankle with individuals lying sideway on the isokinetic machine. The protocols consisted of 4 sets of 10 repetitions with a rest interval between sets of 30 s. Muscle strength was assessed with a single maximal voluntary isometric contraction of 5 s performed before (PRE), immediately after (POST), 7 (P07), and 14 (P14) minutes after each protocol. The mean and standard deviations of the isometric muscle strength assessments (N/m) in the PRE, POST, P07, and P14 were respectively: (1) 103.22 ± 32.77, 99.78 ± 30.76, 95.70 ± 34.52, and 98.35 ± 33.41 for the Control Session; (2) 102.82 ± 39.86, 105.69 ± 38.19, 99.76 ± 35.26, and 104.10 ± 36.04 for Slow DS; (3) 101.00 ± 30.56, 105.25 ± 38.72, 104.91 ± 39.26, and 103.54 ± 39.35 for moderate DS; (4) 98.55 ± 29.83, 104.29 ± 35.78, 102.43 ± 36.94, and 100.99 ± 33.30 for Fast DS. The Repeated Measures ANOVA reported neither significant effect for time (*p* = 0.356) nor condition (*p* = 0.586) for isometric muscle strength. Moreover, there was no interaction between time and condition (*p* = 0.285). Therefore, DS independent of the velocity performed did not influence muscle strength production.

**Keywords:** muscle performance; force; maximal voluntary contraction.

**Funding:** The study was supported by the Centre d’Expertise de la Performance from the Université of Bourgogne, by the Region Bourgogne Franche- Comteé (2020Y-22065 and 2022Y-13186), and by the National Council for Scientific and Technological Development–CNPq (200391/2022-4).

**References**

1. Vieira, D.C.L.; Opplert, J.; Babault, N. Acute effects of dynamic stretching on neuromechanical properties: an interaction between stretching, contraction, and movement. *Eur J Appl Physiol* **2021**, *121*, 957–967.
2. Opplert, J.; Babault, N. Acute effects of Dynamic Stretching on Muscle Flexibility and Performance: An Analysis of the Current Literature. *Sports Med* **2018**, *48*, 299–325.

